# Supplementary material for: Impact of an Acceptance and Commitment Therapy programme on HbA1c, self-management and psychosocial factors in adults with type 1 diabetes and elevated HbA1c levels: a randomised controlled trial
Source: BMJ Open. 2023 Dec 14;13(12):e072061. doi: 10.1136/bmjopen-2023-072061 (PMC10729111; doi:10.1136/bmjopen-2023-072061)
Supplement: Supplementary data [file bmjopen-2023-072061supp001.pdf]

Supplementary Table 1: Estimates of the adherence among the instructors (n=2) to the ACT programme.

	Group A (n=5) s 3 median (range)	Instructors s 3 median (range)	Group A (n=5) s 7 median (range)	Instructors s 7 median (range)	Group B (n=3) s 3 median (range)	Instructors s 3 median (range)
Review of preceding sessions	8 (6-9)	10 (10-10)	9 (9-10)	8 (8-8)	7 (6-9)	9 (9-9)
Feedback on homework	7 (5-10)	7.5 (7-8)	9 (8-10)	6.5 (6-7)	8 (7-10)	8 (7-9)
The Compass of life	10 (7-10)	7.5 (7-8)	9 (9-10)	8 (8-8)	9 (7-10)	9.5 (9-10)
Specific exercise	9.5 (8-10)	8.5 (8-9)	10 (8-10)	9 (9-9)	9 (6-10)	9.5 (9-10)

Estimations on a scale 1-10 (0= “this has not been discussed/brought up”, to 10 = “This was covered thoroughly”.)
Examples of items from the adherence evaluation. s 3: session 3, s 7: session 7

Supplementary Table 2: Estimates of the adherence among participants in two groups to the ACT programme.

	Group A (n=5) s 3 median(range)	Group A (n=5) s 7 median(range)	Group B (n=3) s 3 median(range)
Made changes in life[1]	2 (1-2)	2.5 (2-3)	2 (2-3)
App use (mindfulness)[2]	0 (0-2)	1 (0-1)	2 (0-10)
Mindfulness daily life[3]	3 (2-4)	3.5 (2-4)	4 (3-4)

1. Have you since the course started, decided to change something/take steps in your life direction? 1=No, 2=I have decided and made a small step/change,3=I have decided and made several changes/steps.

2. How many times per week did you practise mindfulness with the app “I´m here now”?

3. How often do you practise mindfulness in your daily life? 1=Never,2=Seldom, 3=Once a week/sometimes,4=Once per day, 5=Several times a day

s 3: session 3, s 7: session 7

Supplementary Table 3: Observed mean value at each time point for outcome measurements and linear mixed model analysis with testing for interaction effects of time and ACT intervention on HbA1c, PAID, DASS21 and AAQ-II.

								Mixed model analysis with interaction		
	**Week 0**	**Week 8**	**Week 14**	**6 months**	**12 months**	**24 months**		**Fixed effects**	**Estimate (95% CI)**	**Interaction effect χ^2 (p value)**
**HbA1c** mmol/mol										
ACT								Intercept	75.22 (71.43, 79.01)	
N	43	27	21	15	16	15		ACT	-3.71 (-8.90, 1.49)	
Mean (SD)	71.4 (10.1)	70.1 (8.9)	69.4 (8.3)	63.6 (9.5)	64.6 (8.4)	59.6 (8.0)		Time	-0.05 (-0.09, -0.01)	
Control								ACT*time	-0.02 (-0.07, 0.03)	
N	38	20	21	15	17	11				
Mean (SD)	75.9 (15.4)	75.7 (14.4)	72.4 (11.9)	64.6 (5.7)	70.2 (13.3)	70.5 (18.6)				
P-value	0.315	0.281	0.227	0.677	0.181	0.132				0.49 (0.485)
**PAID**										
ACT								Intercept	43.52 (37.78, 49.29)	
N	39	24	21	19	17	17		ACT	-9.40 (-17.36, -1.46)	
Mean (SD)	36.6 (18.1)	32.6 (19.7)	28.7 (18.7)	31.1 (18.5)	28.6 (16.0)	27.4 (18.6)		Time	-0.05 (-0.10, -0.01)	
Control								ACT*time	-0.01 (-0.08, 0.05)	
N	32	25	25	21	19	16				
Mean (SD)	42.9 (15.9)	39.8 (20.7)	41.6 (19.2)	34.9 (20.9)	36.2 (16.6)	33.4 (17.7)				
P value	0.229	0.322	0.034	0.533	0.188	0.279				0.22(0.642)
**DASS-S**										
ACT								Intercept	16.53 (13.63, 19.44)	
N	39	24	21	19	17	17		ACT	-4.90 (-8.92, -0.89)	
Mean (SD)	12.5 (8.4)	12.5 (9.2)	9.8 (8.3)	12.8 (10.4)	9.4 (6.3)	10.7 (6.7)		Time	-0.01 (-0.03, 0.02)	
Control								ACT*time	-0.01 (-0.04, 0.03)	
N	32	25	25	21	19	16				
Mean (SD)	16.8 (11.1)	14.5 (10.7)	15.8 (9.7)	13.0 (10.9)	14.3 (11.3)	14.1 (10.7)				

P value	0.141	0.534	0.036	0.978	0.226	0.458				0.11(0.743)
**DASS-A**										
ACT								Intercept	7.54 (5.44, 9.64)	
N	39	24	21	19	17	17		ACT	-1.06 (-3.97, 1.85)	
Mean (SD)	6.8 (6.8)	5.8 (7.1)	5.4 (6.3)	7.3 (8.3)	4.2 (4.0)	4.6 (7.9)		Time	0.01 (-0.01, 0.03)	
Control								ACT*time	-0.02 (-0.05, 0.01)	
N	32	25	25	21	19	16				
Mean (SD)	8.2 (7.1)	6.6 (7.0)	6.9 (6.0)	6.5 (7.0)	8.0 (6.7)	7.1 (6.2)				
P value	0.302	0.460	0.283	0.763	0.042	0.063				1.97(0.161)
**DASS-D**										
ACT								Intercept	13.10 (9.87, 16.35)	
N	39	24	21	19	17	17		ACT	-2.53 (-7.03, 1.95)	
Mean (SD)	11.7 (11.4)	10.9 (11.0)	6.9 (9.4)	11.6 (12.8)	6.0 (7.4)	10.0 (7.5)		Time	0.02 (-0.01, 0.06)	
Control								ACT*time	-0.03 (-0.08, 0.01)	
N	32	25	25	21	19	16				
Mean (SD)	12.6 (10.3)	11.3 (9.0)	14.1 (10.0)	11.1 (11.7)	14.8 (11.3)	14.1 (10.5)				
P value	0.486	0.672	0.015	0.989	0.011	0.261				2.00(0.157)
**AAQ**										
ACT								Intercept	17.69 (15.34, 20.05)	
N	39	24	21	19	17	17		ACT	-1.95 (-5.20, 1.30)	
Mean (SD)	16.3 (8.4)	15.8 (8.5)	14.2 (8.3)	15.0 (9.3)	11.9 (7.8)	12.5 (7.8)		Time	0.02 (0.00, 0.04)	
Control								ACT*time	-0.05 (-0.08, -0.02)	
N	32	25	25	21	19	16				
Mean (SD)	18.1 (6.1)	16.0 (7.0)	17.4 (7.3)	16.7 (7.2)	17.9 (7.9)	18.9 (7.8)				
P value	0.325	0.749	0.095	0.356	0.016	0.013				12.63 (<0.001)

*DASS-S-stress, DASS-A-anxiety, DASS-D-depression

ACT, acceptance and commitment therapy; SD, standard deviation; CI, confidence interval; HbA1c, glycated haemoglobin; PAID, Problem Areas in Diabetes Scale; DASS, Depression, Anxiety, Stress Scale; AAQ, Acceptance and Action Questionnaire.

Supplementary Table 4: Observed mean value at each time point for outcome measurements and linear mixed model analysis with testing for interaction effects of time and ACT intervention on HFS, AADQ, MANSA and SDSCA

**HFS-B**										
ACT								Intercept	20.27 (18.61, 21.93)	
N	39	24	21	19	17	17		ACT	-3.14 (-5.44, -0.85)	
Mean (SD)	18.1 (4.9)	15.8 (5.2)	15.7 (4.6)	15.9 (4.4)	15.4 (4.5)	16.7 (6.8)		Time	-0.02 (-0.03, 0.00)	
Control								ACT*time	0.01 (-0.01, 0.03)	
N	32	25	25	21	19	16				
Mean (SD)	20.4 (5.3)	19.5 (4.9)	19.5 (6.7)	18.7 (6.0)	18.7 (5.8)	19.6 (5.3)				
P-value	0.030	0.013	0.021	0.162	0.177	0.139				0.55 (0.457)
**HFS-W**										
ACT								Intercept	17.00 (14.02, 19.99)	
N	39	24	21	19	17	17		ACT	-2.06 (-6.20, 2.05)	
Mean (SD)	16.7 (10.2)	14.2 (8.7)	12.5 (6.6)	12.0 (6.7)	11.6 (6.5)	12.6 (7.7)		Time	-0.01 (-0.04, 0.01)	
Control								ACT*time	-0.01 (-0.04, 0.02)	
N	32	25	25	21	19	16				
Mean (SD)	16.4 (9.3)	14.3 (8.8)	16.1 (8.7)	13.5 (11.4)	14.1 (8.7)	13.5 (8.7)				
P-value	0.986	0.888	0.182	0.957	0.526	0.843				0.65 (0.420)
**AADQ**										
ACT								Intercept	53.40 (50.50, 56.30)	
N	39	24	21	19	17	17		ACT	4.80 (0.80, 8.82)	
Mean (SD)	57.4 (8.8)	58.7 (7.6)	59.6 (7.7)	59.0 (9.2)	62.1 (8.5)	61.2 (8.3)		Time	0.03 (0.01, 0.05)	
Control								ACT*time	0.01 (-0.02, 0.04)	
No.	32	25	25	21	19	16				

Mean (SD)	52.6 (11.3)	54.3 (11.3)	54.5 (9.6)	58.3 (7.8)	56.5 (7.1)	57.8 (5.9)				
P value	0.049	0.207	0.047	0.560	0.022	0.073				0.34(0.562)
**MANSA**										
ACT								Intercept	4.65 (4.38, 4.91)	
N	39	24	21	19	17	17		ACT	0.44 (0.07, 0.80)	
Mean (SD)	5.0 (0.9)	5.1 (0.8)	5.3 (0.8)	5.1 (0.9)	5.3 (0.6)	5.3 (0.9)		Time	0.00 (0.00, 0.00)	
Control								ACT*time	0.00 (0.00, 0.01)	
N	32	25	25	21	19	16				
Mean (SD)	4.7 (0.7)	4.7 (1.0)	4.5 (0.9)	4.8 (0.9)	4.8 (0.9)	4.5 (0.8)				
P value	0.093	0.118	0.006	0.266	0.038	0.015				5.79(0.016)
**SDSCA-GD**										
ACT								Intercept	4.14 (3.61, 4.67)	
No.	39	24	21	19	17	17		ACT	0.75 (0.02, 1.48)	
Mean (SD)	5.0 (1.6)	4.7 (1.8)	4.5 (1.7)	4.9 (1.6)	5.1 (1.5)	5.1 (1.5)		Time	0.01 (0.00, 0.01)	
Control								ACT*time	0.00 (-0.01, 0.01)	
No.	32	25	25	21	19	16				
Mean (SD)	4.1 (1.9)	3.8 (2.1)	4.4 (1.8)	4.9 (1.4)	4.9 (1.8)	4.7 (1.6)				
P value	0.062	0.104	0.722	0.743	0.784	0.388				0.01(0.924)
**SDSCA- SD**										
ACT								Intercept	3.66 (3.18, 4.15)	
N	39	24	21	19	17	17		ACT	0.21 (-0.46, 0.88)	
Mean (SD)	3.8 (1.8)	4.1 (1.5)	3.6 (1.4)	4.5 (1.2)	4.2 (1.6)	4.1 (1.7)		Time	0.00 (-0.01, 0.00)	
Control								ACT*time	0.00 (0.00, 0.01)	
No.	32	25	25	21	19	16				

Mean (SD)	3.5 (1.6)	3.7 (1.6)	3.7 (1.6)	4.2 (1.5)	3.7 (1.5)	3.6 (1.6)				
P value	0.313	0.415	0.706	0.405	0.425	0.269				1.37 (0.242)
**SDSCA-E**										
ACT								Intercept	2.62 (2.03, 3.22)	
No.	39	24	21	19	17	17		ACT	0.73 (-0.10, 1.55)	
Mean (SD)	3.2 (2.1)	4.2 (1.9)	3.2 (2.0)	3.8 (2.3)	4.1 (2.1)	3.6 (2.2)		Time	0.00 (0.00, 0.01)	
Control								ACT*time	0.00 (-0.01, 0.01)	
N	32	25	25	21	19	16				
Mean (SD)	2.5 (2.0)	2.7 (2.0)	2.5 (1.9)	2.9 (1.8)	3.2 (2.6)	2.9 (1.7)				
P value	0.174	0.010	0.186	0.253	0.245	0.536				0.10 (0.749)
**SDSCA -G**										
ACT								Intercept	5.37 (4.83, 5.91)	
N	39	24	21	19	17	17		ACT	0.84 (0.09, 1.59)	
Mean (SD)	6.1 (1.8)	6.3 (1.2)	6.3 (1.4)	6.6 (0.9)	6.9 (0.3)	6.8 (0.8)		Time	0.01 (0.00, 0.01)	
Control								ACT*time	0.00 (-0.01, 0.01)	
N	32	25	25	21	19	16				
Mean (SD)	5.4 (2.5)	5.5 (2.3)	5.7 (2.2)	6.0 (1.7)	6.0 (1.9)	6.7 (0.8)				
P value	0.423	0.300	0.613	0.265	0.117	0.924				0.23 (0.634)

ACT, acceptance and commitment therapy; SD, standard deviation; CI, confidence interval; HbA1c, glycated haemoglobin; Scale; HFS, Hypoglycaemia Fear Survey (HFS- W-Worry, HFS-B- Behaviour); AADQ, Acceptance Action Diabetes Questionnaire; MANSA, Manchester Short Assessment of Quality of life; SDSCA, Summary of Diabetes Self-Care Activities (SDSCA-GD-general diet, SDSCA-SD-specific diet, SDSCA-E-Exercise, SDSCA-G-Glucose monitoring).

Supplementary Table 5: ACT exposure (number of sessions attended) and proportion of participants with HbA1c measurement in each timepoint

**ACT exposure**	**Frequency**<br>Intervention group<br>(n =43)	**Frequency**<br>Control group<br>(n=38)
Zero sessions	8	-
One session	3	-
Two sessions	5	-
Three sessions	7	-
Four sessions	1	-
Five sessions	3	-
Six sessions	9	-
Seven sessions	7	-
**HbA1c measurement**		
Week 8, n (%)	27 (62.8)	20 (52.6)
Week 14, n (%)	21 (48.8)	21 (55.3)
6 months, n (%)	15 (34.9)	15 (39.5)
12 months, n (%)	16 (37.2)	17 (44.7)
24 months, (%)	15 (34.9)	11 (28.9)

ACT, acceptance and commitment therapy; HbA1c, glycated Haemoglobin.
