# Supplementary material for: Impact of an Acceptance and Commitment Therapy programme on HbA1c, self-management and psychosocial factors in adults with type 1 diabetes and elevated HbA1c levels: a randomised controlled trial
Source: BMJ Open. 2023 Dec 14;13(12):e072061. doi: 10.1136/bmjopen-2023-072061 (PMC10729111; doi:10.1136/bmjopen-2023-072061)
Supplement: Author's manuscript [file bmjopen-2023-072061.draft_revisions.pdf]

BMJ Open

BMJ Open is committed to open peer review. As part of this commitment we make the peer review history of every article we publish publicly available.

When an article is published we post the peer reviewers' comments and the authors' responses online. We also post the versions of the paper that were used during peer review. These are the versions that the peer review comments apply to.

The versions of the paper that follow are the versions that were submitted during the peer review process. They are not the versions of record or the final published versions. They should not be cited or distributed as the published version of this manuscript.

BMJ Open is an open access journal and the full, final, typeset and author-corrected version of record of the manuscript is available on our site with no access controls, subscription charges or pay-per-view fees (http://bmjopen.bmj.com).

If you have any questions on BMJ Open's open peer review process please email info.bmjopen@bmj.com

BMJ Open

Impact of an Acceptance and Commitment Therapy programme on HbA1c, self-management and psychosocial factors in adults with type 1 diabetes and elevated HbA1c levels: a randomised controlled trial

Journal:	*BMJ Open*
Manuscript ID	bmjopen-2023-072061
Article Type:	Original research
Date Submitted by the Author:	21-Jan-2023

Complete List of Authors:	Wijk, Ingrid ; Sophiahemmet University, Department of Health Promoting Science<br>Amsberg, Susanne; Marie Cedershiöld University, Department of Health Care Sciences<br>Johansson, Unn-Britt; Sophiahemmet University, Department of Health Promoting Science; Karolinska Institutet, Department of Clinical Sciences and Education, Södersjukhuset<br>Livheim, Fredrik; Karolinska Institutet, Department of Clinical Neuroscience<br>Toft, Eva; Karolinska Institutet, Department of Medicine, Huddinge; Karolinska Institutet, Department of Medicine, Ersta Hospital<br>Anderbro, Therese; Karolinska Institutet, Department of Clinical Sciences; Stockholm University, Department of Psychology

Keywords:	DIABETES & ENDOCRINOLOGY, Depression & mood disorders < PSYCHIATRY, PREVENTIVE MEDICINE

SCHOLARONE™
Manuscripts

I, the Submitting Author has the right to grant and does grant on behalf of all authors of the Work (as defined in the below author licence), an exclusive licence and/or a non-exclusive licence for contributions from authors who are: i) UK Crown employees; ii) where BMJ has agreed a CC-BY licence shall apply, and/or iii) in accordance with the terms applicable for US Federal Government officers or employees acting as part of their official duties; on a worldwide, perpetual, irrevocable, royalty-free basis to BMJ Publishing Group Ltd ("BMJ") its licensees and where the relevant Journal is co-owned by BMJ to the co-owners of the Journal, to publish the Work in this journal and any other BMJ products and to exploit all rights, as set out in our licence.

The Submitting Author accepts and understands that any supply made under these terms is made by BMJ to the Submitting Author unless you are acting as an employee on behalf of your employer or a postgraduate student of an affiliated institution which is paying any applicable article publishing charge ("APC") for Open Access articles. Where the Submitting Author wishes to make the Work available on an Open Access basis (and intends to pay the relevant APC), the terms of reuse of such Open Access shall be governed by a Creative Commons licence – details of these licences and which Creative Commons licence will apply to this Work are set out in our licence referred to above.

Other than as permitted in any relevant BMJ Author's Self Archiving Policies, I confirm this Work has not been accepted for publication elsewhere, is not being considered for publication elsewhere and does not duplicate material already published. I confirm all authors consent to publication of this Work and authorise the granting of this licence.

For peer review only

Impact of an Acceptance and Commitment Therapy programme on HbA1c, self-management and psychosocial factors in adults with type 1 diabetes and elevated HbA1c levels: a randomised controlled trial

Ingrid Wijk[1*], Susanne Amsberg[2], Unn-Britt Johansson[1,3], Fredrik Livheim[4], Eva Toft[5,6], Therese Anderbro[7,8]

[1]Department of Health Promoting Science, Sophiahemmet University, Stockholm, Sweden

[2]Department of Health Care Sciences, Marie Cederschiöld University, Stockholm, Sweden

[3]Department of Clinical Sciences and Education, Södersjukhuset, Karolinska Institutet, Stockholm, Sweden

[4]Department of Clinical Neuroscience, Karolinska Institutet, Stockholm, Sweden

[5]Department of Medicine, Huddinge, Karolinska Institutet, Stockholm, Sweden

[6]Department of Medicine, Karolinska Institutet, Ersta Hospital, Stockholm, Sweden

[7]Department of Clinical Sciences, Danderyd Hospital, Karolinska Institutet, Stockholm, Sweden

[8]Department of Psychology, Stockholm University, Stockholm, Sweden

For peer review only

*Corresponding author:

E-mail address: ingrid.wijk@shh.se (I. Wijk).

Sophiahemmet University

P O Box 5605

SE-114 86 Stockholm, Sweden

ABSTRACT

Objective: To evaluate the impact of an Acceptance and Commitment Therapy (ACT) programme, tailored for people living with type 1 diabetes, on HbA1c, self-management and psychosocial factors among individuals with HbA1c >60 mmol/mol compared with treatment as usual (TAU).

Setting: An endocrinologic clinic in Sweden.

Participants: In this randomised controlled trial, 81 individuals with type 1 diabetes, aged 18-70 years with HbA1c >60 mmol/mol, were randomly assigned to either an ACT group intervention or TAU. Exclusion criteria were pregnancy, severe untreated psychiatric illness, disease or medical treatment that may affect the glucose level and newly started insulin pump treatment. At the two-year follow-up, HbA1c was measured in 26 individuals.

Intervention: The ACT programme comprised seven 2-hour sessions held over 14 weeks and focused on acceptance of stressful thoughts and emotions, and to promote value-based committed action.

Outcomes: The primary outcome was HbA1c, and the secondary outcomes were measures of depression, anxiety, general stress, fear of hypoglycaemia, diabetes distress, self-care activities, psychological flexibility (general and related to diabetes) and quality of life. The primary endpoint was HbA1c two years after the intervention programme. Linear mixed models were used to test for an interaction effect between measurement time and group.

Results: Likelihood ratio test of nested models demonstrated no statistically significant interaction effect (chi-square=1, p=0.316) between measurement time and group regarding HbA1c. However, a statistically significant interaction effect (likelihood ratio test chi-square=12.63, p<0.001) was observed with improved scores on The Acceptance and Action Questionnaire in the intervention group after one and two years.

Conclusions: No statistically significant difference was found between the groups regarding the primary outcome measure, HbA1c. However, the ACT programme showed a persistent beneficial impact on psychological flexibility in the intervention group.

Trial registration: Trial registration number NCT02914496.

Strengths and limitations of this study:

- This study was developed using an interdisciplinary approach, with a randomised control trial design.
- The intervention is theory-based, with roots in behavioural research, and is tailored for living with diabetes.
- This research addresses psychological aspects of living with type 1 diabetes which has been requested by the diabetes community and in international guidelines.
- The drop-out rate in this study affects the possibility to draw conclusions from the findings.

For peer review only - http://bmjopen.bmj.com/site/about/guidelines.xhtml

For peer review only

BACKGROUND

Type 1 diabetes is an autoimmune mediated disease (1) with an increasing global incidence in young people under 20 years of age (2). Complications of type 1 diabetes involve life-threating conditions such as hypoglycaemia and ketoacidosis as well as micro- and macro vascular diseases (3). National and international guidelines recommend treatment targets for glycaemic level, individually adjusted, to prevent development of complications (4, 5). However, only a minority reach the treatment targets (6-8).

The pancreatic beta cell destruction in type 1 diabetes brings a life-long dependency on supplying exogenous insulin (3) and along with that a self-management posing high demands (9). Glucose monitoring, insulin adjustment and managing hypo- and hyperglycaemia are examples of self-care activities involved (10). In addition to the amount of ingested carbohydrates, several factors such as physical activity, hormones, stress and temperature affect the glycaemic level (10). Consequently, self-management requires knowledge and skills (11) and involves frequent decision-making (12). Emotional stress related to the management of diabetes, diabetes distress, persists over time (13) and is present among 20%–30% of people with type 1 diabetes (14). Furthermore, the prevalence of depression is up to three-fold higher among individuals living with type 1 diabetes (15). Moreover, depression in individuals with diabetes is linked with an increased risk of mortality (16). Several studies have found that psychological health problems and elevated HbA1c levels are associated (17-19). Consequently, there has been a call to address the psychological aspects of living with diabetes (20, 21).

Cognitive Behaviour Therapy (CBT) is focused on restructuring maladaptive thoughts to create more functional cognition and behaviours (22). CBT has shown positive effects on depression in a general population (23) as well as among individuals with diabetes (24). In a randomised controlled trial (RCT) by Amsberg et al., a CBT intervention improved HbA1c and reduced levels of depression and diabetes distress (25).

Acceptance and Commitment Therapy (ACT) expands upon CBT. The approach in ACT suggests that the presence of uncomfortable thoughts and feelings should be accepted and not supressed or modified owing to the fact that human life is difficult (26). Despite the existence of uncomfortable thoughts, functional behaviours could pave the way to a life in accordance with a person's values and goals. Being present and aware, and at the same time developing functional behaviours adapted in the current context, is referred to as psychological flexibility (27). Moreover, psychological flexibility, which ACT aims to increase (28), is suggested to be a key prerequisite to psychological health (29). Previous studies have supported treatment effects of ACT for different mental and somatic health problems (30). With its focus on acceptance of discomfort, ACT has also been argued to be especially suitable for long-term conditions where distress can be a realistic factor (31). Furthermore, a recent study found that greater psychological flexibility in adults with type 1 diabetes was associated with less distress and more beneficial HbA1c (32). A meta-analysis concluded that ACT interventions may improve the ability of self-care, psychological flexibility and HbA1c in type 2 diabetes (33). Within the scope of type 1 diabetes, few ACT interventions for adults have been evaluated. A feasibility study of a single-arm trial involving individuals with type 1 diabetes and comorbid eating disorder indicated positive effects on psychological flexibility and diabetes distress, in addition to being feasible and improving eating disorder symptoms. Furthermore, an RCT feasibility study of an ACT-based intervention provided in webformat included individuals with type 1 and

For peer review only - http://bmjopen.bmj.com/site/about/guidelines.xhtml

type 2 diabetes. A reduction in diabetes distress was seen in the intervention group. However, the dropout rate was 57% during treatment (34).

In summary, type 1 diabetes is a severe disease with an increased prevalence of impaired psychological health with treatment targets reached by only a minority. Previous research regarding ACT and diabetes has mainly focused on type 2 diabetes. Our hypothesis was that an ACT programme would lead to improvements in glycaemic and psychosocial outcomes in an adult population with type 1 diabetes and prolonged elevation of HbA1c. Thus, we conducted an RCT aiming to evaluate the impact of an ACT programme, tailored for people living with type 1 diabetes, on HbA1c, self-management and psychosocial factors.

METHODS

Study design and participants

This study was a longitudinal two-arm RCT conducted at an endocrinologic clinic in Sweden. The intervention group participated in an ACT programme and the control group received treatment as usual (TAU) only. The endocrinology clinic population comprised adults (> 18 years) with type 1 diabetes and included urban and suburban residents. A study protocol has been published where a more detailed description of the method and the intervention is provided (35).

Eligible participants were identified by extracting data from the Swedish National Diabetes Register (NDR). Individuals who met the inclusion criteria: type 1 diabetes with a duration of at least 2 years, aged 18–70 years and HbA1c > 60 mmol/mol on two occasions during preceding year, were assessed in a random order (generated by computer). An additional inclusion criterion was the ability to speak, read and write in Swedish. Sequentially, patient records were reviewed for information on exclusion criteria. The following criteria were applied: severe, untreated mental illness, disease or treatment that can affect glucose level (e.g., steroid treatment, thyrotoxicosis, planned bariatric surgery), pregnancy and newly started insulin pump treatment (within three months).

Recruitment

A power analysis before recruitment suggested that at least 56 individuals with type 1 diabetes were needed to reach 80% power for detecting a clinically relevant reduction in HbA1c of 6 mmol/mol (with assumed standard deviation [SD] of 9) at a 5% significance level. To allow for dropouts of approximately 24% the aim was to recruit 70 persons with type 1 diabetes.

From 2016 to 2019 individuals with HbA1c >60 mmol/mol (reference value 42 mmol/mol) were assessed for eligibility (n=622) in five rounds. Study information and an invitation to participate were sent by post to eligible individuals who met the criteria for inclusion (n=343). Two weeks after the material was sent, a research nurse assistant telephoned the invited individuals to offer more information about the study, if desired. Eighty-one people submitted written informed consent and were included in the study. Consequently, the participation rate was 23.6% (81 of 343 who received an invitation). Sociodemographic details and information on treatment were collected at baseline.

Randomisation

For peer review only

For peer review only - http://bmjopen.bmj.com/site/about/guidelines.xhtml

After the baseline measurements, participants were randomly allocated into either the intervention or control group (Figure 1). A person not involved with the project conducted randomisation by drawing sealed lots. Stratification was carried out to achieve an equal distribution by sex in the groups. The research assistant nurse informed participants of the group assignment by e-mail and provided further instructions three weeks before the start of the intervention programme. Blinding the ACT-instructors or the research assistant nurse to the group allocations during the study period was not possible. However, the ACT-instructors never met the control group; additionally, the research nurse assistant was not involved in the intervention programme and did not have ACT skills.

Intervention

This study was developed by a multidisciplinary team consisting of psychologists, nurses specialised in diabetes and an endocrinologist**.** The ACT programme consisted of seven two-hour sessions that were held every other week. A text message was sent the day before each session as a reminder. The size of the ACT groups ranged from six to nine participants. Furthermore, the programme was manual-based and developed according to a general ACT programme (36). The manual was tailored by a psychologist specialised in diabetes and a diabetes nurse to direct the focus to living with type 1 diabetes; both were trained ACT instructors. The sessions involved activities connected to the six core components of ACT: - being present, acceptance, defusion, self as context, values, and commitment (27). Recurring activities throughout the programme were group discussions, psychoeducation, exercises, mindfulness practice, role play and value awareness. There was also homework between sessions.

The intervention group followed the ACT programme alongside TAU and the control group received TAU. TAU comprised a minimum of two visits (one appointment with an endocrinologist and one with a nurse specialised in diabetes) at the clinic each year. The needs of the patient governed the number of visits and telephone contacts.

Assessments

Time points for measurement were at baseline, weeks 8 and 14 during the intervention and repeated measurements at 6 months, one year and two years after the intervention. Web-based questionnaires were sent to participants by e-mail and reminders were sent one and two weeks after the first e-mail, if needed. The questionnaires and reminders were sent via a web-based tool. Three weeks before each follow-up assessment, a letter with information regarding the coming measurements and dates for HbA1c measurement was sent via post and via e-mail. Reminders of the upcoming dates were sent a couple of days before HbA1c measurement, which took place close to the clinic but not in the hospital area. If both the questionnaires and HbA1c measurement were completed, a gift card of 100 Swedish crowns was sent to the participant after each time point of measurement.

Primary outcome measure

The primary outcome was glycated haemoglobin, HbA1c. The measurement reflects the average glycaemic level during the previous two to three months (37). The treatment goal should be individually set; however, a general treatment target for adults is < 53 mmol/mol (38). We used capillary blood samples and equipment to conduct point-of-care immunoassay (DCA Vantage Analyzer, Siemens) which has shown sufficient correlation with the reference method, high-performance liquid chromatography (39). The method of analysis is calibrated against IFCC standard (International Federation of Clinical

For peer review only - http://bmjopen.bmj.com/site/about/guidelines.xhtml

Chemists). The same equipment was used at each measurement time point and a checklist for calibration and maintenance was followed in accordance with the manufacturer's recommendations (40).

Secondary outcome measures

Depression Anxiety Stress Scales

Depression, anxiety and stress, were assessed with The Depression Anxiety, Stress Scale (DASS21) (41). The questionnaire consists of 21 items divided into three subscales. A 4-point Likert scale, starting with *didn't apply to me at all* and ending with *applied to me very much or most of the time,* is used for rating (41). Psychometric properties have been evaluated in a Swedish population (42). Cronbach's alpha in the subscales in the current study ranged from 0.75 to 0.92.

Hypoglycaemia Fear Survey

Fear of hypoglycaemia was assessed by using The Hypoglycaemia Fear Survey (HFS) (41, 43). The scale with 23 items in total involves two subscales: worry and behaviour. A 5-point Likert Scale is used for scoring (0=never, 4=always). A higher score indicates a more explicit fear of hypoglycaemia (43). The Swedish version has been evaluated regarding psychometric properties (44). Cronbach's alpha for the total scale used in the current study was 0.86.

Problem Areas in Diabetes Scale

The Problem Areas in Diabetes Scale (PAID) is a measure for assessing emotional distress related to diabetes (45). The questionnaire consists of 20 items and is scored on a 5-point Likert scale (0=not a problem to 4= serious problem) (45). Greater distress is indicated with higher scores (total score 0-100) where 40 is the cut-off for significant distress (46, 47). The reliability and validity of the Swedish version scale have been evaluated (48). Cronbach`s alpha in the current study was 0.91.

Summary of Diabetes Self-Care Activities

The frequency of self-care activities over the past seven days was measured with the Summary of Diabetes Self-Care Activities (SDSCA) (49). The questionnaire includes eleven items in different areas of self-care activity. The scoring system ranges from 0 to 7 and refers to the number of weekdays. The areas are separately scored and are not summed in a total score. High scores indicate high self-care performance (49). A Swedish version, translated by Amsberg et al., was used which also demonstrated sufficient internal consistency for the subscales (Cronbach's alpha 0.80 – 0.86) in a similar sample (25). In the current study, Cronbach's alpha for the subscales diet, exercise and glucose monitoring ranged from 0.80 to 0.90.

Acceptance and Action Questionnaire

For peer review only - http://bmjopen.bmj.com/site/about/guidelines.xhtml

The original Acceptance and Action Questionnaire (AAQ) (50) was developed into a revised version, the AAQ-II (51) to measure the degree of psychological flexibility. The revised scale AAQ-II has been translated into Swedish and evaluated regarding psychometric features. This version was used in our study. There are six items in the Swedish version that are rated on a 7-point Likert scale (1= never true, 7 =always true). A lower score reflects greater psychological flexibility (52). Cronbach`s alpha in the current study was 0.88.

Acceptance Action Diabetes Questionnaire

Psychological flexibility related to diabetes was assessed using the Acceptance Action Diabetes Questionnaire (AADQ). The questionnaire is derived from the AAQ and was developed by Gregg et al. (53). The original AADQ comprises eleven items and is rated on a 7-point Likert scale (1= never true, 7= always true). A higher score indicates a greater psychological flexibility (53) in contrast to the AAQ which is scored in the opposite direction (52). The scale was translated into Swedish, and the psychometric properties has been evaluated. The manuscript has been accepted but is not yet published. Cronbach`s alpha in the current study was 0.77.

Manchester Short Assessment of Quality of life

The Manchester Short Assessment of Quality of life (MANSA) is used to assess quality of life (QoL). The questionnaire was developed by Priebe et al. (54) and was constructed with 16 items (12 items related to general QoL and 4 are objective items of QoL). The rating score ranges from 1 to 7 (1= could not be worse, 7 = could not be better). The response categories in the objective items are dichotomous (yes, no). A Swedish version has been evaluated regarding the psychometric properties (55). The Cronbach`s alpha in the current study was 0.85.

Statistical methods

Baseline characteristics were described as mean (SD) for continuous variables and absolute and relative frequencies for categorical variables. Tests for differences in baseline characteristics between completers and non-completers and intervention (control group, intervention group) were performed using the Kruskal-Wallis test (continuous variables) and chi-squared test (categorical variables). Mixed effect models were fitted to test for changes in HbA1c levels and questionnaire scores across follow-up. Each model included the time point (baseline, week 8, week 14, week 38, week 62) and intervention (control group, intervention group) as fixed effects and a random intercept for participants. Interaction effects between the time point and intervention were explored. Test of fixed effects were done using a likelihood ratio test in nested models using the chi-square distribution. All statistical analyses were performed using R version 3.6.2 (56). IBM SPSS 27 was used for graphs (IBM Corp., Armonk, NY, USA). Other statistical analyses that were planned and presented in the study protocol could not be conducted or were not suitable owing to a high number of missing values and lack of statistical significance in outcome. The intention-to-treat principle was applied in the primary analysis. In addition, a per-protocol analysis was conducted in a further examination of the results.

For peer review only - http://bmjopen.bmj.com/site/about/guidelines.xhtml

Fidelity check of the intervention

For quality assurance, a fidelity check was performed by videotaping one session held by the licensed psychologist and the registered nurse. The videotape was reviewed by DE, an experienced trainer who has been training instructors in the ACT-group intervention that was used. In addition, participants in two groups and the group leaders estimated the adherence to the treatment intervention and manual by completing an adherence evaluation after sessions three and seven. The aim was to measure how adherent each participant had been to the treatment interventions in the sessions and how well the group leaders adhered to the treatment manual. The group leaders also evaluated their adherence to the manual. Finally, participants' and group leaders' evaluations were compared. Participants and group leaders rated each intervention in the session on a scale from 0 to 10 where 0 = "this has not been discussed or brought up", to 10 = "This was covered thoroughly". The estimates are shown in Supplementary Table 1 and Supplementary Table 2 (Supplementary Material).

Insert figure 1

PATIENT AND PUBLIC INVOLVEMENT

The RCT was preceded by an informal pre-trial among eight volunteers with type 1 diabetes to test the design and content of the ACT intervention. On the basis of the feedback of participants, an adaptation of the programme was made with shorter but more sessions. The participants in the RCT will receive a message when the results are published and where to find them. There will also be a link provided to a webpage with a summary of the results in plain Swedish.

RESULTS

Study participants

The mean age for all study participants in the sample was 40.2 (SD 16.6) years and 70% were women. The mean duration of type 1 diabetes was 23.4 (SD 14.4) years, the mean HbA1c was 73.5 (SD 13) mmol/mol and 74.7% of participants have had retinopathy on some occasion. Approximately 88% of participants used sensor-based glucose monitoring and 35.8% wore an insulin pump. In Table 1 the baseline characteristics are described for the total sample and the allocated trial groups. No statistically significant differences between group characteristics were found at baseline. Baseline scoring of the secondary outcomes and comparisons between the groups are shown in Table 2 and Supplementary Table 3 (Supplementary Material). No statistically significant differences were found between the groups in baseline scoring (Bonferroni correction applied). Significantly elevated diabetes distress was present among 51% of the study sample, (PAID score >40). Mean scores in the normal range were noted for stress and anxiety. The mean score in the depression subscale of 12.14 (SD 10.85) indicated mild depression, and 39% of participants had scores corresponding to moderate to severe depression.

For peer review only - http://bmjopen.bmj.com/site/about/guidelines.xhtml

Table 1: Baseline characteristics of trial groups with comparisons between complete (participants completing the two-year follow-up) and drop-out (participants lost to follow-up or who discontinued the study at the two-year follow-up) groups.

**Characteristics**	**All participants**	**Intervention group**	**Control group**	**P value**	**Complete**	**Dropout**	**P value**
**N**	81	43	38		26	55	
**Males**, n (%)	30 (37)	15 (34.9)	15 (39.5)	0.844	8 (30.8)	22 (40)	0.578
**Age** (years) (SD, range)	40.2 (16.6, 19-69)	42.2 (16.5, 19-69)	37.9 (16.6, 20-68)	0.368	38.6 (16.4)	40.9 (16.8)	0.754
**Diabetes duration** (years) (SD, range)	23.4 (14.4, 3-61)	23.6 (14.0, 3-61)	23.2 (15, 5-61)	0.730	20.2 (12.5)	24.9 (15.1)	0.156
**Family/Cohabitation**							
With a partner, n (%)	36 (44.4)	17 (39.5)	19 (50)		11 (42.3)	25 (45,5)	
With parents, n (%)	14 (17.3)	7 (16.3)	7 (18.4)		3 (11.5)	11 (20.0)	
With other, n (%)	5 (6.2)	2 (4.7)	3 (7.9)		0 (0.0)	5 (9.1)	
Alone, n (%)	22 (27.2)	13 (30.2)	9 (23.7)		11 (42.3)	11 (20.0)	
Apart, n (%)	4 (4.9)	4 (9.3)	0 (0.0)	0.314	1 (3.8)	3 (5.5)	0.167
**Education (highest level)**							
Elementary school, n (%)	4 (4.9)	3 (7.0)	1 (2.6)		0 (0.0)	4 (7.3)	
Upper secondary school, n (%)	30 (37.0)	14 (32.6)	16 (42.1)		8 (30.8)	22 (40.0)	
University (courses or education) n (%)	41 (50.6)	24 (55.8)	17 (44.7)		16 (61.5)	25 (45.5)	
Other, n (%)	6 (7.4)	2 (4.7)	4 (10.5)	0.441	2 (7.7)	4 (7.3)	0.361
**Employment**							
Retired, n (%)	6 (7.4)	3 (7.0)	3 (7.9)		1 (3.8)	5 (9.1)	
Early retirement, n (%)	5 (6.2)	3 (7.0)	2 (5.3)		3 (11.5)	2 (3.6)	
Student, n (%)	11 (13.6)	5 (11.6)	6 (15.8)		3 (11.5)	8 (14.5)	
Not currently working for medical reasons, n (%)	2 (2.5)	2 (4.7)	0 (0.0)		0 (0.0)	2 (3.6)	
Unemployed, n (%)	3 (3.7)	1 (2.3)	2 (5.3)		1 (3.8)	2 (3.6)	

For peer review only - http://bmjopen.bmj.com/site/about/guidelines.xhtml

Employed, n (%)	44 (54.3)	21 (48.8)	23 (60.5)		15 (57.7)	29 (52.7)	
Manage own company, n (%)	10 (12.3)	8 (18.6)	2 (5.3)	0.420	3 (11.5)	7 (12.7)	0.730
**CGM or FGM**, n (%)	71 (87.7)	37 (86)	34 (89.5)	0.897	24 (92.3)	47 (85.5)	0.608
**CSII**, n (%)	29 (35.8)	15 (34.9)	14 (36.8)	1.000	12 (46.2)	17 (30.9)	0.277
**BMI** (SD, range)	26.1 (4.7, 17.3-39)	25.5 (4.5, 17.3-36.5)	26.8 (4.7, 19.6-39)	0.250	26.6 (3.4)	25.8 (5.1)	0.210
**Baseline HbA1c** (mmol/mol) (SD, range)	73.5 (13, 60-125)	71.4 (10.1, 60-118)	75.9 (15.4, 61-125)	0.315	69.2 (7.2)	75.5 (14.6)	0.029
**Retinopathy,** n (%) *	59 (74.7)	30 (73.2)	29 (76.3)	0.950	17 (65.4)	42 (79.2)	0.291
**Neuropathy**, n (%) *	23 (29.1)	13 (31.7)	10 (26.3)	0.780	9 (34.6)	14 (26.4)	0.624
**Nephropathy,** n (%) *							
Micro-albuminuria	4 (5.2)	2 (5.0)	2 (5.4)		2 (7.7)	2 (3.9)	
Macro-albuminuria	2 (2.6)	1 (2.5)	1 (2.7)		0 (0.0)	2 (3.9)	
No albuminuria	71 (92.2)	37 (92.5)	34 (91.9)	0.995	24 (92.3)	47 (92.2)	0.474
**Cerebrovascular disease**, n (%) *	1 (1.3)	1 (2.4)	0 (0.0)	1.000	0 (0)	1 (1.9)	1.000
**Coronary artery disease,** n (%) *	3 (3.8)	1 (2.4)	2 (5.3)	0.946	1 (3.8)	2 (3.8)	1.000

*Information missing for some individuals (nephropathy:4, neuropathy:2, retinopathy:2, cerebrovascular disease:4, coronary artery disease:2)

SD, standard deviation; BMI, body mass index; HbA1c, glycated haemoglobin; CGM, continuous glucose monitoring; FGM, flash glucose monitoring; CSII, continuous subcutaneous insulin infusion .

For peer review only - http://bmjopen.bmj.com/site/about/guidelines.xhtml

Dropouts and adherence to the programme

Eight participants did not start the intervention programme and wished to withdraw from the study. Reasons for their decision were as follows: internship in another city (n=1), going abroad (n=1), lack of time (n=1), family reasons (n=1), and having to work (n=1). In three cases, no reason was provided. Reasons for interrupting the programme were as follows: disliked the program (n=2), medical/health related reasons (n= 3), evening time not suitable (n = 1), family reasons (n= 1), and change in working schedule (n=1). One person in the intervention group passed away before the two-year follow-up. In the control group, the reasons reported for discontinuation of the study after randomisation until the last timepoint for measurement (two years) were as follows: medical reasons (n= 1), family reasons (n=1), no longer interested (n=2), lack of time (n=1) and moved to another city (n=2). Parts of the one-year and the two-year follow-up were conducted during the COVID-19 pandemic during 2020-2021. Three participants actively declined the invitation for follow-up measurement because of the pandemic (two who had symptoms and one owing to the risk of infection). However, one HbA1c measurement could be registered from the Endocrinology clinic in the same week. Most reasons for discontinuation/loss to follow-up were unknown.

Supplementary Table 4 (Supplementary Material) presents the exposure to ACT. Thirty-five participants attended at least one session (81.4%) and 20 participants (46.5%) attended four to seven sessions of the programme. The attendance for measurement of HbA1c is also shown in Supplementary Table 4. In week 8 after session 4, 62.8% of participants in the intervention group and 52.6% controls completed HbA1c measurement. The dropout rate was approximately equal in the trial arms during the follow-up period. Furthermore, no significant differences in baseline characteristics were detected among participants remaining in the study after two years and those who were lost to follow-up (Bonferroni correction applied) (Table 1).

Primary outcome

The mean HbA1c in the intervention group decreased over two years from 71.4 mmol/mol to 59.6 mmol/mol. However, the likelihood ratio test of nested models demonstrated no statistically significant interaction effect (chi-square:1, p:0.316) between measurement time point and group affiliation regarding HbA1c, as shown in Table 2. The observed mean HbA1c over time is illustrated in Figure 2.

Insert Figure 2.

For peer review only - http://bmjopen.bmj.com/site/about/guidelines.xhtml

Table 2: Observed mean value at each time point for outcome measurements and linear mixed model analysis with testing for interaction effects of time and ACT intervention on HbA1c, PAID, DASS21 and AAQ-II.

							Mixed model analysis with interaction		
	**Week 0**	**Week 8**	**Week 14**	**6 months**	**12 months**	**24 months**	**Fixed effects**	**Estimate (95% CI)**	**Interaction effect □^{2} (p value)**
**HbA1c** mmol/mol									
ACT							Intercept	75.22 (71.43, 79.01)	
N	43	27	21	15	16	15	ACT	-3.71 (-8.90, 1.49)	
Mean (SD)	71.4 (10.1)	70.1 (8.9)	69.4 (8.3)	63.6 (9.5)	64.6 (8.4)	59.6 (8.0)	Time	-0.05 (-0.09, -0.01)	
Control							ACT*time	-0.02 (-0.07, 0.03)	
N	38	20	21	15	17	11			
Mean (SD)	75.9 (15.4)	75.7 (14.4)	72.4 (11.9)	64.6 (5.7)	70.2 (13.3)	70.5 (18.6)			
P-value	0.315	0.281	0.227	0.677	0.181	0.132			0.49 (0.485)
**PAID**									
ACT							Intercept	43.52 (37.78, 49.29)	
N	39	24	21	19	17	17	ACT	-9.40 (-17.36, -1.46)	
Mean (SD)	36.6 (18.1)	32.6 (19.7)	28.7 (18.7)	31.1 (18.5)	28.6 (16.0)	27.4 (18.6)	Time	-0.05 (-0.10, -0.01)	
Control							ACT*time	-0.01 (-0.08, 0.05)	
N	32	25	25	21	19	16			
Mean (SD)	42.9 (15.9)	39.8 (20.7)	41.6 (19.2)	34.9 (20.9)	36.2 (16.6)	33.4 (17.7)			
P value	0.229	0.322	0.034	0.533	0.188	0.279			0.22(0.642)
**DASS-S**									
ACT							Intercept	16.53 (13.63, 19.44)	
N	39	24	21	19	17	17	ACT	-4.90 (-8.92, -0.89)	
Mean (SD)	12.5 (8.4)	12.5 (9.2)	9.8 (8.3)	12.8 (10.4)	9.4 (6.3)	10.7 (6.7)	Time	-0.01 (-0.03, 0.02)	
Control							ACT*time	-0.01 (-0.04, 0.03)	
N	32	25	25	21	19	16			
Mean (SD)	16.8 (11.1)	14.5 (10.7)	15.8 (9.7)	13.0 (10.9)	14.3 (11.3)	14.1 (10.7)			
P value	0.141	0.534	0.036	0.978	0.226	0.458			0.11(0.743)
**DASS-A**									
ACT							Intercept	7.54 (5.44, 9.64)	

For peer review only - http://bmjopen.bmj.com/site/about/guidelines.xhtml

N	39	24	21	19	17	17		ACT	-1.06 (-3.97, 1.85)	
Mean (SD)	6.8 (6.8)	5.8 (7.1)	5.4 (6.3)	7.3 (8.3)	4.2 (4.0)	4.6 (7.9)		Time	0.01 (-0.01, 0.03)	
Control								ACT*time	-0.02 (-0.05, 0.01)	
N	32	25	25	21	19	16				
Mean (SD)	8.2 (7.1)	6.6 (7.0)	6.9 (6.0)	6.5 (7.0)	8.0 (6.7)	7.1 (6.2)				
P value	0.302	0.460	0.283	0.763	0.042	0.063				1.97(0.161)
**DASS-D**										
ACT								Intercept	13.10 (9.87, 16.35)	
N	39	24	21	19	17	17		ACT	-2.53 (-7.03, 1.95)	
Mean (SD)	11.7 (11.4)	10.9 (11.0)	6.9 (9.4)	11.6 (12.8)	6.0 (7.4)	10.0 (7.5)		Time	0.02 (-0.01, 0.06)	
Control								ACT*time	-0.03 (-0.08, 0.01)	
N	32	25	25	21	19	16				
Mean (SD)	12.6 (10.3)	11.3 (9.0)	14.1 (10.0)	11.1 (11.7)	14.8 (11.3)	14.1 (10.5)				
P value	0.486	0.672	0.015	0.989	0.011	0.261				2.00(0.157)
**AAQ**										
ACT								Intercept	17.69 (15.34, 20.05)	
N	39	24	21	19	17	17		ACT	-1.95 (-5.20, 1.30)	
Mean (SD)	16.3 (8.4)	15.8 (8.5)	14.2 (8.3)	15.0 (9.3)	11.9 (7.8)	12.5 (7.8)		Time	0.02 (0.00, 0.04)	
Control								ACT*time	-0.05 (-0.08, -0.02)	
N	32	25	25	21	19	16				
Mean (SD)	18.1 (6.1)	16.0 (7.0)	17.4 (7.3)	16.7 (7.2)	17.9 (7.9)	18.9 (7.8)				
P value	0.325	0.749	0.095	0.356	0.016	0.013				12.63 (<0.001)

*DASS-S-stress, DASS-A-anxiety, DASS-D-depression

ACT, acceptance and commitment therapy; SD, standard deviation; CI, confidence interval; HbA1c, glycated haemoglobin; PAID, Problem Areas in Diabetes Scale; DASS, Depression, Anxiety, Stress Scale; AAQ, Acceptance and Action Questionnaire.

For peer review only

For peer review only - http://bmjopen.bmj.com/site/about/guidelines.xhtml

Secondary outcomes

A significant interaction effect (likelihood ratio test chi-square:12.63, $p<0.001$) between time and group allocation was found in AAQ-II scores measuring psychological flexibility (Table 2). This finding indicates a trend of improvement in the ACT group over time. The observed mean AAQ-II score over two years is illustrated in Figure 3.

No significant interaction effects between time and group allocation were demonstrated in scores of the PAID, DASS21 (Table 2), HFS, AADQ, MANSA or SDSCA (Supplementary Table 3, Supplementary Material).

Insert Figure 3

Per protocol

Per protocol analyses were conducted; the results were consistent with findings in the intention-to-treat analyses.

DISCUSSION

This study evaluated the impact of an ACT programme tailored for individuals living with type 1 diabetes. The observed mean values decreased to a level below 60 mmol/mol in the intervention group during the study period. However, no statistically significant effect on HbA1c was demonstrated two years after the ACT program. The AAQ-II scores decreased over time and the improvement in psychological flexibility was statistically significant after one and two years. The results regarding HbA1c correspond to those of a meta-analysis involving overall psychological interventions in type 1 diabetes (57) and a meta-analysis of CBT interventions for diabetes related distress in type 1 and type 2 diabetes (58). Some CBT intervention studies solely focusing on type 1 diabetes have shown similar findings (59, 60). At the same time, the meta-analysis of Winkley et. al (2020) and Yang et al (2020) concluded that CBT has the potential to be effective for HbA1c reduction in type 1 diabetes (57, 61) which was clearly demonstrated in the study of Amsberg et .al (2008). Regarding ACT intervention in diabetes the findings in our study stand in contrast to those of previous studies. ACT interventions in type 2 diabetes have shown promising results (33). A recent study of an ACT intervention for adolescents with type 1 diabetes showed a significant beneficial effect on HbA1c (62). To our knowledge, this is one of the first RCTs to evaluate the effect of a group-based face-to-face ACT intervention on HbA1c in an adult population. We used a longitudinal design with a long-term follow-up period of two years which can enlighten the mechanism of change over time. The study protocol was developed according to Standard Protocol Items: Recommendations for Interventional Trials (SPIRIT) 2013 Statement (63) and was previously published and open for transparency and replication. We used instruments that have been evaluated for psychometric properties (with one exception, the translated version of the SDSCA), and the same device for HbA1c measurements.

Interestingly, a persistent beneficial effect was found in psychological flexibility during the follow-up period. The effect was seen one year after the intervention but not before that time. This finding may indicate that psychological flexibility takes time to develop; this was also discussed in a previous study

For peer review only - http://bmjopen.bmj.com/site/about/guidelines.xhtml

(64). We were unable to conduct mediation analysis regarding the role of psychological flexibility in self-management and HbA1c improvement owing to the absence of statistical significance in the outcome variables. However, a development process that requires time would hypothetically generate improvement in HbA1c even later in the follow-up period. Notably, the statistically significant change in psychological flexibility was only observed in the generic AAQ-II and not in the disease-specific AADQ. The AAQ-II is the most frequently used instrument in research for measuring psychological flexibility, but it has also been criticised for being a measure for psychological distress rather than of psychological flexibility (65).

Reflections have been made regarding the connection between the content of the psychological intervention and diabetes self-management in explaining outcomes in similar studies (57, 62). Gregg et al. (2007) evaluated the impact of an educational intervention with and without ACT components in type 2 diabetes (53). The current study did not focus on self-management and did not involve elements of diabetes education, which may have been a disadvantage. Another observed aspect that differentiates the current study from other group-based studies showing HbA1c improvement is the individualised perspective. The intervention in the study by Amsberg et al. (2008) included individual meetings (25), and Alho et al. (2022) conducted interviews regarding expectations prior to the intervention (62). Moreover, participants called for more individualisation in a feasibility trial of a web-based ACT programme (34). An example of a beneficial individualised element in psychological intervention trials is a study by Malin et al. (2020). A brief motivational interview was introduced after the initial session which increased adherence and decreased the drop-out rate in a CBT intervention of chronic pain and cancer (66).

This study has several limitations, among which the main limitation is the dropout rate. Eight individuals (18.6%) allocated to the ACT group did not start the intervention which is in line with a meta-analysis of dropout rates in CBT trials (67). However, the dropout rate during the ACT programme period in the current study far exceeded the aggregated dropout rate in the study by Fernandez et. al (2015) (67). In the study by Bendig et al. (2021) (34) that involved a web-based ACT programme, the dropout rate was 57% in the intervention group during the study period which is more similar to our findings. Nevertheless, there are no indications of different dropout rates between trials based on ACT or other psychological interventions (68). In addition, the Covid-19 pandemic may have affected the attendance for measurement of HbA1c during parts of the one-year and two-years follow-up periods. There were no differences in baseline characteristics between participants who remained at the two-year follow-up and those who were lost to follow-up at the same measurement time point. Without a Bonferroni correction, the HbA1c demonstrated higher values in the group lost to follow-up which would imply a selection bias. Furthermore, the consequence of the high attrition is an underpowered analysis, which may entail an elevated risk of type II error. Another deficiency in the study is the lack of a comprehensive process evaluation such as that Craig et al. (2008) described in the Medical Research Council guidelines for complex interventions (69). On the other hand, we have gained valuable knowledge regarding the context and implementation of ACT groups for adults with type 1 diabetes that lays a foundation for further development.

Considering that this was a single-center study with significant attrition problems, the external validity is weakened. The study is ongoing with the last measurement timepoint at five years after the intervention. The decision to proceed with follow-up measurements despite the high attrition was made owing to the effects of psychological flexibility and tendencies toward a decreasing HbA1c.

For peer review only - http://bmjopen.bmj.com/site/about/guidelines.xhtml

In conclusion, this study showed no statistically significant difference between the groups regarding the primary outcome measure, HbA1c. However, the ACT programme showed a persistent beneficial impact on psychological flexibility in the intervention group.

Author contributions

Study conceptualisation and design: TA, SA, FL, ET. Acquisition of data: IW, ET. Analysis and interpretation of data: IW in collaboration with an external statistician and TA, U-BJ, SA. Manuscript draft: IW. Revision and approval of final draft: TA, U-BJ, SA, ET, FL.

Ethics approval

This study was approved by the Swedish Ethical Review Authority (2016/14-31/1).

Funding

The study was supported by Lindhés advokatbyrå AB (grant nr: LA2016-0429), the Annie and Fritz Tjus donation fund (grant nr: N/A), the Sophiahemmet Foundation (grant nr: N/A) and the Swedish Diabetes Foundation (grant nr: DIA2018-360).

Competing interests

FL receives royalties from his books written on ACT and income from training professionals in ACT.

Data sharing statement

The data may be available upon reasonable request.

Acknowledgement

We would like to thank all participants in the study and RN Neide Johansson for supporting the recruitment and data collection. Furthermore, we would like to acknowledge Fredrik Wiklund (Statisticon AB) for statistical support and Psychologist Daniel Ek (DE) for the fidelity check of the videotaped material. We also thank Edanz for language editing.

For peer review only

REFERENCES

1. WHO. Classification of Diabetes Mellitus. Geneva: WHO; 2019.

2. Patterson CC, Karuranga S, Salpea P, Saeedi P, Dahlquist G, Soltesz G, et al. Worldwide estimates of incidence, prevalence and mortality of type 1 diabetes in children and adolescents: Results from the International Diabetes Federation Diabetes Atlas, 9th edition. Diabetes Res Clin Pract. 2019;157:107842. https://doi.org/10.1016/j.diabres.2019.107842

3. DiMeglio LA, Evans-Molina C, Oram RA. Type 1 diabetes. Lancet. 2018;391(10138):2449-62. https://doi.org/10.1016/S0140-6736(18)31320-5

4. American Diabetes Association. 6. Glycemic Targets: Standards of Medical Care in Diabetes—2022. Diabetes Care. 2021;45(Supplement_1):S83-S96. https://doi.org/10.2337/dc22-S006

5. The National Board of Health and Welfare. Nationella riktlinjer för diabetesvård - Stöd för styrning och ledning. Report No.: 978-91-75555-476-1. Socialstyrelsen 2018 www.socialstyrelsen.se

6. The Swedish National Diabetes Register. Nationella Diabetesregistrets årsrapport 2021. www.ndr.nu
7. Shah VN, Grimsmann JM, Foster NC, Dost A, Miller KM, Pavel M, et al. Undertreatment of cardiovascular risk factors in the type 1 diabetes exchange clinic network (United States) and the prospective diabetes follow-up (Germany/Austria) registries. Diabetes Obes Metab. 2020;22(9):1577-85. https://doi.org/10.1111/dom.14069
8. Renard E, Ikegami H, Daher Vianna AG, Pozzilli P, Brette S, Bosnyak Z, et al. The SAGE study: Global observational analysis of glycaemic control, hypoglycaemia and diabetes management in T1DM. Diabetes Metab Res Rev. 2021;37(7):e3430. https://doi.org/10.1002/dmrr.3430
9. Funnell MM, Bootle S, Stuckey HL. The diabetes attitudes, wishes and needs second study. Clin Diabetes. 2015;33(1):32-6. https://doi.org/10.2337/diaclin.33.1.32
10. Reddy M, Rilstone S, Cooper P, Oliver NS. Type 1 diabetes in adults: supporting self management. BMJ. 2016;352:i998. https://doi.org/10.1136/bmj.i998
11. Iqbal A, Novodvorsky P, Heller SR. Recent Updates on Type 1 Diabetes Mellitus Management for Clinicians. Diabetes Metab J. 2018;42(1):3-18. https://doi.org/10.4093/dmj.2018.42.1.3
12. Jull J, Witteman HO, Ferne J, Yoganathan M, Stacey D. Adult-Onset Type 1 Diabetes: A Qualitative Study of Decision-Making Needs. Can J Diabetes. 2016;40(2):164-9. https://doi.org/10.1016/j.jcjd.2015.09.080
13. Fisher L, Hessler D, Polonsky W, Strycker L, Masharani U, Peters A. Diabetes distress in adults with type 1 diabetes: Prevalence, incidence and change over time. J Diabetes Complications. 2016;30(6):1123-8. https://doi.org/10.1016/j.jdiacomp.2016.03.032
14. Sturt J, Dennick K, Due-Christensen M, McCarthy K. The detection and management of diabetes distress in people with type 1 diabetes. Curr Diab Rep. 2015;15(11):101. https://doi.org/10.1007/s11892-015-0660-z
15. Roy T, Lloyd CE. Epidemiology of depression and diabetes: a systematic review. J Affect Disord. 2012;142 Suppl:S8-21. https://doi.org/10.1016/S0165-0327(12)70004-6
16. van Dooren FE, Nefs G, Schram MT, Verhey FR, Denollet J, Pouwer F. Depression and risk of mortality in people with diabetes mellitus: a systematic review and meta-analysis. PloS one. 2013;8(3):e57058. https://doi.org/10.1371/journal.pone.0057058
17. de Groot M, Golden SH, Wagner J. Psychological conditions in adults with diabetes. Am Psychol. 2016;71(7):552-62. https://doi.org/10.1037/a0040408
18. Hessler DM, Fisher L, Polonsky WH, Masharani U, Strycker LA, Peters AL, et al. Diabetes distress is linked with worsening diabetes management over time in adults with Type 1 diabetes. Diabet Med. 2017;34(9):1228-34. https://doi.org/10.1111/dme.13381
19. Melin EO, Thunander M, Svensson R, Landin-Olsson M, Thulesius HO. Depression, obesity, and smoking were independently associated with inadequate glycemic control in patients with type 1 diabetes. Eur J Endocrinol. 2013;168(6):861-9. https://doi.org/10.1530/EJE-13-0137
20. Jones A, Vallis M, Pouwer F. If it does not significantly change HbA1c levels why should we waste time on it? A plea for the prioritization of psychological well-being in people with diabetes. Diabet Med. 2015;32(2):155-63. https://doi.org/10.1111/dme.12620
21. Holt RIG, DeVries JH, Hess-Fischl A, Hirsch IB, Kirkman MS, Klupa T, et al. The management of type 1 diabetes in adults. A consensus report by the American Diabetes Association (ADA) and the European Association for the Study of Diabetes (EASD). Diabetologia. 2021;64(12):2609-52. https://doi.org/10.1007/s00125-021-05568-3
22. Fenn K, Byrne, M. . The key principals of cognitive behaviour therapy. InnovAiT: Education and inspiration for general practice. 2013;6(9). https://doi.org/10.1177/1755738012471029

For peer review only - http://bmjopen.bmj.com/site/about/guidelines.xhtml

23. Lepping P, Whittington R, Sambhi RS, Lane S, Poole R, Leucht S, et al. Clinical relevance of findings in trials of CBT for depression. Eur Psychiatry 2017;45:207-11. https://doi.org/10.1016/j.eurpsy.2017.07.003
24. Uchendu C, Blake H. Effectiveness of cognitive-behavioural therapy on glycaemic control and psychological outcomes in adults with diabetes mellitus: a systematic review and meta-analysis of randomized controlled trials. Diabet Med. 2017;34(3):328-39. https://doi.org/10.1111/dme.13195
25. Amsberg S, Anderbro T, Wredling R, Lisspers J, Lins PE, Adamson U, et al. A cognitive behavior therapy-based intervention among poorly controlled adult type 1 diabetes patients--a randomized controlled trial. Patient Educ Couns. 2009;77(1):72-80. https://doi.org/10.1016/j.pec.2009.01.015
26. Hayes S, Strosahl K, Wilson K. Acceptance and Commitment Therapy: The Process and Practice of Mindful Change. New York: Guilford Press 2011.
27. Hayes SC, Levin ME, Plumb-Vilardaga J, Villatte JL, Pistorello J. Acceptance and Commitment Therapy and Contextual Behavioral Science: Examining the Progress of a Distinctive Model of Behavioral and Cognitive Therapy. Behav Ther. 2013;44(2):180-98. https://doi.org/10.1016/j.beth.2009.08.002
28. Ruiz F. A review of Acceptance and Commitment Therapy (ACT) empirical evidence: Correlational, experimental psychopathology, component and outcome studies. International Journal of Psychology and Psychological Therapy. 2010;10:125-62.
29. Kashdan TB, Rottenberg J. Psychological flexibility as a fundamental aspect of health. Clin Psychol Rev. 2010;30(7):865-78. https://doi.org/10.1016/j.cpr.2010.03.001
30. A-Tjack J, Davis ML, Morina N, Powers MB, Smits JA, Emmelkamp PM. A meta-analysis of the efficacy of acceptance and commitment therapy for clinically relevant mental and physical health problems. Psychother Psychosom. 2015;84(1):30-6. https://doi.org/10.1159/000365764
31. Graham CD, Gouick J, Krahé C, Gillanders D. A systematic review of the use of Acceptance and Commitment Therapy (ACT) in chronic disease and long-term conditions. Clin Psychol Rev. 2016;46:46-58. https://doi.org/10.1016/j.cpr.2016.04.009
32. Nicholas JA, Yeap BB, Cross D, Burkhardt MS. Psychological flexibility is associated with less diabetes distress and lower glycated haemoglobin in adults with type 1 diabetes. Intern Med J. 2022;52(6):952-8. https://doi.org/10.1111/imj.15250
33. Sakamoto R, Ohtake Y, Kataoka Y, Matsuda Y, Hata T, Otonari J, et al. The efficacy of acceptance and commitment therapy for people with type 2 diabetes: Systematic review and meta-analysis. J Diabetes Investig. 2021. https://doi.org/10.1111/jdi.13658
34. Bendig E, Bauereiss N, Schmitt A, Albus P, Baumeister H. ACTonDiabetes-a guided psychological internet intervention based on Acceptance and Commitment Therapy (ACT) for adults living with type 1 or 2 diabetes: results of a randomised controlled feasibility trial. BMJ open. 2021;11(7):e049238-e. https://doi.org/10.1136/bmjopen-2021-049238
35. Amsberg S, Wijk I, Livheim F, Toft E, Johansson UB, Anderbro T. Acceptance and commitment therapy (ACT) for adult type 1 diabetes management: study protocol for a randomised controlled trial. BMJ Open. 2018;8(11):e022234. https://doi.org/10.1136/bmjopen-2018-022234
36. Livheim F. ACT treatment for youth – a contextual behavioral approach. [dissertation] Stockholm: Karolinska Institutet 2019. https://openarchive.ki.se/xmlui/bitstream/handle/10616/46394/Thesis_Fredrik_Livheim.pdf?sequence=3&isAllowed=y
37. Jeffcoate SL. Diabetes control and complications: the role of glycated haemoglobin, 25 years on. Diabet Med. 2004;21(7):657-65. https://doi.org/10.1046/j.1464-5491.2003.01065.x
38. American Diabetes Association. 6. Glycemic Targets: Standards of Medical Care in Diabetes—2021. Diabetes Care. 2020;44(Supplement_1):S73-S84. https://doi.org/10.2337/dc21-S006

For peer review only - http://bmjopen.bmj.com/site/about/guidelines.xhtml

39. Guadalupe Vargas M, Pazmiño Gomez BJ, Vera Lorenti FE, Álvarez Condo GM, Rodas Neira EI, Veron D, et al. Assessment of two glycated hemoglobin immunoassays. Endocrinol Diabetes Nutr (English ed). 2020;67(5):297-303. https://doi.org/10.1016/j.endinu.2019.09.011
40. Healthineers S. DCA Vantage Analyzer: Siemens Healthineers; [cited 2022 24-10]. Available from: https://www.siemens-healthineers.com/se/diabetes/diabetes/dca-vantage-analyzer
41. Lovibond PF, Lovibond SH. The structure of negative emotional states: comparison of the Depression Anxiety Stress Scales (DASS) with the Beck Depression and Anxiety Inventories. Behav Res Ther. 1995;33(3):335-43. https://doi.org/10.1016/0005-7967(94)00075-u
42. Alfonsson S, Wallin E, Maathz P. Factor structure and validity of the Depression, Anxiety and Stress Scale-21 in Swedish translation. J Psychiatr Ment Health Nurs. 2017;24(2-3):154-62. https://doi.org/10.1111/jpm.12363
43. Cox DJ, Irvine A, Gonder-Frederick L, Nowacek G, Butterfield J. Fear of hypoglycemia: quantification, validation, and utilization. Diabetes Care. 1987;10(5):617-21. https://doi.org/10.2337/diacare.10.5.617
44. Anderbro T, Amsberg S, Wredling R, Lins PE, Adamson U, Lisspers J, et al. Psychometric evaluation of the Swedish version of the Hypoglycaemia Fear Survey. Patient Educ Couns. 2008;73(1):127-31. https://doi.org/10.1016/j.pec.2008.03.022
45. Polonsky WH, Anderson BJ, Lohrer PA, Welch G, Jacobson AM, Aponte JE, et al. Assessment of diabetes-related distress. Diabetes Care. 1995;18(6):754-60. https://doi.org/10.2337/diacare.18.6.754
46. Hermanns N, Kulzer B, Krichbaum M, Kubiak T, Haak T. How to screen for depression and emotional problems in patients with diabetes: comparison of screening characteristics of depression questionnaires, measurement of diabetes-specific emotional problems and standard clinical assessment. Diabetologia. 2006;49(3):469-77. https://doi.org/10.1007/s00125-005-0094-2
47. Welch GW, Jacobson AM, Polonsky WH. The Problem Areas in Diabetes Scale. An evaluation of its clinical utility. Diabetes Care. 1997;20(5):760-6. https://doi.org/10.2337/diacare.20.5.760
48. Amsberg S, Wredling R, Lins PE, Adamson U, Johansson UB. The psychometric properties of the Swedish version of the Problem Areas in Diabetes Scale (Swe-PAID-20): scale development. Int J Nurs Stud. 2008;45(9):1319-28. https://doi.org/10.1016/j.ijnurstu.2007.09.010
49. Toobert DJ, Hampson SE, Glasgow RE. The summary of diabetes self-care activities measure: results from 7 studies and a revised scale. Diabetes Care. 2000;23(7):943-50. https://doi.org/10.2337/diacare.23.7.943
50. Hayes S, Strosahl K, Wilson K, Bissett RT, Pistorello J, Toarmino D, et al. Measuring experiential avoidance: A preliminary test of a working model. The Psychological record. 2004;54:553-78. https://doi.org/10.1007/BF03395492
51. Bond FW, Hayes SC, Baer RA, Carpenter KM, Guenole N, Orcutt HK, et al. Preliminary psychometric properties of the Acceptance and Action Questionnaire-II: a revised measure of psychological inflexibility and experiential avoidance. Behav Ther. 2011;42(4):676-88. https://doi.org/10.1016/j.beth.2011.03.007
52. Lundgren T, Parling T. Swedish Acceptance and Action Questionnaire (SAAQ): a psychometric evaluation. Cogn Behav Ther. 2017;46(4):315-26. https://doi.org/10.1080/16506073.2016.1250228
53. Gregg JA, Callaghan GM, Hayes SC, Glenn-Lawson JL. Improving diabetes self-management through acceptance, mindfulness, and values: a randomized controlled trial. J Consult Clin Psychol. 2007;75(2):336-43. https://doi.org/10.1037/0022-006X.75.2.336
54. Priebe S, Huxley P, Knight S, Evans S. Application and results of the Manchester Short Assessment of Quality of Life (MANSA). Int J Soc psychiatry. 1999;45(1):7-12. https://doi.org/10.1177/002076409904500102

For peer review only - http://bmjopen.bmj.com/site/about/guidelines.xhtml

55. Björkman T, Svensson B. Quality of life in people with severe mental illness. Reliability and validity of the Manchester Short Assessment of Quality of Life (MANSA). Nord J Psychiatry. 2005;59(4):302-6. https://doi.org/10.1080/08039480500213733
56. R Core Team. R: A language and environment for statistical computing. Vienna, Austria.: R Foundation for Statistical Computing; 2019 [Available from: https://www.R-project.org.]
57. Winkley K, Upsher R, Stahl D, Pollard D, Brennan A, Heller S, et al. Systematic review and meta-analysis of randomized controlled trials of psychological interventions to improve glycaemic control in children and adults with type 1 diabetes. Diabet Med. 2020;37(5):735-46. https://doi.org/10.1111/dme.14264
58. Jenkinson E, Knoop I, Hudson JL, Moss-Morris R, Hackett RA. The effectiveness of cognitive behavioural therapy and third-wave cognitive behavioural interventions on diabetes-related distress: A systematic review and meta-analysis. Diabet Med. 2022;39(11):e14948. https://doi.org/10.1111/dme.14948
59. Snoek FJ, van der Ven NC, Twisk JW, Hogenelst MH, Tromp-Wever AM, van der Ploeg HM, et al. Cognitive behavioural therapy (CBT) compared with blood glucose awareness training (BGAT) in poorly controlled Type 1 diabetic patients: long-term effects on HbA moderated by depression. A randomized controlled trial. Diabet Med. 2008;25(11):1337-42. https://doi.org/10.1111/j.1464-5491.2008.02595.x
60. van der Ven NC, Hogenelst MH, Tromp-Wever AM, Twisk JW, van der Ploeg HM, Heine RJ, et al. Short-term effects of cognitive behavioural group training (CBGT) in adult Type 1 diabetes patients in prolonged poor glycaemic control. A randomized controlled trial. Diabet Med. 2005;22(11):1619-23. https://doi.org/10.1111/j.1464-5491.2005.01691.x
61. Yang X, Li Z, Sun J. Effects of Cognitive Behavioral Therapy–Based Intervention on Improving Glycaemic, Psychological, and Physiological Outcomes in Adult Patients With Diabetes Mellitus: A Meta-Analysis of Randomized Controlled Trials. Front Psychiatry. 2020;11(711). https://doi.org/10.3389/fpsyt.2020.00711
62. Alho I, Lappalainen P, Muotka J, Lappalainen R. Acceptance and commitment therapy group intervention for adolescents with type 1 diabetes: A randomized controlled trial. J Contextual Behav Sci. 2022;25:153-61. https://doi.org/10.1016/j.jcbs.2022.08.002
63. Chan AW, Tetzlaff JM, Altman DG, Laupacis A, Gøtzsche PC, Krleža-Jerić K, et al. SPIRIT 2013 statement: defining standard protocol items for clinical trials. Ann Intern Med. 2013;158(3):200-7. https://doi.org/10.7326/0003-4819-158-3-201302050-00583
64. Fledderus M, Bohlmeijer ET, Smit F, Westerhof GJ. Mental health promotion as a new goal in public mental health care: a randomized controlled trial of an intervention enhancing psychological flexibility. Am J Public Health. 2010;100(12):2372. https://doi.org/10.2105/AJPH.2010.196196
65. Cherry KM, Hoeven EV, Patterson TS, Lumley MN. Defining and measuring "psychological flexibility": A narrative scoping review of diverse flexibility and rigidity constructs and perspectives. Clin Psychol Rev. 2021;84:101973. https://doi.org/10.1016/j.cpr.2021.101973
66. Malins S, Biswas S, Rathbone J, Vogt W, Pye N, Levene J, et al. Reducing dropout in acceptance and commitment therapy, mindfulness-based cognitive therapy, and problem-solving therapy for chronic pain and cancer patients using motivational interviewing. Br J Clin Psychol. 2020;59(3):424-38. https://doi.org/10.1111/bjc.12254
67. Fernandez E, Salem D, Swift J, Ramtahal N. Meta-Analysis of Dropout From Cognitive Behavioral Therapy: Magnitude, Timing, and Moderators. J Consult Clin Psychol. 2015;83. https://doi.org/10.1037/ccp0000044
68. Ong CW, Lee EB, Twohig MP. A meta-analysis of dropout rates in acceptance and commitment therapy. Behav Res Ther. 2018;104:14-33. https://doi.org/10.1016/j.brat.2018.02.004

For peer review only - http://bmjopen.bmj.com/site/about/guidelines.xhtml

69. Craig P, Dieppe P, Macintyre S, Michie S, Nazareth I, Petticrew M. Developing and evaluating complex interventions: the new Medical Research Council guidance. BMJ. 2008;337:a1655. https://doi.org/10.1136/bmj.a1655

Figure 1: Flow diagram of recruitment, randomisation and attrition

HbA1c, glycated haemoglobin.

Figure 2: Mean values of HbA1c over two years by group affiliation

ACT, acceptance and commitment therapy; HbA1c, glycated haemoglobin; CI, confidence interval.

Figure 3: Mean values of AAQ-II score over two years by group affiliation

AAQ, Acceptance and Action Questionnaire; HbA1c, glycated haemoglobin; CI, confidence interval.

For peer review only

For peer review only - http://bmjopen.bmj.com/site/about/guidelines.xhtml

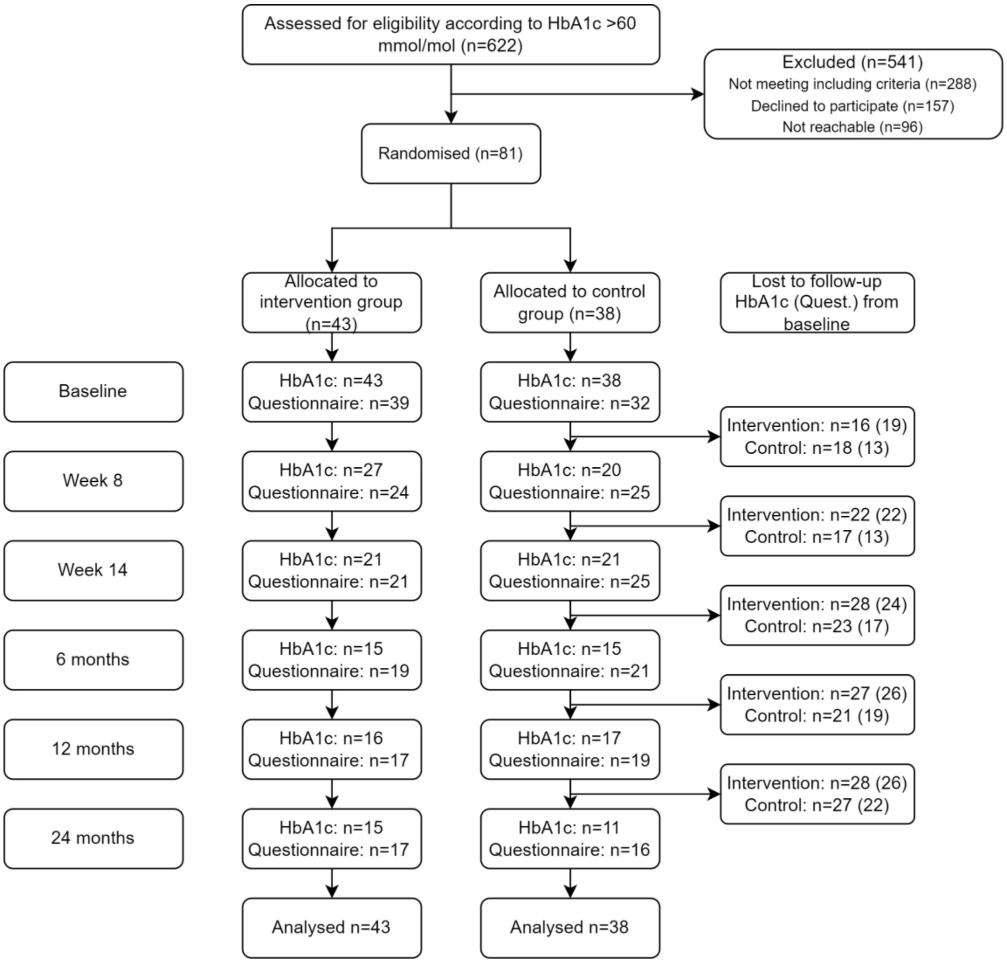

Figure 1 Flow diagram of recruitment, randomisation and attrition
HbA1c, glycated haemoglobin.

210x200mm (300 x 300 DPI)

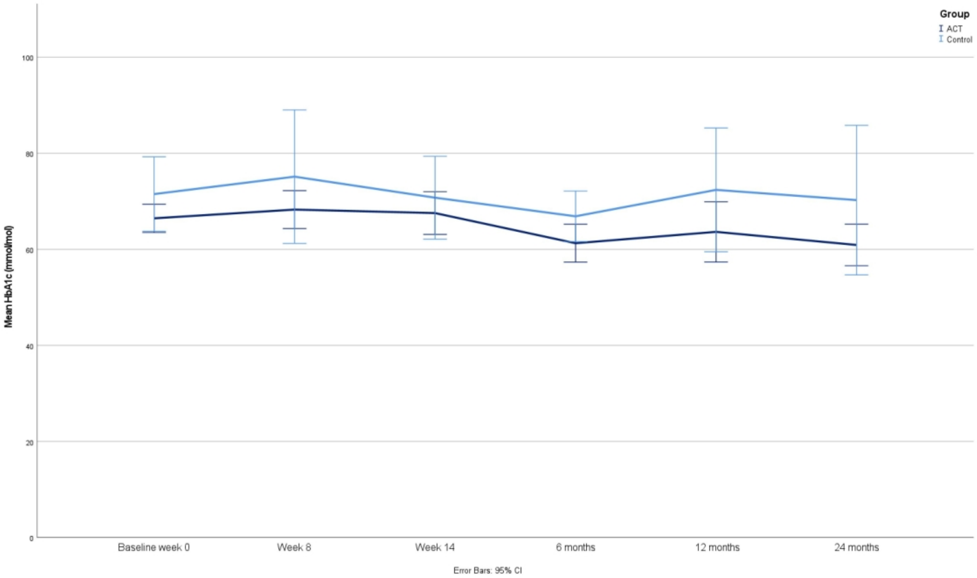

Figure 2 Mean values of HbA1c over two years by group affiliation
ACT, acceptance and commitment therapy; HbA1c, glycated haemoglobin; CI, confidence interval.

271x159mm (300 x 300 DPI)

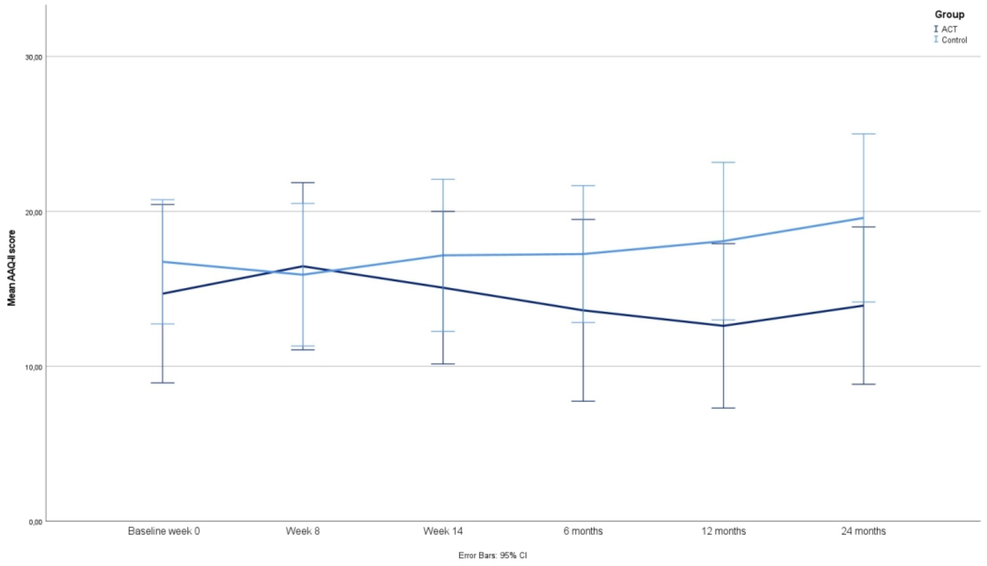

Figure 3: Mean values of AAQ-II score over two years by group affiliation
AAQ, Acceptance and Action Questionnaire; HbA1c, glycated haemoglobin; CI, confidence interval.

271x159mm (300 x 300 DPI)

CONSORT 2010 checklist of information to include when reporting a randomised trial*

Section/Topic	Item No	Checklist item	Reported on page No
**Title and abstract**			
	1a	Identification as a randomised trial in the title	Title page
	1b	Structured summary of trial design, methods, results, and conclusions (for specific guidance see CONSORT for abstracts)	1
**Introduction**			
Background and objectives	2a	Scientific background and explanation of rationale	2
	2b	Specific objectives or hypotheses	3
**Methods**			
Trial design	3a	Description of trial design (such as parallel, factorial) including allocation ratio	3-4
	3b	Important changes to methods after trial commencement (such as eligibility criteria), with reasons	-
Participants	4a	Eligibility criteria for participants	3
	4b	Settings and locations where the data were collected	4
Interventions	5	The interventions for each group with sufficient details to allow replication, including how and when they were actually administered	4
Outcomes	6a	Completely defined pre-specified primary and secondary outcome measures, including how and when they were assessed	4-5
	6b	Any changes to trial outcomes after the trial commenced, with reasons	-
Sample size	7a	How sample size was determined	3
	7b	When applicable, explanation of any interim analyses and stopping guidelines	-
Randomisation:			
Sequence generation	8a	Method used to generate the random allocation sequence	3
	8b	Type of randomisation; details of any restriction (such as blocking and block size)	3
Allocation concealment mechanism	9	Mechanism used to implement the random allocation sequence (such as sequentially numbered containers), describing any steps taken to conceal the sequence until interventions were assigned	3-4
Implementation	10	Who generated the random allocation sequence, who enrolled participants, and who assigned participants to interventions	3-4
Blinding	11a	If done, who was blinded after assignment to interventions (for example, participants, care providers, those	-

For peer review only

		assessing outcomes) and how	
	11b	If relevant, description of the similarity of interventions	-
Statistical methods	12a	Statistical methods used to compare groups for primary and secondary outcomes	6
	12b	Methods for additional analyses, such as subgroup analyses and adjusted analyses	-
**Results**			
Participant flow (a diagram is strongly recommended)	13a	For each group, the numbers of participants who were randomly assigned, received intended treatment, and were analysed for the primary outcome	7
	13b	For each group, losses and exclusions after randomisation, together with reasons	7&10
Recruitment	14a	Dates defining the periods of recruitment and follow-up	3-4
	14b	Why the trial ended or was stopped	-
Baseline data	15	A table showing baseline demographic and clinical characteristics for each group	8-9
Numbers analysed	16	For each group, number of participants (denominator) included in each analysis and whether the analysis was by original assigned groups	7
Outcomes and estimation	17a	For each primary and secondary outcome, results for each group, and the estimated effect size and its precision (such as 95% confidence interval)	10-13
	17b	For binary outcomes, presentation of both absolute and relative effect sizes is recommended	-
Ancillary analyses	18	Results of any other analyses performed, including subgroup analyses and adjusted analyses, distinguishing pre-specified from exploratory	13
Harms	19	All important harms or unintended effects in each group (for specific guidance see CONSORT for harms)	-
**Discussion**			
Limitations	20	Trial limitations, addressing sources of potential bias, imprecision, and, if relevant, multiplicity of analyses	14
Generalisability	21	Generalisability (external validity, applicability) of the trial findings	14
Interpretation	22	Interpretation consistent with results, balancing benefits and harms, and considering other relevant evidence	13-14
**Other information**			
Registration	23	Registration number and name of trial registry	1
Protocol	24	Where the full trial protocol can be accessed, if available	3
Funding	25	Sources of funding and other support (such as supply of drugs), role of funders	15

*We strongly recommend reading this statement in conjunction with the CONSORT 2010 Explanation and Elaboration for important clarifications on all the items. If relevant, we also recommend reading CONSORT extensions for cluster randomised trials, non-inferiority and equivalence trials, non-pharmacological treatments, herbal interventions, and pragmatic trials. Additional extensions are forthcoming: for those and for up to date references relevant to this checklist, see www.consort-statement.org.

For peer review only - http://bmjopen.bmj.com/site/about/guidelines.xhtml
For peer review only

Supplementary Table 1: Estimates of the adherence among the instructors (n=2) to the ACT programme.

	Group A (n=5) s 3 median (range)	Instructors s 3 median (range)	Group A (n=5) s 7 median (range)	Instructors s 7 median (range)	Group B (n=3) s 3 median (range)	Instructors s 3 median (range)
Review of preceding sessions	8 (6-9)	10 (10-10)	9 (9-10)	8 (8-8)	7 (6-9)	9 (9-9)
Feedback on homework	7 (5-10)	7.5 (7-8)	9 (8-10)	6.5 (6-7)	8 (7-10)	8 (7-9)
The Compass of life	10 (7-10)	7.5 (7-8)	9 (9-10)	8 (8-8)	9 (7-10)	9.5 (9-10)
Specific exercise	9.5 (8-10)	8.5 (8-9)	10 (8-10)	9 (9-9)	9 (6-10)	9.5 (9-10)

Estimations on a scale 1-10 (0= "this has not been discussed/brought up", to 10 = "This was covered thoroughly".)
Examples of items from the adherence evaluation. s 3: session 3, s 7: session 7

Supplementary Table 2: Estimates of the adherence among participants in two groups to the ACT programme.

	Group A (n=5) s 3 median(range)	Group A (n=5) s 7 median(range)	Group B (n=3) s 3 median(range)
Made changes in life[1]	2 (1-2)	2.5 (2-3)	2 (2-3)
App use (mindfulness)[2]	0 (0-2)	1 (0-1)	2 (0-10)
Mindfulness daily life[3]	3 (2-4)	3.5 (2-4)	4 (3-4)

1. Have you since the course started, decided to change something/take steps in your life direction? 1=No, 2=I have decided and made a small step/change,3=I have decided and made several changes/steps.

2. How many times per week did you practise mindfulness with the app "I´m here now"?

3. How often do you practise mindfulness in your daily life? 1=Never,2=Seldom, 3=Once a week/sometimes,4=Once per day, 5=Several times a day

s 3: session 3, s 7: session 7

For peer review only

Supplementary Table 3: Observed mean value at each time point for outcome measurements and linear mixed model analysis with testing for interaction effects of time and ACT intervention on HFS, AADQ, MANSA and SDSCA

**HFS-B**										
ACT								Intercept	20.27 (18.61, 21.93)	
N	39	24	21	19	17	17		ACT	-3.14 (-5.44, -0.85)	
Mean (SD)	18.1 (4.9)	15.8 (5.2)	15.7 (4.6)	15.9 (4.4)	15.4 (4.5)	16.7 (6.8)		Time	-0.02 (-0.03, 0.00)	
Control								ACT*time	0.01 (-0.01, 0.03)	
N	32	25	25	21	19	16				
Mean (SD)	20.4 (5.3)	19.5 (4.9)	19.5 (6.7)	18.7 (6.0)	18.7 (5.8)	19.6 (5.3)				
P-value	0.030	0.013	0.021	0.162	0.177	0.139				0.55 (0.457)
**HFS-W**										
ACT								Intercept	17.00 (14.02, 19.99)	
N	39	24	21	19	17	17		ACT	-2.06 (-6.20, 2.05)	
Mean (SD)	16.7 (10.2)	14.2 (8.7)	12.5 (6.6)	12.0 (6.7)	11.6 (6.5)	12.6 (7.7)		Time	-0.01 (-0.04, 0.01)	
Control								ACT*time	-0.01 (-0.04, 0.02)	
N	32	25	25	21	19	16				
Mean (SD)	16.4 (9.3)	14.3 (8.8)	16.1 (8.7)	13.5 (11.4)	14.1 (8.7)	13.5 (8.7)				
P-value	0.986	0.888	0.182	0.957	0.526	0.843				0.65 (0.420)
**AADQ**										
ACT								Intercept	53.40 (50.50, 56.30)	
N	39	24	21	19	17	17		ACT	4.80 (0.80, 8.82)	
Mean (SD)	57.4 (8.8)	58.7 (7.6)	59.6 (7.7)	59.0 (9.2)	62.1 (8.5)	61.2 (8.3)		Time	0.03 (0.01, 0.05)	
Control								ACT*time	0.01 (-0.02, 0.04)	
No.	32	25	25	21	19	16				

For peer review only

Mean (SD)	52.6 (11.3)	54.3 (11.3)	54.5 (9.6)	58.3 (7.8)	56.5 (7.1)	57.8 (5.9)				
P value	0.049	0.207	0.047	0.560	0.022	0.073				0.34(0.562)
**MANSA**										
ACT								Intercept	4.65 (4.38, 4.91)	
N	39	24	21	19	17	17		ACT	0.44 (0.07, 0.80)	
Mean (SD)	5.0 (0.9)	5.1 (0.8)	5.3 (0.8)	5.1 (0.9)	5.3 (0.6)	5.3 (0.9)		Time	0.00 (0.00, 0.00)	
Control								ACT*time	0.00 (0.00, 0.01)	
N	32	25	25	21	19	16				
Mean (SD)	4.7 (0.7)	4.7 (1.0)	4.5 (0.9)	4.8 (0.9)	4.8 (0.9)	4.5 (0.8)				
P value	0.093	0.118	0.006	0.266	0.038	0.015				5.79(0.016)
**SDSCA-GD**										
ACT								Intercept	4.14 (3.61, 4.67)	
No.	39	24	21	19	17	17		ACT	0.75 (0.02, 1.48)	
Mean (SD)	5.0 (1.6)	4.7 (1.8)	4.5 (1.7)	4.9 (1.6)	5.1 (1.5)	5.1 (1.5)		Time	0.01 (0.00, 0.01)	
Control								ACT*time	0.00 (-0.01, 0.01)	
No.	32	25	25	21	19	16				
Mean (SD)	4.1 (1.9)	3.8 (2.1)	4.4 (1.8)	4.9 (1.4)	4.9 (1.8)	4.7 (1.6)				
P value	0.062	0.104	0.722	0.743	0.784	0.388				0.01(0.924)
**SDSCA- SD**										
ACT								Intercept	3.66 (3.18, 4.15)	
N	39	24	21	19	17	17		ACT	0.21 (-0.46, 0.88)	
Mean (SD)	3.8 (1.8)	4.1 (1.5)	3.6 (1.4)	4.5 (1.2)	4.2 (1.6)	4.1 (1.7)		Time	0.00 (-0.01, 0.00)	
Control								ACT*time	0.00 (0.00, 0.01)	
No.	32	25	25	21	19	16				

For peer review only

Mean (SD)	3.5 (1.6)	3.7 (1.6)	3.7 (1.6)	4.2 (1.5)	3.7 (1.5)	3.6 (1.6)				
P value	0.313	0.415	0.706	0.405	0.425	0.269				1.37 (0.242)
**SDSCA-E**										
ACT								Intercept	2.62 (2.03, 3.22)	
No.	39	24	21	19	17	17		ACT	0.73 (-0.10, 1.55)	
Mean (SD)	3.2 (2.1)	4.2 (1.9)	3.2 (2.0)	3.8 (2.3)	4.1 (2.1)	3.6 (2.2)		Time	0.00 (0.00, 0.01)	
Control								ACT*time	0.00 (-0.01, 0.01)	
N	32	25	25	21	19	16				
Mean (SD)	2.5 (2.0)	2.7 (2.0)	2.5 (1.9)	2.9 (1.8)	3.2 (2.6)	2.9 (1.7)				
P value	0.174	0.010	0.186	0.253	0.245	0.536				0.10 (0.749)
**SDSCA -G**										
ACT								Intercept	5.37 (4.83, 5.91)	
N	39	24	21	19	17	17		ACT	0.84 (0.09, 1.59)	
Mean (SD)	6.1 (1.8)	6.3 (1.2)	6.3 (1.4)	6.6 (0.9)	6.9 (0.3)	6.8 (0.8)		Time	0.01 (0.00, 0.01)	
Control								ACT*time	0.00 (-0.01, 0.01)	
N	32	25	25	21	19	16				
Mean (SD)	5.4 (2.5)	5.5 (2.3)	5.7 (2.2)	6.0 (1.7)	6.0 (1.9)	6.7 (0.8)				
P value	0.423	0.300	0.613	0.265	0.117	0.924				0.23 (0.634)

ACT, acceptance and commitment therapy; SD, standard deviation; CI, confidence interval; HbA1c, glycated haemoglobin; Scale; HFS, Hypoglycaemia Fear Survey (HFS- W-Worry, HFS-B- Behaviour); AADQ, Acceptance Action Diabetes Questionnaire; MANSA, Manchester Short Assessment of Quality of life; SDSCA, Summary of Diabetes Self-Care Activities (SDSCA-GD-general diet, SDSCA-SD-specific diet, SDSCA-E-Exercise, SDSCA-G-Glucose monitoring).

For peer review only

Supplementary Table 4: ACT exposure (number of sessions attended) and proportion of participants with HbA1c measurement in each timepoint

**ACT exposure**	**Frequency** Intervention group (n =43)	**Frequency** Control group (n=38)
Zero sessions	8	-
One session	3	-
Two sessions	5	-
Three sessions	7	-
Four sessions	1	-
Five sessions	3	-
Six sessions	9	-
Seven sessions	7	-
**HbA1c measurement**		
Week 8, n (%)	27 (62.8)	20 (52.6)
Week 14, n (%)	21 (48.8)	21 (55.3)
6 months, n (%)	15 (34.9)	15 (39.5)
12 months, n (%)	16 (37.2)	17 (44.7)
24 months, (%)	15 (34.9)	11 (28.9)

ACT, acceptance and commitment therapy; HbA1c, glycated Haemoglobin.

For peer review only

BMJ Open

Impact of an Acceptance and Commitment Therapy programme on HbA1c, self-management and psychosocial factors in adults with type 1 diabetes and elevated HbA1c levels: a randomised controlled trial

Journal:	*BMJ Open*
Manuscript ID	bmjopen-2023-072061.R1
Article Type:	Original research
Date Submitted by the Author:	21-Sep-2023
Complete List of Authors:	Wijk, Ingrid ; Sophiahemmet University, Department of Health Promoting Science<br>Amsberg, Susanne; Marie Cedershiöld University, Department of Health Care Sciences<br>Johansson, Unn-Britt; Sophiahemmet University, Department of Health Promoting Science; Karolinska Institutet, Department of Clinical Sciences and Education, Södersjukhuset<br>Livheim, Fredrik; Karolinska Institutet, Department of Clinical Neuroscience<br>Toft, Eva; Karolinska Institutet, Department of Medicine, Huddinge; Karolinska Institutet, Department of Medicine, Ersta Hospital<br>Anderbro, Therese; Karolinska Institutet, Department of Clinical Sciences; Stockholm University, Department of Psychology
**Primary Subject Heading**:	Diabetes and endocrinology
Secondary Subject Heading:	Mental health
Keywords:	DIABETES & ENDOCRINOLOGY, Depression & mood disorders < PSYCHIATRY, PREVENTIVE MEDICINE

SCHOLARONE™
Manuscripts

I, the Submitting Author has the right to grant and does grant on behalf of all authors of the Work (as defined in the below author licence), an exclusive licence and/or a non-exclusive licence for contributions from authors who are: i) UK Crown employees; ii) where BMJ has agreed a CC-BY licence shall apply, and/or iii) in accordance with the terms applicable for US Federal Government officers or employees acting as part of their official duties; on a worldwide, perpetual, irrevocable, royalty-free basis to BMJ Publishing Group Ltd ("BMJ") its licensees and where the relevant Journal is co-owned by BMJ to the co-owners of the Journal, to publish the Work in this journal and any other BMJ products and to exploit all rights, as set out in our licence.

The Submitting Author accepts and understands that any supply made under these terms is made by BMJ to the Submitting Author unless you are acting as an employee on behalf of your employer or a postgraduate student of an affiliated institution which is paying any applicable article publishing charge ("APC") for Open Access articles. Where the Submitting Author wishes to make the Work available on an Open Access basis (and intends to pay the relevant APC), the terms of reuse of such Open Access shall be governed by a Creative Commons licence – details of these licences and which Creative Commons licence will apply to this Work are set out in our licence referred to above.

Other than as permitted in any relevant BMJ Author's Self Archiving Policies, I confirm this Work has not been accepted for publication elsewhere, is not being considered for publication elsewhere and does not duplicate material already published. I confirm all authors consent to publication of this Work and authorise the granting of this licence.

For peer review only

Impact of an Acceptance and Commitment Therapy programme on HbA1c, self-management and psychosocial factors in adults with type 1 diabetes and elevated HbA1c levels: a randomised controlled trial

Ingrid Wijk[1*], Susanne Amsberg[2], Unn-Britt Johansson[1,3], Fredrik Livheim[4], Eva Toft[5,6], Therese Anderbro[7,8]

[1]Department of Health Promoting Science, Sophiahemmet University, Stockholm, Sweden

[2]Department of Health Care Sciences, Marie Cederschiöld University, Stockholm, Sweden

[3]Department of Clinical Sciences and Education, Södersjukhuset, Karolinska Institutet, Stockholm, Sweden

[4]Department of Clinical Neuroscience, Karolinska Institutet, Stockholm, Sweden

[5]Department of Medicine, Huddinge, Karolinska Institutet, Stockholm, Sweden

[6]Department of Medicine, Karolinska Institutet, Ersta Hospital, Stockholm, Sweden

[7]Department of Clinical Sciences, Danderyd Hospital, Karolinska Institutet, Stockholm, Sweden

[8]Department of Psychology, Stockholm University, Stockholm, Sweden

For peer review only

*Corresponding author:

E-mail address: ingrid.wijk@shh.se (I. Wijk).

Sophiahemmet University

P O Box 5605

SE-114 86 Stockholm, Sweden

ABSTRACT

Objective: To evaluate the impact of an Acceptance and Commitment Therapy (ACT) programme, tailored for people living with type 1 diabetes, on HbA1c, self-management and psychosocial factors among individuals with HbA1c >60 mmol/mol compared with treatment as usual (TAU).

Setting: An endocrinologic clinic in Sweden.

Participants: In this randomised controlled trial, 81 individuals with type 1 diabetes, aged 18-70 years with HbA1c >60 mmol/mol, were randomly assigned to either an ACT group intervention or TAU. Exclusion criteria were pregnancy, severe untreated psychiatric illness, disease or medical treatment that may affect the glucose level and newly started insulin pump treatment. At the two-year follow-up, HbA1c was measured in 26 individuals.

Intervention: The ACT programme comprised seven 2-hour sessions held over 14 weeks and focused on acceptance of stressful thoughts and emotions, and to promote value-based committed action.

Outcomes: The primary outcome was HbA1c, and the secondary outcomes were measures of depression, anxiety, general stress, fear of hypoglycaemia, diabetes distress, self-care activities, psychological flexibility (general and related to diabetes) and quality of life. The primary endpoint was HbA1c two years after the intervention programme. Linear mixed models were used to test for an interaction effect between measurement time and group.

Results: Likelihood ratio test of nested models demonstrated no statistically significant interaction effect (chi-square=1, p=0.316) between measurement time and group regarding HbA1c. However, a statistically significant interaction effect (likelihood ratio test chi-square=12.63, p<0.001) was observed with improved scores on The Acceptance and Action Questionnaire in the intervention group after one and two years.

Conclusions: No statistically significant difference was found between the groups regarding the primary outcome measure, HbA1c. However, the ACT programme showed a persistent beneficial impact on psychological flexibility in the intervention group.

Trial registration: Trial registration number NCT02914496.

Strengths and limitations of this study:

- This study was developed using an interdisciplinary approach, with a randomised control trial design.
- The intervention is theory-based, with roots in behavioural research, and is tailored for living with diabetes.
- This research addresses psychological aspects of living with type 1 diabetes which has been requested by the diabetes community and in international guidelines.

- The drop-out rate in this study affects the possibility to draw conclusions from the findings.

For peer review only - http://bmjopen.bmj.com/site/about/guidelines.xhtml

For peer review only

BACKGROUND

Type 1 diabetes is an autoimmune mediated disease (1) with an increasing global incidence in young people under 20 years of age (2). Complications of type 1 diabetes involve life-threating conditions such as hypoglycaemia and ketoacidosis as well as micro- and macro vascular diseases (3). National and international guidelines recommend treatment targets for glycaemic level, individually adjusted, to prevent development of complications (4, 5). However, only a minority reach the treatment targets (6-8).

The pancreatic beta cell destruction in type 1 diabetes brings a life-long dependency on supplying exogenous insulin (3) and along with that a self-management posing high demands (9). Glucose monitoring, insulin adjustment and managing hypo- and hyperglycaemia are examples of self-care activities involved (10). In addition to the amount of ingested carbohydrates, several factors such as physical activity, hormones, stress and temperature affect the glycaemic level (10). Consequently, self-management requires knowledge and skills (11) and involves frequent decision-making (12). Emotional stress related to the management of diabetes, diabetes distress, persists over time (13) and is present among 20%–30% of people with type 1 diabetes (14). Furthermore, the prevalence of depression is up to three-fold higher among individuals living with type 1 diabetes (15). Moreover, depression in individuals with diabetes is linked with an increased risk of mortality (16). Several studies have found that psychological health problems and elevated HbA1c levels are associated (17-19). Consequently, there has been a call to address the psychological aspects of living with diabetes (20, 21).

Cognitive Behaviour Therapy (CBT) is focused on restructuring maladaptive thoughts to create more functional cognition and behaviours (22). CBT has shown positive effects on depression in a general population (23) as well as among individuals with diabetes (24). In a randomised controlled trial (RCT) by Amsberg et al., a CBT intervention improved HbA1c and reduced levels of depression and diabetes distress (25).

Acceptance and Commitment Therapy (ACT) expands upon CBT. The approach in ACT suggests that the presence of uncomfortable thoughts and feelings should be accepted and not supressed or modified owing to the fact that human life is difficult (26). Despite the existence of uncomfortable thoughts, functional behaviours could pave the way to a life in accordance with a person's values and goals. Being present and aware, and at the same time developing functional behaviours adapted in the current context, is referred to as psychological flexibility (27). Moreover, psychological flexibility, which ACT aims to increase (28), is suggested to be a key prerequisite to psychological health (29). ACT is based on six flexibility processes: acceptance - allowance of difficult thoughts and feelings, defusion- not getting fused with thoughts and feelings, self-as-context – observing thoughts and feelings, attention to the present moment – being mindful of the current internal and external environment, values – stating personal values that may provide direction in life and commitment – committed action - creating behaviours that corresponds to personal values (27). Previous studies have supported treatment effects of ACT for different mental and somatic health problems (30). With its focus on acceptance of discomfort, ACT has also been argued to be especially suitable for long-term conditions where distress can be a realistic factor (31). Furthermore, a recent study found that greater psychological flexibility in adults with type 1 diabetes was associated with less distress and more beneficial HbA1c (32). A meta-analysis concluded that ACT interventions may improve the ability of self-care, psychological flexibility and HbA1c in type 2 diabetes

For peer review only - http://bmjopen.bmj.com/site/about/guidelines.xhtml

(33). Within the scope of type 1 diabetes, few ACT interventions for adults have been evaluated. A feasibility study of a single-arm trial involving individuals with type 1 diabetes and comorbid eating disorder indicated positive effects on psychological flexibility and diabetes distress, in addition to being feasible and improving eating disorder symptoms. Furthermore, an RCT feasibility study of an ACT-based intervention provided in webformat included individuals with type 1 and type 2 diabetes. A reduction in diabetes distress was seen in the intervention group. However, the dropout rate was 57% during treatment (34).

In summary, type 1 diabetes is a severe disease with an increased prevalence of impaired psychological health with treatment targets reached by only a minority. Previous research regarding ACT and diabetes has mainly focused on type 2 diabetes. Our hypothesis was that an ACT programme would lead to improvements in glycaemic and psychosocial outcomes in an adult population with type 1 diabetes and prolonged elevation of HbA1c. Thus, we conducted an RCT aiming to evaluate the impact of an ACT programme, tailored for people living with type 1 diabetes, on HbA1c, self-management and psychosocial factors.

METHODS

Study design and participants

This study was a longitudinal two-arm RCT conducted at an endocrinologic clinic in Sweden. The intervention group participated in an ACT programme and the control group received treatment as usual (TAU) only. The endocrinology clinic population comprised adults (> 18 years) with type 1 diabetes and included urban and suburban residents. A study protocol has been published where a more detailed description of the method and the intervention is provided (35).

Eligible participants were identified by extracting data from the Swedish National Diabetes Register (NDR). Individuals who met the inclusion criteria: type 1 diabetes with a duration of at least 2 years, aged 18–70 years and HbA1c > 60 mmol/mol on two occasions during preceding year, were assessed in a random order (generated by computer). An additional inclusion criterion was the ability to speak, read and write in Swedish. Sequentially, patient records were reviewed for information on exclusion criteria. The following criteria were applied: severe, untreated mental illness, disease or treatment that can affect glucose level (e.g., steroid treatment, thyrotoxicosis, planned bariatric surgery), pregnancy and newly started insulin pump treatment (within three months).

Recruitment

A power analysis before recruitment suggested that at least 56 individuals with type 1 diabetes were needed to reach 80% power for detecting a clinically relevant reduction in HbA1c of 6 mmol/mol (with assumed standard deviation [SD] of 9) at a 5% significance level. To allow for dropouts of approximately 24% (25) the aim was to recruit 70 persons with type 1 diabetes.

From 2016 to 2019 individuals with HbA1c >60 mmol/mol (reference value 42 mmol/mol) were assessed for eligibility (n=622) in five rounds. Study information and an invitation to participate were sent by post to eligible individuals who met the criteria for inclusion (n=343). Two weeks after the material was sent, a research nurse assistant telephoned the invited individuals to offer more information about the study, if desired. Eighty-one people submitted written informed consent and were included in the study.

For peer review only - http://bmjopen.bmj.com/site/about/guidelines.xhtml

Consequently, the participation rate was 23.6% (81 of 343 who received an invitation). Sociodemographic details and information on treatment were collected at baseline.

Randomisation

After the baseline measurements, participants were randomly allocated into either the intervention or control group (Figure 1). A person not involved with the project conducted randomisation by drawing sealed lots. Stratification was carried out to achieve an equal distribution by sex in the groups. The research assistant nurse informed participants of the group assignment by e-mail and provided further instructions three weeks before the start of the intervention programme. Blinding the ACT-instructors or the research assistant nurse to the group allocations during the study period was not possible. However, the ACT-instructors never met the control group; additionally, the research nurse assistant was not involved in the intervention programme and did not have ACT skills.

Intervention

This study was developed by a multidisciplinary team consisting of psychologists, nurses specialised in diabetes and an endocrinologist. The ACT programme consisted of seven two-hour sessions that were held every other week. A text message was sent the day before each session as a reminder. The size of the ACT groups ranged from six to nine participants. Furthermore, the programme was manual-based and developed according to a general ACT programme (36). The manual was tailored by a psychologist specialised in diabetes and a diabetes nurse to direct the focus to living with type 1 diabetes; both were trained ACT instructors. The sessions involved activities connected to the six core components of ACT: - being present, acceptance, defusion, self as context, values, and commitment (27). Recurring activities throughout the programme were group discussions, psychoeducation, exercises, mindfulness practice, role play and value awareness. There was also homework between sessions.

The intervention group followed the ACT programme alongside TAU and the control group received TAU. TAU comprised a minimum of two visits (one appointment with an endocrinologist and one with a nurse specialised in diabetes) at the clinic each year. The needs of the patient governed the number of visits and telephone contacts.

Assessments

Time points for measurement were at baseline, weeks 8 and 14 during the intervention and repeated measurements at 6 months, one year and two years after the intervention. Web-based questionnaires were sent to participants by e-mail and reminders were sent one and two weeks after the first e-mail, if needed. The questionnaires and reminders were sent via a web-based tool. Three weeks before each follow-up assessment, a letter with information regarding the coming measurements and dates for HbA1c measurement was sent via post and via e-mail. Reminders of the upcoming dates were sent a couple of days before HbA1c measurement, which took place close to the clinic but not in the hospital area. If both the questionnaires and HbA1c measurement were completed, a gift card of 100 Swedish crowns was sent to the participant after each time point of measurement.

Primary outcome measure

The primary outcome was glycated haemoglobin, HbA1c. The measurement reflects the average glycaemic level during the previous two to three months (37). The treatment goal should be individually

For peer review only - http://bmjopen.bmj.com/site/about/guidelines.xhtml

set; however, a general treatment target for adults is < 53 mmol/mol (38). We used capillary blood samples and equipment to conduct point-of-care immunoassay (DCA Vantage Analyzer, Siemens) which has shown sufficient correlation with the reference method, high-performance liquid chromatography (39). The method of analysis is calibrated against IFCC standard (International Federation of Clinical Chemists). The same equipment was used at each measurement time point and a checklist for calibration and maintenance was followed in accordance with the manufacturer's recommendations (40).

Secondary outcome measures

Depression Anxiety Stress Scales

Depression, anxiety and stress, were assessed with The Depression Anxiety, Stress Scale (DASS21) (41). The questionnaire consists of 21 items divided into three subscales. A 4-point Likert scale, starting with *didn't apply to me at all* and ending with *applied to me very much or most of the time,* is used for rating (41). Psychometric properties have been evaluated in a Swedish population (42). Cronbach's alpha in the subscales in the current study ranged from 0.75 to 0.92.

Hypoglycaemia Fear Survey

Fear of hypoglycaemia was assessed by using The Hypoglycaemia Fear Survey (HFS) (41, 43). The scale with 23 items in total involves two subscales: worry and behaviour. A 5-point Likert Scale is used for scoring (0=never, 4=always). A higher score indicates a more explicit fear of hypoglycaemia (43). The Swedish version has been evaluated regarding psychometric properties (44). Cronbach's alpha for the total scale used in the current study was 0.86.

Problem Areas in Diabetes Scale

The Problem Areas in Diabetes Scale (PAID) is a measure for assessing emotional distress related to diabetes (45). The questionnaire consists of 20 items and is scored on a 5-point Likert scale (0=not a problem to 4= serious problem) (45). Greater distress is indicated with higher scores (total score 0-100) where 40 is the cut-off for significant distress (46, 47). The reliability and validity of the Swedish version scale have been evaluated (48). Cronbach`s alpha in the current study was 0.91.

Summary of Diabetes Self-Care Activities

The frequency of self-care activities over the past seven days was measured with the Summary of Diabetes Self-Care Activities (SDSCA) (49). The questionnaire includes eleven items in different areas of self-care activity. The scoring system ranges from 0 to 7 and refers to the number of weekdays. The areas are separately scored and are not summed in a total score. High scores indicate high self-care performance (49). A Swedish version, translated by Amsberg et al., was used which also demonstrated sufficient internal consistency for the subscales (Cronbach's alpha 0.80 – 0.86) in a similar sample (25). In the current study, Cronbach's alpha for the subscales diet, exercise and glucose monitoring ranged from 0.80 to 0.90.

For peer review only - http://bmjopen.bmj.com/site/about/guidelines.xhtml
For peer review only

Acceptance and Action Questionnaire

The original Acceptance and Action Questionnaire (AAQ) (50) was developed into a revised version, the AAQ-II (51) to measure the degree of psychological flexibility. The revised scale AAQ-II has been translated into Swedish and evaluated regarding psychometric features. This version was used in our study. There are six items in the Swedish version that are rated on a 7-point Likert scale (1= never true, 7 =always true). A lower score reflects greater psychological flexibility (52). Cronbach`s alpha in the current study was 0.88.

Acceptance Action Diabetes Questionnaire

Psychological flexibility related to diabetes was assessed using the Acceptance Action Diabetes Questionnaire (AADQ). The questionnaire is derived from the AAQ and was developed by Gregg et al. (53). The original AADQ comprises eleven items and is rated on a 7-point Likert scale (1= never true, 7= always true). A higher score indicates a greater psychological flexibility (53) in contrast to the AAQ which is scored in the opposite direction (52). The scale was translated into Swedish, and the psychometric properties has been evaluated. The manuscript has been accepted but is not yet published. Cronbach`s alpha in the current study was 0.77.

Manchester Short Assessment of Quality of life

The Manchester Short Assessment of Quality of life (MANSA) is used to assess quality of life (QoL). The questionnaire was developed by Priebe et al. (54) and was constructed with 16 items (12 items related to general QoL and 4 are objective items of QoL). The rating score ranges from 1 to 7 (1= could not be worse, 7 = could not be better). The response categories in the objective items are dichotomous (yes, no). A Swedish version has been evaluated regarding the psychometric properties (55). The Cronbach`s alpha in the current study was 0.85.

Statistical methods

Baseline characteristics were described as mean (SD) for continuous variables and absolute and relative frequencies for categorical variables. Tests for differences in baseline characteristics between completers and non-completers and intervention (control group, intervention group) were performed using the Kruskal-Wallis test (continuous variables) and chi-squared test (categorical variables). Mixed effect models were fitted to test for changes in HbA1c levels and questionnaire scores across follow-up. Each model included the time point (baseline, week 8, week 14, week 38, week 62) and intervention (control group, intervention group) as fixed effects and a random intercept for participants. Interaction effects between the time point and intervention were explored. Test of fixed effects were done using a likelihood ratio test in nested models using the chi-square distribution. We implemented a compound symmetry structure within our mixed-effects model. Graphical assessment of residuals plotted against predicted values were conducted to validate the model’s assumption.

For peer review only - http://bmjopen.bmj.com/site/about/guidelines.xhtml

All statistical analyses were performed using R version 3.6.2 (56). IBM SPSS 27 was used for graphs (IBM Corp., Armonk, NY, USA). Other statistical analyses that were planned and presented in the study protocol could not be conducted or were not suitable owing to a high number of missing values and lack of statistical significance in outcome. The intention-to-treat principle was applied in the primary analysis. In addition, a per-protocol analysis was conducted in a further examination of the results.

Fidelity check of the intervention

For quality assurance, a fidelity check was performed by videotaping one session held by the licensed psychologist and the registered nurse. The videotape was reviewed by DE, an experienced trainer who has been training instructors in the ACT-group intervention that was used. In addition, participants in two groups and the group leaders estimated the adherence to the treatment intervention and manual by completing an adherence evaluation after sessions three and seven. The aim was to measure how adherent each participant had been to the treatment interventions in the sessions and how well the group leaders adhered to the treatment manual. The group leaders also evaluated their adherence to the manual. Finally, participants' and group leaders' evaluations were compared. Participants and group leaders rated each intervention in the session on a scale from 0 to 10 where 0 = "this has not been discussed or brought up", to 10 = "This was covered thoroughly". The estimates are shown in Supplementary Table 1 and Supplementary Table 2 (Supplementary Material).

Insert figure 1

PATIENT AND PUBLIC INVOLVEMENT

The RCT was preceded by an informal pre-trial among eight volunteers with type 1 diabetes to test the design and content of the ACT intervention. On the basis of the feedback of participants, an adaptation of the programme was made with shorter but more sessions. The participants in the RCT will receive a message when the results are published and where to find them. There will also be a link provided to a webpage with a summary of the results in plain Swedish.

RESULTS

Study participants

The mean age for all study participants in the sample was 40.2 (SD 16.6) years and 70% were women. The mean duration of type 1 diabetes was 23.4 (SD 14.4) years, the mean HbA1c was 73.5 (SD 13) mmol/mol and 74.7% of participants have had retinopathy on some occasion. Approximately 88% of participants used sensor-based glucose monitoring and 35.8% wore an insulin pump. In Table 1 the baseline characteristics are described for the total sample and the allocated trial groups. No statistically significant differences between group characteristics were found at baseline. Baseline scoring of the secondary outcomes and comparisons between the groups are shown in Supplementary Table 3 and Supplementary Table 4 (Supplementary Material). No statistically significant differences were found

For peer review only - http://bmjopen.bmj.com/site/about/guidelines.xhtml

between the groups in baseline scoring (Bonferroni correction applied). Significantly elevated diabetes distress was present among 51% of the study sample, (PAID score >40). Mean scores in the normal range were noted for stress and anxiety. The mean score in the depression subscale of 12.14 (SD 10.85) indicated mild depression, and 39% of participants had scores corresponding to moderate to severe depression.

For peer review only

Table 1: Baseline characteristics of trial groups with comparisons between complete (participants completing the two-year follow-up) and drop-out (participants lost to follow-up or who discontinued the study at the two-year follow-up) groups.

Characteristics	All participants	Intervention group	Control group	P value	Complete	Dropout	P value
**N**	81	43	38		26	55	
**Males**, n (%)	30 (37)	15 (34.9)	15 (39.5)	0.844	8 (30.8)	22 (40)	0.578
**Age** (years) (SD, range)	40.2 (16.6, 19-69)	42.2 (16.5, 19-69)	37.9 (16.6, 20-68)	0.368	38.6 (16.4)	40.9 (16.8)	0.754
**Diabetes duration** (years) (SD, range)	23.4 (14.4, 3-61)	23.6 (14.0, 3-61)	23.2 (15, 5-61)	0.730	20.2 (12.5)	24.9 (15.1)	0.156
**Family/Cohabitation**							
With a partner, n (%)	36 (44.4)	17 (39.5)	19 (50)		11 (42.3)	25 (45,5)	
With parents, n (%)	14 (17.3)	7 (16.3)	7 (18.4)		3 (11.5)	11 (20.0)	
With other, n (%)	5 (6.2)	2 (4.7)	3 (7.9)		0 (0.0)	5 (9.1)	
Alone, n (%)	22 (27.2)	13 (30.2)	9 (23.7)		11 (42.3)	11 (20.0)	
Apart, n (%)	4 (4.9)	4 (9.3)	0 (0.0)	0.314	1 (3.8)	3 (5.5)	0.167
**Education (highest level)**							
Elementary school, n (%)	4 (4.9)	3 (7.0)	1 (2.6)		0 (0.0)	4 (7.3)	
Upper secondary school, n (%)	30 (37.0)	14 (32.6)	16 (42.1)		8 (30.8)	22 (40.0)	
University (courses or education) n (%)	41 (50.6)	24 (55.8)	17 (44.7)		16 (61.5)	25 (45.5)	
Other, n (%)	6 (7.4)	2 (4.7)	4 (10.5)	0.441	2 (7.7)	4 (7.3)	0.361
**Employment**							
Retired, n (%)	6 (7.4)	3 (7.0)	3 (7.9)		1 (3.8)	5 (9.1)	
Early retirement, n (%)	5 (6.2)	3 (7.0)	2 (5.3)		3 (11.5)	2 (3.6)	
Student, n (%)	11 (13.6)	5 (11.6)	6 (15.8)		3 (11.5)	8 (14.5)	
Not currently working for medical reasons, n (%)	2 (2.5)	2 (4.7)	0 (0.0)		0 (0.0)	2 (3.6)	
Unemployed, n (%)	3 (3.7)	1 (2.3)	2 (5.3)		1 (3.8)	2 (3.6)	

For peer review only - http://bmjopen.bmj.com/site/about/guidelines.xhtml

Employed, n (%)	44 (54.3)	21 (48.8)	23 (60.5)		15 (57.7)	29 (52.7)	
Manage own company, n (%)	10 (12.3)	8 (18.6)	2 (5.3)	0.420	3 (11.5)	7 (12.7)	0.730
**CGM or FGM**, n (%)	71 (87.7)	37 (86)	34 (89.5)	0.897	24 (92.3)	47 (85.5)	0.608
**CSII**, n (%)	29 (35.8)	15 (34.9)	14 (36.8)	1.000	12 (46.2)	17 (30.9)	0.277
**BMI** (SD, range)	26.1 (4.7, 17.3-39)	25.5 (4.5, 17.3-36.5)	26.8 (4.7, 19.6-39)	0.250	26.6 (3.4)	25.8 (5.1)	0.210
**Baseline HbA1c** (mmol/mol) (SD, range)	73.5 (13, 60-125)	71.4 (10.1, 60-118)	75.9 (15.4, 61-125)	0.315	69.2 (7.2)	75.5 (14.6)	0.029
**Retinopathy,** n (%) *	59 (74.7)	30 (73.2)	29 (76.3)	0.950	17 (65.4)	42 (79.2)	0.291
**Neuropathy**, n (%) *	23 (29.1)	13 (31.7)	10 (26.3)	0.780	9 (34.6)	14 (26.4)	0.624
**Nephropathy,** n (%) *							
Micro-albuminuria	4 (5.2)	2 (5.0)	2 (5.4)		2 (7.7)	2 (3.9)	
Macro-albuminuria	2 (2.6)	1 (2.5)	1 (2.7)		0 (0.0)	2 (3.9)	
No albuminuria	71 (92.2)	37 (92.5)	34 (91.9)	0.995	24 (92.3)	47 (92.2)	0.474
**Cerebrovascular disease**, n (%) *	1 (1.3)	1 (2.4)	0 (0.0)	1.000	0 (0)	1 (1.9)	1.000
**Coronary artery disease,** n (%) *	3 (3.8)	1 (2.4)	2 (5.3)	0.946	1 (3.8)	2 (3.8)	1.000

*Information missing for some individuals (nephropathy:4, neuropathy:2, retinopathy:2, cerebrovascular disease:4, coronary artery disease:2)

SD, standard deviation; BMI, body mass index; HbA1c, glycated haemoglobin; CGM, continuous glucose monitoring; FGM, flash glucose monitoring; CSII, continuous subcutaneous insulin infusion .

For peer review only

For peer review only - http://bmjopen.bmj.com/site/about/guidelines.xhtml

Dropouts and adherence to the programme

Eight participants did not start the intervention programme and wished to withdraw from the study. Reasons for their decision were as follows: internship in another city (n=1), going abroad (n=1), lack of time (n=1), family reasons (n=1), and having to work (n=1). In three cases, no reason was provided. Reasons for interrupting the programme were as follows: disliked the program (n=2), medical/health related reasons (n= 3), evening time not suitable (n = 1), family reasons (n= 1), and change in working schedule (n=1). One person in the intervention group passed away before the two-year follow-up. In the control group, the reasons reported for discontinuation of the study after randomisation until the last timepoint for measurement (two years) were as follows: medical reasons (n= 1), family reasons (n=1), no longer interested (n=2), lack of time (n=1) and moved to another city (n=2). Parts of the one-year and the two-year follow-up were conducted during the COVID-19 pandemic during 2020-2021. Three participants actively declined the invitation for follow-up measurement because of the pandemic (two who had symptoms and one owing to the risk of infection). However, one HbA1c measurement could be registered from the Endocrinology clinic in the same week. Most reasons for discontinuation/loss to follow-up were unknown.

Supplementary Table 5 (Supplementary Material) presents the exposure to ACT. Thirty-five participants attended at least one session (81.4%) and 20 participants (46.5%) attended four to seven sessions of the programme. The attendance for measurement of HbA1c is also shown in Supplementary Table 5. In week 8 after session 4, 62.8% of participants in the intervention group and 52.6% controls completed HbA1c measurement. The dropout rate was approximately equal in the trial arms during the follow-up period. Furthermore, no significant differences in baseline characteristics were detected among participants remaining in the study after two years and those who were lost to follow-up (Bonferroni correction applied) (Table 1).

Primary outcome

The mean HbA1c in the intervention group decreased over two years from 71.4 mmol/mol to 59.6 mmol/mol. However, the likelihood ratio test of nested models demonstrated no statistically significant interaction effect (chi-square:1, p:0.316) between measurement time point and group affiliation regarding HbA1c, as shown in Supplementary Table 3 (Supplementary Material). The observed mean HbA1c over time is illustrated in Figure 2.

Insert Figure 2.

Secondary outcomes

A significant interaction effect (likelihood ratio test chi-square:12.63, p<0.001) between time and group allocation was found in AAQ-II scores measuring psychological flexibility (Supplementary Table 3,

For peer review only - http://bmjopen.bmj.com/site/about/guidelines.xhtml

Supplementary Material). This finding indicates a trend of improvement in the ACT group over time. The observed mean AAQ-II score over two years is illustrated in Figure 3.

Insert Figure 3

No significant interaction effects between time and group allocation were demonstrated in scores of the PAID, DASS21 (Supplementary Table 3, Supplementary Material), HFS, AADQ, MANSA or SDSCA (Supplementary Table 4, Supplementary Material).

Per protocol

Per protocol analyses were conducted; the results were consistent with findings in the intention-to-treat analyses.

DISCUSSION

This study evaluated the impact of an ACT programme tailored for individuals living with type 1 diabetes. The observed mean values decreased to a level below 60 mmol/mol in the intervention group during the study period. However, no statistically significant effect on HbA1c was demonstrated two years after the ACT program. The AAQ-II scores decreased over time and the improvement in psychological flexibility was statistically significant after one and two years. The results regarding HbA1c correspond to those of a meta-analysis involving overall psychological interventions in type 1 diabetes (57) and a meta-analysis of CBT interventions for diabetes related distress in type 1 and type 2 diabetes (58). Some CBT intervention studies solely focusing on type 1 diabetes have shown similar findings (59, 60). At the same time, the meta-analysis of Winkley et. al (2020) and Yang et al (2020) concluded that CBT has the potential to be effective for HbA1c reduction in type 1 diabetes (57, 61) which was clearly demonstrated in the study of Amsberg et .al (2008). Regarding ACT intervention in diabetes the findings in our study stand in contrast to those of previous studies. ACT interventions in type 2 diabetes have shown promising results (33). A recent study of an ACT intervention for adolescents with type 1 diabetes showed a significant beneficial effect on HbA1c (62). To our knowledge, this is one of the first RCTs to evaluate the effect of a group-based face-to-face ACT intervention on HbA1c in an adult population. We used a longitudinal design with a long-term follow-up period of two years which can enlighten the mechanism of change over time. The study protocol was developed according to Standard Protocol Items: Recommendations for Interventional Trials (SPIRIT) 2013 Statement (63) and was previously published and open for transparency and replication. We used instruments that have been evaluated for psychometric properties (with one exception, the translated version of the SDSCA), and the same device for HbA1c measurements.

Interestingly, a persistent beneficial effect was found in psychological flexibility during the follow-up period. The effect was seen one year after the intervention but not before that time. This finding may indicate that psychological flexibility takes time to develop; this was also discussed in a previous study

For peer review only - http://bmjopen.bmj.com/site/about/guidelines.xhtml

(64). We were unable to conduct mediation analysis regarding the role of psychological flexibility in self-management and HbA1c improvement owing to the absence of statistical significance in the outcome variables. However, a development process that requires time would hypothetically generate improvement in HbA1c even later in the follow-up period. Notably, the statistically significant change in psychological flexibility was only observed in the generic AAQ-II and not in the disease-specific AADQ. The AAQ-II is the most frequently used instrument in research for measuring psychological flexibility, but it has also been criticised for being a measure for psychological distress rather than of psychological flexibility (65).

Reflections have been made regarding the connection between the content of the psychological intervention and diabetes self-management in explaining outcomes in similar studies (57, 62). Gregg et al. (2007) evaluated the impact of an educational intervention with and without ACT components in type 2 diabetes (53). The current study did not focus on self-management and did not involve elements of diabetes education, which may have been a disadvantage. Another observed aspect that differentiates the current study from other group-based studies showing HbA1c improvement is the individualised perspective. The intervention in the study by Amsberg et al. (2008) included individual meetings (25), and Alho et al. (2022) conducted interviews regarding expectations prior to the intervention (62). Moreover, participants called for more individualisation in a feasibility trial of a web-based ACT programme (34). An example of a beneficial individualised element in psychological intervention trials is a study by Malin et al. (2020). A brief motivational interview was introduced after the initial session which increased adherence and decreased the drop-out rate in a CBT intervention of chronic pain and cancer (66).

This study has several limitations, among which the main limitation is the dropout rate. Eight individuals (18.6%) allocated to the ACT group did not start the intervention which is in line with a meta-analysis of dropout rates in CBT trials (67). However, the dropout rate during the ACT programme period in the current study far exceeded the aggregated dropout rate in the study by Fernandez et. al (2015) (67). In the study by Bendig et al. (2021) (34) that involved a web-based ACT programme, the dropout rate was 57% in the intervention group during the study period which is more similar to our findings. Nevertheless, there are no indications of different dropout rates between trials based on ACT or other psychological interventions (68). In addition, the Covid-19 pandemic may have affected the attendance for measurement of HbA1c during parts of the one-year and two-years follow-up periods. There were no differences in baseline characteristics between participants who remained at the two-year follow-up and those who were lost to follow-up at the same measurement time point. Without a Bonferroni correction, the HbA1c demonstrated higher values in the group lost to follow-up which would imply a selection bias. Furthermore, the consequence of the high attrition is an underpowered analysis, which may entail an elevated risk of type II error. Another deficiency in the study is the lack of a comprehensive process evaluation such as that Craig et al. (2008) described in the Medical Research Council guidelines for complex interventions (69). On the other hand, we have gained valuable knowledge regarding the context and implementation of ACT groups for adults with type 1 diabetes that lays a foundation for further development. This study indicates that the format of providing tools based on ACT may not be suitable for the targeted study population. The mean age of the participants suggests a life context with demands from family and work life. In addition, previous research has showed that high HbA1c and executive dysfunctions are associated, at least in young adulthood (70). It is possible that comprehensive programmes for behaviour change which requirements of commitment may be

For peer review only - http://bmjopen.bmj.com/site/about/guidelines.xhtml

difficult to fit in a busy life. Brief ACT interventions have demonstrated promising results in different disciplines (71). It is also noteworthy that diabetes distress may be a significant issue for persons with HbA1c within target range (72). Thus, interventions targeting diabetes distress, with elements of brief ACT strategies incorporated in diabetes health care may be beneficial to explore.

Considering that this was a single-center study with significant attrition problems, the external validity is weakened. The study is ongoing with the last measurement timepoint at five years after the intervention. The decision to proceed with follow-up measurements despite the high attrition was made owing to the effects of psychological flexibility and tendencies toward a decreasing HbA1c.

In conclusion, this study showed no statistically significant difference between the groups regarding the primary outcome measure, HbA1c. However, the ACT programme showed a persistent beneficial impact on psychological flexibility in the intervention group.

Author contributions

Study conceptualisation and design: TA, SA, FL, ET. Acquisition of data: IW, ET. Analysis and interpretation of data: IW in collaboration with an external statistician and TA, U-BJ, SA. Manuscript draft: IW. Revision and approval of final draft: TA, U-BJ, SA, ET, FL.

Ethics approval

This study was approved by the Swedish Ethical Review Authority (2016/14-31/1).

Funding

The study was supported by Lindhés advokatbyrå AB (grant nr: LA2016-0429), the Annie and Fritz Tjus donation fund (grant nr: N/A), the Sophiahemmet Foundation (grant nr: N/A) and the Swedish Diabetes Foundation (grant nr: DIA2018-360).

Competing interests

FL receives royalties from his books written on ACT and income from training professionals in ACT.

Data sharing statement

The data may be available upon reasonable request.

Acknowledgement

We would like to thank all participants in the study and RN Neide Johansson for supporting the recruitment and data collection. Furthermore, we would like to acknowledge Fredrik Wiklund (Statisticon AB) for statistical support and Psychologist Daniel Ek (DE) for the fidelity check of the videotaped material. We also thank Edanz for language editing.

REFERENCES

1. WHO. Classification of Diabetes Mellitus. Geneva: WHO; 2019.

For peer review only - http://bmjopen.bmj.com/site/about/guidelines.xhtml

2. Patterson CC, Karuranga S, Salpea P, Saeedi P, Dahlquist G, Soltesz G, et al. Worldwide estimates of incidence, prevalence and mortality of type 1 diabetes in children and adolescents: Results from the International Diabetes Federation Diabetes Atlas, 9th edition. Diabetes Res Clin Pract. 2019;157:107842. https://doi.org/10.1016/j.diabres.2019.107842
3. DiMeglio LA, Evans-Molina C, Oram RA. Type 1 diabetes. Lancet. 2018;391(10138):2449-62. https://doi.org/10.1016/S0140-6736(18)31320-5
4. American Diabetes Association. 6. Glycemic Targets: Standards of Medical Care in Diabetes—2022. Diabetes Care. 2021;45(Supplement_1):S83-S96. https://doi.org/10.2337/dc22-S006
5. The National Board of Health and Welfare. Nationella riktlinjer för diabetesvård - Stöd för styrning och ledning. Report No.: 978-91-75555-476-1. Socialstyrelsen 2018 www.socialstyrelsen.se
6. The Swedish National Diabetes Register. Nationella Diabetesregistrets årsrapport 2021. www.ndr.nu
7. Shah VN, Grimsmann JM, Foster NC, Dost A, Miller KM, Pavel M, et al. Undertreatment of cardiovascular risk factors in the type 1 diabetes exchange clinic network (United States) and the prospective diabetes follow-up (Germany/Austria) registries. Diabetes Obes Metab. 2020;22(9):1577-85. https://doi.org/10.1111/dom.14069
8. Renard E, Ikegami H, Daher Vianna AG, Pozzilli P, Brette S, Bosnyak Z, et al. The SAGE study: Global observational analysis of glycaemic control, hypoglycaemia and diabetes management in T1DM. Diabetes Metab Res Rev. 2021;37(7):e3430. https://doi.org/10.1002/dmrr.3430
9. Funnell MM, Bootle S, Stuckey HL. The diabetes attitudes, wishes and needs second study. Clin Diabetes. 2015;33(1):32-6. https://doi.org/10.2337/diaclin.33.1.32
10. Reddy M, Rilstone S, Cooper P, Oliver NS. Type 1 diabetes in adults: supporting self management. BMJ. 2016;352:i998. https://doi.org/10.1136/bmj.i998
11. Iqbal A, Novodvorsky P, Heller SR. Recent Updates on Type 1 Diabetes Mellitus Management for Clinicians. Diabetes Metab J. 2018;42(1):3-18. https://doi.org/10.4093/dmj.2018.42.1.3
12. Jull J, Witteman HO, Ferne J, Yoganathan M, Stacey D. Adult-Onset Type 1 Diabetes: A Qualitative Study of Decision-Making Needs. Can J Diabetes. 2016;40(2):164-9. https://doi.org/10.1016/j.jcjd.2015.09.080
13. Fisher L, Hessler D, Polonsky W, Strycker L, Masharani U, Peters A. Diabetes distress in adults with type 1 diabetes: Prevalence, incidence and change over time. J Diabetes Complications. 2016;30(6):1123-8. https://doi.org/10.1016/j.jdiacomp.2016.03.032
14. Sturt J, Dennick K, Due-Christensen M, McCarthy K. The detection and management of diabetes distress in people with type 1 diabetes. Curr Diab Rep. 2015;15(11):101. https://doi.org/10.1007/s11892-015-0660-z
15. Roy T, Lloyd CE. Epidemiology of depression and diabetes: a systematic review. J Affect Disord. 2012;142 Suppl:S8-21. https://doi.org/10.1016/S0165-0327(12)70004-6
16. van Dooren FE, Nefs G, Schram MT, Verhey FR, Denollet J, Pouwer F. Depression and risk of mortality in people with diabetes mellitus: a systematic review and meta-analysis. PloS one. 2013;8(3):e57058. https://doi.org/10.1371/journal.pone.0057058
17. de Groot M, Golden SH, Wagner J. Psychological conditions in adults with diabetes. Am Psychol. 2016;71(7):552-62. https://doi.org/10.1037/a0040408
18. Hessler DM, Fisher L, Polonsky WH, Masharani U, Strycker LA, Peters AL, et al. Diabetes distress is linked with worsening diabetes management over time in adults with Type 1 diabetes. Diabet Med. 2017;34(9):1228-34. https://doi.org/10.1111/dme.13381
19. Melin EO, Thunander M, Svensson R, Landin-Olsson M, Thulesius HO. Depression, obesity, and smoking were independently associated with inadequate glycemic control in patients with type 1 diabetes. Eur J Endocrinol. 2013;168(6):861-9. https://doi.org/10.1530/EJE-13-0137

For peer review only - http://bmjopen.bmj.com/site/about/guidelines.xhtml

20. Jones A, Vallis M, Pouwer F. If it does not significantly change HbA1c levels why should we waste time on it? A plea for the prioritization of psychological well-being in people with diabetes. Diabet Med. 2015;32(2):155-63. https://doi.org/10.1111/dme.12620
21. Holt RIG, DeVries JH, Hess-Fischl A, Hirsch IB, Kirkman MS, Klupa T, et al. The management of type 1 diabetes in adults. A consensus report by the American Diabetes Association (ADA) and the European Association for the Study of Diabetes (EASD). Diabetologia. 2021;64(12):2609-52. https://doi.org/10.1007/s00125-021-05568-3
22. Fenn K, Byrne, M. . The key principals of cognitive behaviour therapy. InnovAiT: Education and inspiration for general practice. 2013;6(9). https://doi.org/10.1177/1755738012471029
23. Lepping P, Whittington R, Sambhi RS, Lane S, Poole R, Leucht S, et al. Clinical relevance of findings in trials of CBT for depression. Eur Psychiatry 2017;45:207-11. https://doi.org/10.1016/j.eurpsy.2017.07.003
24. Uchendu C, Blake H. Effectiveness of cognitive-behavioural therapy on glycaemic control and psychological outcomes in adults with diabetes mellitus: a systematic review and meta-analysis of randomized controlled trials. Diabet Med. 2017;34(3):328-39. https://doi.org/10.1111/dme.13195
25. Amsberg S, Anderbro T, Wredling R, Lisspers J, Lins PE, Adamson U, et al. A cognitive behavior therapy-based intervention among poorly controlled adult type 1 diabetes patients--a randomized controlled trial. Patient Educ Couns. 2009;77(1):72-80. https://doi.org/10.1016/j.pec.2009.01.015
26. Hayes S, Strosahl K, Wilson K. Acceptance and Commitment Therapy: The Process and Practice of Mindful Change. New York: Guilford Press 2011.
27. Hayes SC, Levin ME, Plumb-Vilardaga J, Villatte JL, Pistorello J. Acceptance and Commitment Therapy and Contextual Behavioral Science: Examining the Progress of a Distinctive Model of Behavioral and Cognitive Therapy. Behav Ther. 2013;44(2):180-98. https://doi.org/10.1016/j.beth.2009.08.002
28. Ruiz F. A review of Acceptance and Commitment Therapy (ACT) empirical evidence: Correlational, experimental psychopathology, component and outcome studies. International Journal of Psychology and Psychological Therapy. 2010;10:125-62.
29. Kashdan TB, Rottenberg J. Psychological flexibility as a fundamental aspect of health. Clin Psychol Rev. 2010;30(7):865-78. https://doi.org/10.1016/j.cpr.2010.03.001
30. A-Tjack J, Davis ML, Morina N, Powers MB, Smits JA, Emmelkamp PM. A meta-analysis of the efficacy of acceptance and commitment therapy for clinically relevant mental and physical health problems. Psychother Psychosom. 2015;84(1):30-6. https://doi.org/10.1159/000365764
31. Graham CD, Gouick J, Krahé C, Gillanders D. A systematic review of the use of Acceptance and Commitment Therapy (ACT) in chronic disease and long-term conditions. Clin Psychol Rev. 2016;46:46-58. https://doi.org/10.1016/j.cpr.2016.04.009
32. Nicholas JA, Yeap BB, Cross D, Burkhardt MS. Psychological flexibility is associated with less diabetes distress and lower glycated haemoglobin in adults with type 1 diabetes. Intern Med J. 2022;52(6):952-8. https://doi.org/10.1111/imj.15250
33. Sakamoto R, Ohtake Y, Kataoka Y, Matsuda Y, Hata T, Otonari J, et al. The efficacy of acceptance and commitment therapy for people with type 2 diabetes: Systematic review and meta-analysis. J Diabetes Investig. 2021. https://doi.org/10.1111/jdi.13658
34. Bendig E, Bauereiss N, Schmitt A, Albus P, Baumeister H. ACTonDiabetes-a guided psychological internet intervention based on Acceptance and Commitment Therapy (ACT) for adults living with type 1 or 2 diabetes: results of a randomised controlled feasibility trial. BMJ open. 2021;11(7):e049238-e. https://doi.org/10.1136/bmjopen-2021-049238
35. Amsberg S, Wijk I, Livheim F, Toft E, Johansson UB, Anderbro T. Acceptance and commitment therapy (ACT) for adult type 1 diabetes management: study protocol for a randomised controlled trial. BMJ Open. 2018;8(11):e022234. https://doi.org/10.1136/bmjopen-2018-022234

For peer review only - http://bmjopen.bmj.com/site/about/guidelines.xhtml

36. Livheim F. ACT treatment for youth – a contextual behavioral approach. [dissertation] Stockholm: Karolinska Institutet 2019. https://openarchive.ki.se/xmlui/bitstream/handle/10616/46394/Thesis_Fredrik_Livheim.pdf?sequence=3&isAllowed=y
37. Jeffcoate SL. Diabetes control and complications: the role of glycated haemoglobin, 25 years on. Diabet Med. 2004;21(7):657-65. https://doi.org/10.1046/j.1464-5491.2003.01065.x
38. American Diabetes Association. 6. Glycemic Targets: Standards of Medical Care in Diabetes—2021. Diabetes Care. 2020;44(Supplement_1):S73-S84. https://doi.org/10.2337/dc21-S006
39. Guadalupe Vargas M, Pazmiño Gomez BJ, Vera Lorenti FE, Álvarez Condo GM, Rodas Neira EI, Veron D, et al. Assessment of two glycated hemoglobin immunoassays. Endocrinol Diabetes Nutr (English ed). 2020;67(5):297-303. https://doi.org/10.1016/j.endinu.2019.09.011
40. Healthineers S. DCA Vantage Analyzer: Siemens Healthineers; [cited 2022 24-10]. Available from: https://www.siemens-healthineers.com/se/diabetes/diabetes/dca-vantage-analyzer
41. Lovibond PF, Lovibond SH. The structure of negative emotional states: comparison of the Depression Anxiety Stress Scales (DASS) with the Beck Depression and Anxiety Inventories. Behav Res Ther. 1995;33(3):335-43. https://doi.org/10.1016/0005-7967(94)00075-u
42. Alfonsson S, Wallin E, Maathz P. Factor structure and validity of the Depression, Anxiety and Stress Scale-21 in Swedish translation. J Psychiatr Ment Health Nurs. 2017;24(2-3):154-62. https://doi.org/10.1111/jpm.12363
43. Cox DJ, Irvine A, Gonder-Frederick L, Nowacek G, Butterfield J. Fear of hypoglycemia: quantification, validation, and utilization. Diabetes Care. 1987;10(5):617-21. https://doi.org/10.2337/diacare.10.5.617
44. Anderbro T, Amsberg S, Wredling R, Lins PE, Adamson U, Lisspers J, et al. Psychometric evaluation of the Swedish version of the Hypoglycaemia Fear Survey. Patient Educ Couns. 2008;73(1):127-31. https://doi.org/10.1016/j.pec.2008.03.022
45. Polonsky WH, Anderson BJ, Lohrer PA, Welch G, Jacobson AM, Aponte JE, et al. Assessment of diabetes-related distress. Diabetes Care. 1995;18(6):754-60. https://doi.org/10.2337/diacare.18.6.754
46. Hermanns N, Kulzer B, Krichbaum M, Kubiak T, Haak T. How to screen for depression and emotional problems in patients with diabetes: comparison of screening characteristics of depression questionnaires, measurement of diabetes-specific emotional problems and standard clinical assessment. Diabetologia. 2006;49(3):469-77. https://doi.org/10.1007/s00125-005-0094-2
47. Welch GW, Jacobson AM, Polonsky WH. The Problem Areas in Diabetes Scale. An evaluation of its clinical utility. Diabetes Care. 1997;20(5):760-6. https://doi.org/10.2337/diacare.20.5.760
48. Amsberg S, Wredling R, Lins PE, Adamson U, Johansson UB. The psychometric properties of the Swedish version of the Problem Areas in Diabetes Scale (Swe-PAID-20): scale development. Int J Nurs Stud. 2008;45(9):1319-28. https://doi.org/10.1016/j.ijnurstu.2007.09.010
49. Toobert DJ, Hampson SE, Glasgow RE. The summary of diabetes self-care activities measure: results from 7 studies and a revised scale. Diabetes Care. 2000;23(7):943-50. https://doi.org/10.2337/diacare.23.7.943
50. Hayes S, Strosahl K, Wilson K, Bissett RT, Pistorello J, Toarmino D, et al. Measuring experiential avoidance: A preliminary test of a working model. The Psychological record. 2004;54:553-78. https://doi.org/10.1007/BF03395492
51. Bond FW, Hayes SC, Baer RA, Carpenter KM, Guenole N, Orcutt HK, et al. Preliminary psychometric properties of the Acceptance and Action Questionnaire-II: a revised measure of psychological inflexibility and experiential avoidance. Behav Ther. 2011;42(4):676-88. https://doi.org/10.1016/j.beth.2011.03.007

For peer review only - http://bmjopen.bmj.com/site/about/guidelines.xhtml

52. Lundgren T, Parling T. Swedish Acceptance and Action Questionnaire (SAAQ): a psychometric evaluation. Cogn Behav Ther. 2017;46(4):315-26. https://doi.org/10.1080/16506073.2016.1250228
53. Gregg JA, Callaghan GM, Hayes SC, Glenn-Lawson JL. Improving diabetes self-management through acceptance, mindfulness, and values: a randomized controlled trial. J Consult Clin Psychol. 2007;75(2):336-43. https://doi.org/10.1037/0022-006X.75.2.336
54. Priebe S, Huxley P, Knight S, Evans S. Application and results of the Manchester Short Assessment of Quality of Life (MANSA). Int J Soc psychiatry. 1999;45(1):7-12. https://doi.org/10.1177/002076409904500102
55. Björkman T, Svensson B. Quality of life in people with severe mental illness. Reliability and validity of the Manchester Short Assessment of Quality of Life (MANSA). Nord J Psychiatry. 2005;59(4):302-6. https://doi.org/10.1080/08039480500213733
56. R Core Team. R: A language and environment for statistical computing. Vienna, Austria.: R Foundation for Statistical Computing; 2019 [Available from: https://www.R-project.org.]
57. Winkley K, Upsher R, Stahl D, Pollard D, Brennan A, Heller S, et al. Systematic review and meta-analysis of randomized controlled trials of psychological interventions to improve glycaemic control in children and adults with type 1 diabetes. Diabet Med. 2020;37(5):735-46. https://doi.org/10.1111/dme.14264
58. Jenkinson E, Knoop I, Hudson JL, Moss-Morris R, Hackett RA. The effectiveness of cognitive behavioural therapy and third-wave cognitive behavioural interventions on diabetes-related distress: A systematic review and meta-analysis. Diabet Med. 2022;39(11):e14948. https://doi.org/10.1111/dme.14948
59. Snoek FJ, van der Ven NC, Twisk JW, Hogenelst MH, Tromp-Wever AM, van der Ploeg HM, et al. Cognitive behavioural therapy (CBT) compared with blood glucose awareness training (BGAT) in poorly controlled Type 1 diabetic patients: long-term effects on HbA moderated by depression. A randomized controlled trial. Diabet Med. 2008;25(11):1337-42. https://doi.org/10.1111/j.1464-5491.2008.02595.x
60. van der Ven NC, Hogenelst MH, Tromp-Wever AM, Twisk JW, van der Ploeg HM, Heine RJ, et al. Short-term effects of cognitive behavioural group training (CBGT) in adult Type 1 diabetes patients in prolonged poor glycaemic control. A randomized controlled trial. Diabet Med. 2005;22(11):1619-23. https://doi.org/10.1111/j.1464-5491.2005.01691.x
61. Yang X, Li Z, Sun J. Effects of Cognitive Behavioral Therapy–Based Intervention on Improving Glycaemic, Psychological, and Physiological Outcomes in Adult Patients With Diabetes Mellitus: A Meta-Analysis of Randomized Controlled Trials. Front Psychiatry. 2020;11(711). https://doi.org/10.3389/fpsyt.2020.00711
62. Alho I, Lappalainen P, Muotka J, Lappalainen R. Acceptance and commitment therapy group intervention for adolescents with type 1 diabetes: A randomized controlled trial. J Contextual Behav Sci. 2022;25:153-61. https://doi.org/10.1016/j.jcbs.2022.08.002
63. Chan AW, Tetzlaff JM, Altman DG, Laupacis A, Gøtzsche PC, Krleža-Jerić K, et al. SPIRIT 2013 statement: defining standard protocol items for clinical trials. Ann Intern Med. 2013;158(3):200-7. https://doi.org/10.7326/0003-4819-158-3-201302050-00583
64. Fledderus M, Bohlmeijer ET, Smit F, Westerhof GJ. Mental health promotion as a new goal in public mental health care: a randomized controlled trial of an intervention enhancing psychological flexibility. Am J Public Health. 2010;100(12):2372. https://doi.org/10.2105/AJPH.2010.196196
65. Cherry KM, Hoeven EV, Patterson TS, Lumley MN. Defining and measuring "psychological flexibility": A narrative scoping review of diverse flexibility and rigidity constructs and perspectives. Clin Psychol Rev. 2021;84:101973. https://doi.org/10.1016/j.cpr.2021.101973
66. Malins S, Biswas S, Rathbone J, Vogt W, Pye N, Levene J, et al. Reducing dropout in acceptance and commitment therapy, mindfulness-based cognitive therapy, and problem-solving therapy for

For peer review only - http://bmjopen.bmj.com/site/about/guidelines.xhtml

chronic pain and cancer patients using motivational interviewing. Br J Clin Psychol. 2020;59(3):424-38. https://doi.org/10.1111/bjc.12254
67. Fernandez E, Salem D, Swift J, Ramtahal N. Meta-Analysis of Dropout From Cognitive Behavioral Therapy: Magnitude, Timing, and Moderators. J Consult Clin Psychol. 2015;83. https://doi.org/10.1037/ccp0000044
68. Ong CW, Lee EB, Twohig MP. A meta-analysis of dropout rates in acceptance and commitment therapy. Behav Res Ther. 2018;104:14-33. https://doi.org/10.1016/j.brat.2018.02.004
69. Craig P, Dieppe P, Macintyre S, Michie S, Nazareth I, Petticrew M. Developing and evaluating complex interventions: the new Medical Research Council guidance. BMJ. 2008;337:a1655. https://doi.org/10.1136/bmj.a1655

70. Ding K, Reynolds CM, Driscoll KA, Janicke DM. The Relationship Between Executive Functioning, Type 1 Diabetes Self-Management Behaviors, and Glycemic Control in Adolescents and Young Adults. Curr Diab Rep. 2021;21(3):10. https://doi.org/10.1007/s11892-021-01379-3

71. Dindo L, Van Liew JR, Arch JJ. Acceptance and Commitment Therapy: A Transdiagnostic Behavioral Intervention for Mental Health and Medical Conditions. Neurotherapeutics. 2017;14(3):546-53. https://doi.org/10.1007/s13311-017-0521-3

72. Todd PJ, Edwards F, Spratling L, Patel NH, Amiel SA, Sturt J, et al. Evaluating the relationships of hypoglycaemia and HbA1c with screening-detected diabetes distress in type 1 diabetes. Endocrinology, diabetes & metabolism. 2018;1(1):e00003. https://doi.org/10.1002/edm2.3

For peer review only

For peer review only - http://bmjopen.bmj.com/site/about/guidelines.xhtml

Figure 1: Flow diagram of recruitment, randomisation and attrition

HbA1c, glycated haemoglobin.

Figure 2: Mean values of HbA1c over two years by group affiliation

ACT, acceptance and commitment therapy; HbA1c, glycated haemoglobin; CI, confidence interval.

Figure 3: Mean values of AAQ-II score over two years by group affiliation

AAQ, Acceptance and Action Questionnaire; HbA1c, glycated haemoglobin; CI, confidence interval.

For peer review only

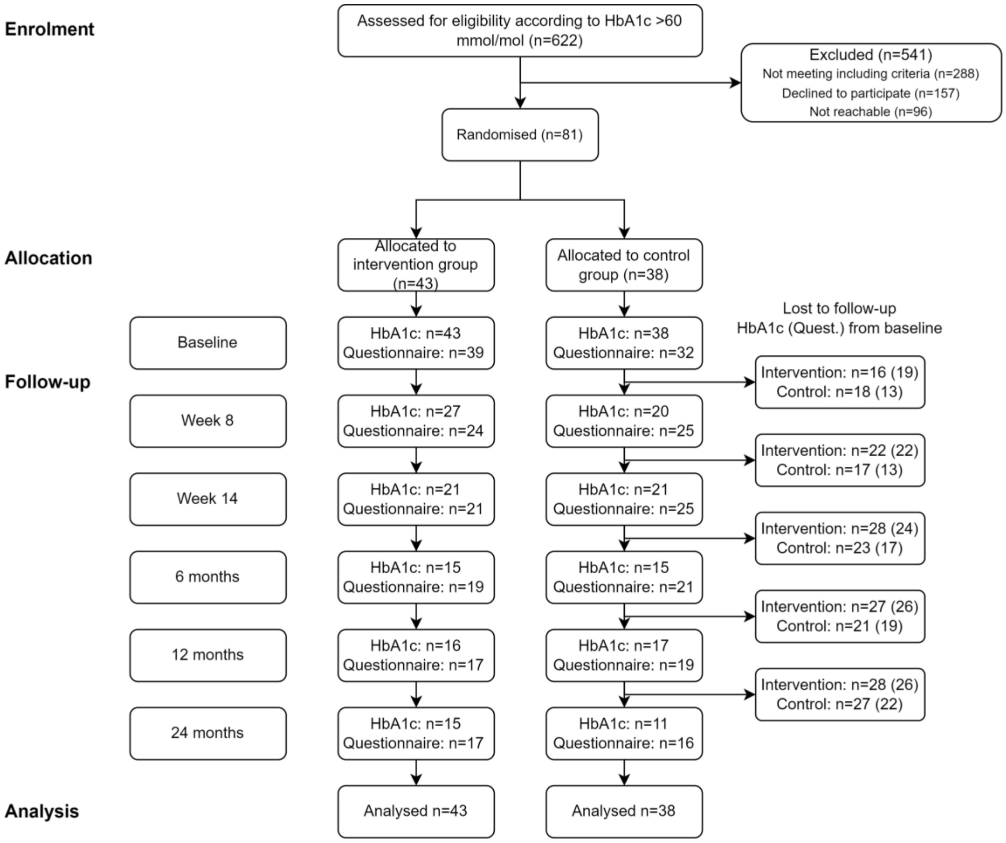

Flow diagram of recruitment, randomisation and attrition
HbA1c, glycated haemoglobin.

1058x882mm (72 x 72 DPI)

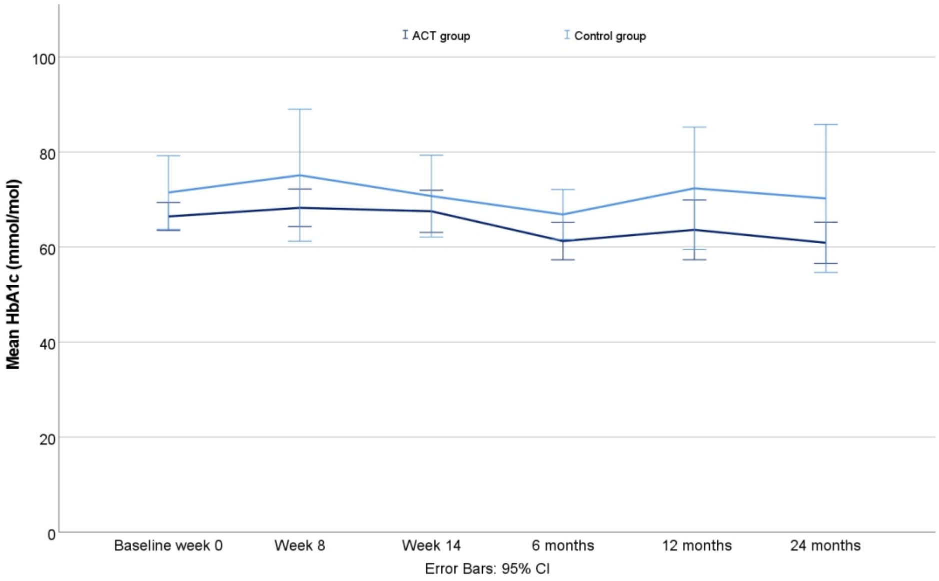

Figure 2: Mean values of HbA1c over two years by group affiliation
ACT, acceptance and commitment therapy; HbA1c, glycated haemoglobin; CI, confidence interval.

1128x661mm (72 x 72 DPI)

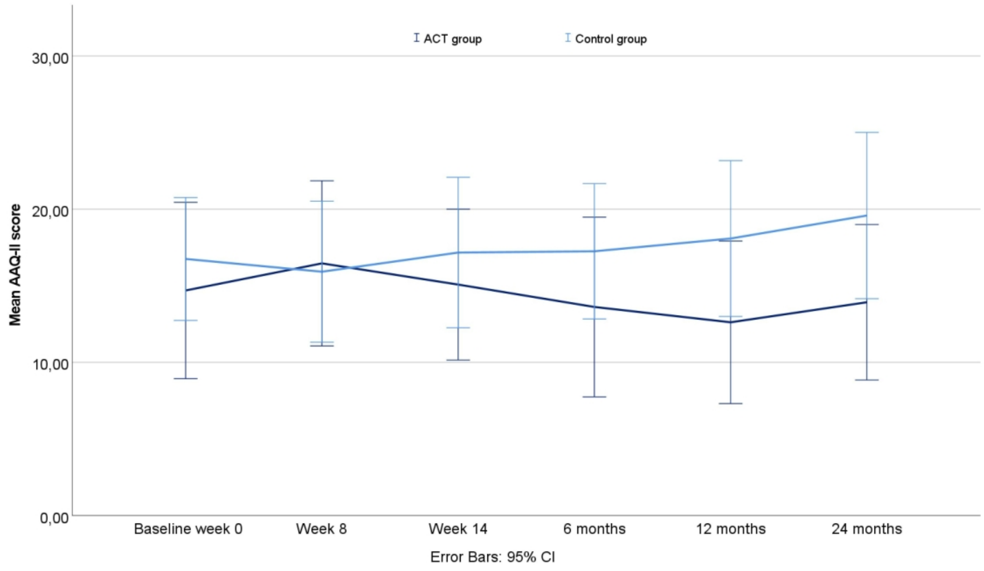

Figure 3: Mean values of AAQ-II score over two years by group affiliation
AAQ, Acceptance and Action Questionnaire; HbA1c, glycated haemoglobin; CI, confidence interval.

1128x662mm (72 x 72 DPI)

Supplementary Table 1: Estimates of the adherence among the instructors (n=2) to the ACT programme.

	Group A (n=5) s 3 median (range)	Instructors s 3 median (range)	Group A (n=5) s 7 median (range)	Instructors s 7 median (range)	Group B (n=3) s 3 median (range)	Instructors s 3 median (range)
Review of preceding sessions	8 (6-9)	10 (10-10)	9 (9-10)	8 (8-8)	7 (6-9)	9 (9-9)
Feedback on homework	7 (5-10)	7.5 (7-8)	9 (8-10)	6.5 (6-7)	8 (7-10)	8 (7-9)
The Compass of life	10 (7-10)	7.5 (7-8)	9 (9-10)	8 (8-8)	9 (7-10)	9.5 (9-10)
Specific exercise	9.5 (8-10)	8.5 (8-9)	10 (8-10)	9 (9-9)	9 (6-10)	9.5 (9-10)

Estimations on a scale 1-10 (0= "this has not been discussed/brought up", to 10 = "This was covered thoroughly".)
Examples of items from the adherence evaluation. s 3: session 3, s 7: session 7

Supplementary Table 2: Estimates of the adherence among participants in two groups to the ACT programme.

	Group A (n=5) s 3 median(range)	Group A (n=5) s 7 median(range)	Group B (n=3) s 3 median(range)
Made changes in life[1]	2 (1-2)	2.5 (2-3)	2 (2-3)
App use (mindfulness)[2]	0 (0-2)	1 (0-1)	2 (0-10)
Mindfulness daily life[3]	3 (2-4)	3.5 (2-4)	4 (3-4)

1. Have you since the course started, decided to change something/take steps in your life direction? 1=No, 2=I have decided and made a small step/change,3=I have decided and made several changes/steps.

2. How many times per week did you practise mindfulness with the app "I´m here now"?

3. How often do you practise mindfulness in your daily life? 1=Never,2=Seldom, 3=Once a week/sometimes,4=Once per day, 5=Several times a day

s 3: session 3, s 7: session 7

For peer review only

Supplementary Table 3: Observed mean value at each time point for outcome measurements and linear mixed model analysis with testing for interaction effects of time and ACT intervention on HbA1c, PAID, DASS21 and AAQ-II.

							Mixed model analysis with interaction		
	**Week 0**	**Week 8**	**Week 14**	**6 months**	**12 months**	**24 months**	**Fixed effects**	**Estimate (95% CI)**	**Interaction effect χ^2 (p value)**
**HbA1c** mmol/mol									
ACT							Intercept	75.22 (71.43, 79.01)	
N	43	27	21	15	16	15	ACT	-3.71 (-8.90, 1.49)	
Mean (SD)	71.4 (10.1)	70.1 (8.9)	69.4 (8.3)	63.6 (9.5)	64.6 (8.4)	59.6 (8.0)	Time	-0.05 (-0.09, -0.01)	
Control							ACT*time	-0.02 (-0.07, 0.03)	
N	38	20	21	15	17	11			
Mean (SD)	75.9 (15.4)	75.7 (14.4)	72.4 (11.9)	64.6 (5.7)	70.2 (13.3)	70.5 (18.6)			
P-value	0.315	0.281	0.227	0.677	0.181	0.132			0.49 (0.485)
**PAID**									
ACT							Intercept	43.52 (37.78, 49.29)	
N	39	24	21	19	17	17	ACT	-9.40 (-17.36, -1.46)	
Mean (SD)	36.6 (18.1)	32.6 (19.7)	28.7 (18.7)	31.1 (18.5)	28.6 (16.0)	27.4 (18.6)	Time	-0.05 (-0.10, -0.01)	
Control							ACT*time	-0.01 (-0.08, 0.05)	
N	32	25	25	21	19	16			
Mean (SD)	42.9 (15.9)	39.8 (20.7)	41.6 (19.2)	34.9 (20.9)	36.2 (16.6)	33.4 (17.7)			
P value	0.229	0.322	0.034	0.533	0.188	0.279			0.22(0.642)
**DASS-S**									
ACT							Intercept	16.53 (13.63, 19.44)	
N	39	24	21	19	17	17	ACT	-4.90 (-8.92, -0.89)	
Mean (SD)	12.5 (8.4)	12.5 (9.2)	9.8 (8.3)	12.8 (10.4)	9.4 (6.3)	10.7 (6.7)	Time	-0.01 (-0.03, 0.02)	
Control							ACT*time	-0.01 (-0.04, 0.03)	
N	32	25	25	21	19	16			
Mean (SD)	16.8 (11.1)	14.5 (10.7)	15.8 (9.7)	13.0 (10.9)	14.3 (11.3)	14.1 (10.7)			

P value	0.141	0.534	0.036	0.978	0.226	0.458				0.11(0.743)
**DASS-A**										
ACT								Intercept	7.54 (5.44, 9.64)	
N	39	24	21	19	17	17		ACT	-1.06 (-3.97, 1.85)	
Mean (SD)	6.8 (6.8)	5.8 (7.1)	5.4 (6.3)	7.3 (8.3)	4.2 (4.0)	4.6 (7.9)		Time	0.01 (-0.01, 0.03)	
Control								ACT*time	-0.02 (-0.05, 0.01)	
N	32	25	25	21	19	16				
Mean (SD)	8.2 (7.1)	6.6 (7.0)	6.9 (6.0)	6.5 (7.0)	8.0 (6.7)	7.1 (6.2)				
P value	0.302	0.460	0.283	0.763	0.042	0.063				1.97(0.161)
**DASS-D**										
ACT								Intercept	13.10 (9.87, 16.35)	
N	39	24	21	19	17	17		ACT	-2.53 (-7.03, 1.95)	
Mean (SD)	11.7 (11.4)	10.9 (11.0)	6.9 (9.4)	11.6 (12.8)	6.0 (7.4)	10.0 (7.5)		Time	0.02 (-0.01, 0.06)	
Control								ACT*time	-0.03 (-0.08, 0.01)	
N	32	25	25	21	19	16				
Mean (SD)	12.6 (10.3)	11.3 (9.0)	14.1 (10.0)	11.1 (11.7)	14.8 (11.3)	14.1 (10.5)				
P value	0.486	0.672	0.015	0.989	0.011	0.261				2.00(0.157)
**AAQ**										
ACT								Intercept	17.69 (15.34, 20.05)	
N	39	24	21	19	17	17		ACT	-1.95 (-5.20, 1.30)	
Mean (SD)	16.3 (8.4)	15.8 (8.5)	14.2 (8.3)	15.0 (9.3)	11.9 (7.8)	12.5 (7.8)		Time	0.02 (0.00, 0.04)	
Control								ACT*time	-0.05 (-0.08, -0.02)	
N	32	25	25	21	19	16				
Mean (SD)	18.1 (6.1)	16.0 (7.0)	17.4 (7.3)	16.7 (7.2)	17.9 (7.9)	18.9 (7.8)				
P value	0.325	0.749	0.095	0.356	0.016	0.013				12.63 (<0.001)

*DASS-S-stress, DASS-A-anxiety, DASS-D-depression

ACT, acceptance and commitment therapy; SD, standard deviation; CI, confidence interval; HbA1c, glycated haemoglobin; PAID, Problem Areas in Diabetes Scale; DASS, Depression, Anxiety, Stress Scale; AAQ, Acceptance and Action Questionnaire.

For peer review only

Supplementary Table 4: Observed mean value at each time point for outcome measurements and linear mixed model analysis with testing for interaction effects of time and ACT intervention on HFS, AADQ, MANSA and SDSCA

**HFS-B**										
ACT								Intercept	20.27 (18.61, 21.93)	
N	39	24	21	19	17	17		ACT	-3.14 (-5.44, -0.85)	
Mean (SD)	18.1 (4.9)	15.8 (5.2)	15.7 (4.6)	15.9 (4.4)	15.4 (4.5)	16.7 (6.8)		Time	-0.02 (-0.03, 0.00)	
Control								ACT*time	0.01 (-0.01, 0.03)	
N	32	25	25	21	19	16				
Mean (SD)	20.4 (5.3)	19.5 (4.9)	19.5 (6.7)	18.7 (6.0)	18.7 (5.8)	19.6 (5.3)				
P-value	0.030	0.013	0.021	0.162	0.177	0.139				0.55 (0.457)
**HFS-W**										
ACT								Intercept	17.00 (14.02, 19.99)	
N	39	24	21	19	17	17		ACT	-2.06 (-6.20, 2.05)	
Mean (SD)	16.7 (10.2)	14.2 (8.7)	12.5 (6.6)	12.0 (6.7)	11.6 (6.5)	12.6 (7.7)		Time	-0.01 (-0.04, 0.01)	
Control								ACT*time	-0.01 (-0.04, 0.02)	
N	32	25	25	21	19	16				
Mean (SD)	16.4 (9.3)	14.3 (8.8)	16.1 (8.7)	13.5 (11.4)	14.1 (8.7)	13.5 (8.7)				
P-value	0.986	0.888	0.182	0.957	0.526	0.843				0.65 (0.420)
**AADQ**										
ACT								Intercept	53.40 (50.50, 56.30)	
N	39	24	21	19	17	17		ACT	4.80 (0.80, 8.82)	
Mean (SD)	57.4 (8.8)	58.7 (7.6)	59.6 (7.7)	59.0 (9.2)	62.1 (8.5)	61.2 (8.3)		Time	0.03 (0.01, 0.05)	
Control								ACT*time	0.01 (-0.02, 0.04)	
No.	32	25	25	21	19	16				

For peer review only

Mean (SD)	52.6 (11.3)	54.3 (11.3)	54.5 (9.6)	58.3 (7.8)	56.5 (7.1)	57.8 (5.9)				
P value	0.049	0.207	0.047	0.560	0.022	0.073				0.34(0.562)
**MANSA**										
ACT								Intercept	4.65 (4.38, 4.91)	
N	39	24	21	19	17	17		ACT	0.44 (0.07, 0.80)	
Mean (SD)	5.0 (0.9)	5.1 (0.8)	5.3 (0.8)	5.1 (0.9)	5.3 (0.6)	5.3 (0.9)		Time	0.00 (0.00, 0.00)	
Control								ACT*time	0.00 (0.00, 0.01)	
N	32	25	25	21	19	16				
Mean (SD)	4.7 (0.7)	4.7 (1.0)	4.5 (0.9)	4.8 (0.9)	4.8 (0.9)	4.5 (0.8)				
P value	0.093	0.118	0.006	0.266	0.038	0.015				5.79(0.016)
**SDSCA-GD**										
ACT								Intercept	4.14 (3.61, 4.67)	
No.	39	24	21	19	17	17		ACT	0.75 (0.02, 1.48)	
Mean (SD)	5.0 (1.6)	4.7 (1.8)	4.5 (1.7)	4.9 (1.6)	5.1 (1.5)	5.1 (1.5)		Time	0.01 (0.00, 0.01)	
Control								ACT*time	0.00 (-0.01, 0.01)	
No.	32	25	25	21	19	16				
Mean (SD)	4.1 (1.9)	3.8 (2.1)	4.4 (1.8)	4.9 (1.4)	4.9 (1.8)	4.7 (1.6)				
P value	0.062	0.104	0.722	0.743	0.784	0.388				0.01(0.924)
**SDSCA- SD**										
ACT								Intercept	3.66 (3.18, 4.15)	
N	39	24	21	19	17	17		ACT	0.21 (-0.46, 0.88)	
Mean (SD)	3.8 (1.8)	4.1 (1.5)	3.6 (1.4)	4.5 (1.2)	4.2 (1.6)	4.1 (1.7)		Time	0.00 (-0.01, 0.00)	
Control								ACT*time	0.00 (0.00, 0.01)	
No.	32	25	25	21	19	16				

For peer review only

Mean (SD)	3.5 (1.6)	3.7 (1.6)	3.7 (1.6)	4.2 (1.5)	3.7 (1.5)	3.6 (1.6)			
P value	0.313	0.415	0.706	0.405	0.425	0.269			1.37 (0.242)
**SDSCA-E**									
ACT							Intercept	2.62 (2.03, 3.22)	
No.	39	24	21	19	17	17	ACT	0.73 (-0.10, 1.55)	
Mean (SD)	3.2 (2.1)	4.2 (1.9)	3.2 (2.0)	3.8 (2.3)	4.1 (2.1)	3.6 (2.2)	Time	0.00 (0.00, 0.01)	
Control							ACT*time	0.00 (-0.01, 0.01)	
N	32	25	25	21	19	16			
Mean (SD)	2.5 (2.0)	2.7 (2.0)	2.5 (1.9)	2.9 (1.8)	3.2 (2.6)	2.9 (1.7)			
P value	0.174	0.010	0.186	0.253	0.245	0.536			0.10 (0.749)
**SDSCA -G**									
ACT							Intercept	5.37 (4.83, 5.91)	
N	39	24	21	19	17	17	ACT	0.84 (0.09, 1.59)	
Mean (SD)	6.1 (1.8)	6.3 (1.2)	6.3 (1.4)	6.6 (0.9)	6.9 (0.3)	6.8 (0.8)	Time	0.01 (0.00, 0.01)	
Control							ACT*time	0.00 (-0.01, 0.01)	
N	32	25	25	21	19	16			
Mean (SD)	5.4 (2.5)	5.5 (2.3)	5.7 (2.2)	6.0 (1.7)	6.0 (1.9)	6.7 (0.8)			
P value	0.423	0.300	0.613	0.265	0.117	0.924			0.23 (0.634)

ACT, acceptance and commitment therapy; SD, standard deviation; CI, confidence interval; HbA1c, glycated haemoglobin; Scale; HFS, Hypoglycaemia Fear Survey (HFS- W-Worry, HFS-B- Behaviour); AADQ, Acceptance Action Diabetes Questionnaire; MANSA, Manchester Short Assessment of Quality of life; SDSCA, Summary of Diabetes Self-Care Activities (SDSCA-GD-general diet, SDSCA-SD-specific diet, SDSCA-E-Exercise, SDSCA-G-Glucose monitoring).

For peer review only

For peer review only - http://bmjopen.bmj.com/site/about/guidelines.xhtml

Supplementary Table 5: ACT exposure (number of sessions attended) and proportion of participants with HbA1c measurement in each timepoint

**ACT exposure**	**Frequency** Intervention group (n =43)	**Frequency** Control group (n=38)
Zero sessions	8	-
One session	3	-
Two sessions	5	-
Three sessions	7	-
Four sessions	1	-
Five sessions	3	-
Six sessions	9	-
Seven sessions	7	-
**HbA1c measurement**		
Week 8, n (%)	27 (62.8)	20 (52.6)
Week 14, n (%)	21 (48.8)	21 (55.3)
6 months, n (%)	15 (34.9)	15 (39.5)
12 months, n (%)	16 (37.2)	17 (44.7)
24 months, (%)	15 (34.9)	11 (28.9)

ACT, acceptance and commitment therapy; HbA1c, glycated Haemoglobin.

For peer review only

CONSORT 2010 checklist of information to include when reporting a randomised trial*

Section/Topic	Item No	Checklist item	Reported on page No
**Title and abstract**			
	1a	Identification as a randomised trial in the title	Title page
	1b	Structured summary of trial design, methods, results, and conclusions (for specific guidance see CONSORT for abstracts)	1
**Introduction**			
Background and objectives	2a	Scientific background and explanation of rationale	2
	2b	Specific objectives or hypotheses	3
**Methods**			
Trial design	3a	Description of trial design (such as parallel, factorial) including allocation ratio	3-4
	3b	Important changes to methods after trial commencement (such as eligibility criteria), with reasons	-
Participants	4a	Eligibility criteria for participants	3
	4b	Settings and locations where the data were collected	4
Interventions	5	The interventions for each group with sufficient details to allow replication, including how and when they were actually administered	4
Outcomes	6a	Completely defined pre-specified primary and secondary outcome measures, including how and when they were assessed	4-5
	6b	Any changes to trial outcomes after the trial commenced, with reasons	-
Sample size	7a	How sample size was determined	3
	7b	When applicable, explanation of any interim analyses and stopping guidelines	-
Randomisation:			
Sequence generation	8a	Method used to generate the random allocation sequence	3
	8b	Type of randomisation; details of any restriction (such as blocking and block size)	3
Allocation concealment mechanism	9	Mechanism used to implement the random allocation sequence (such as sequentially numbered containers), describing any steps taken to conceal the sequence until interventions were assigned	3-4
Implementation	10	Who generated the random allocation sequence, who enrolled participants, and who assigned participants to interventions	3-4
Blinding	11a	If done, who was blinded after assignment to interventions (for example, participants, care providers, those	-

For peer review only

		assessing outcomes) and how	
	11b	If relevant, description of the similarity of interventions	-
Statistical methods	12a	Statistical methods used to compare groups for primary and secondary outcomes	6
	12b	Methods for additional analyses, such as subgroup analyses and adjusted analyses	-
**Results**			
Participant flow (a diagram is strongly recommended)	13a	For each group, the numbers of participants who were randomly assigned, received intended treatment, and were analysed for the primary outcome	7
	13b	For each group, losses and exclusions after randomisation, together with reasons	7&10
Recruitment	14a	Dates defining the periods of recruitment and follow-up	3-4
	14b	Why the trial ended or was stopped	-
Baseline data	15	A table showing baseline demographic and clinical characteristics for each group	8-9
Numbers analysed	16	For each group, number of participants (denominator) included in each analysis and whether the analysis was by original assigned groups	7
Outcomes and estimation	17a	For each primary and secondary outcome, results for each group, and the estimated effect size and its precision (such as 95% confidence interval)	10-13
	17b	For binary outcomes, presentation of both absolute and relative effect sizes is recommended	-
Ancillary analyses	18	Results of any other analyses performed, including subgroup analyses and adjusted analyses, distinguishing pre-specified from exploratory	13
Harms	19	All important harms or unintended effects in each group (for specific guidance see CONSORT for harms)	-
**Discussion**			
Limitations	20	Trial limitations, addressing sources of potential bias, imprecision, and, if relevant, multiplicity of analyses	14
Generalisability	21	Generalisability (external validity, applicability) of the trial findings	14
Interpretation	22	Interpretation consistent with results, balancing benefits and harms, and considering other relevant evidence	13-14
**Other information**			
Registration	23	Registration number and name of trial registry	1
Protocol	24	Where the full trial protocol can be accessed, if available	3
Funding	25	Sources of funding and other support (such as supply of drugs), role of funders	15

*We strongly recommend reading this statement in conjunction with the CONSORT 2010 Explanation and Elaboration for important clarifications on all the items. If relevant, we also recommend reading CONSORT extensions for cluster randomised trials, non-inferiority and equivalence trials, non-pharmacological treatments, herbal interventions, and pragmatic trials. Additional extensions are forthcoming: for those and for up to date references relevant to this checklist, see www.consort-statement.org.

For peer review only - http://bmjopen.bmj.com/site/about/guidelines.xhtml

BMJ Open

Impact of an Acceptance and Commitment Therapy programme on HbA1c, self-management and psychosocial factors in adults with type 1 diabetes and elevated HbA1c levels: a randomised controlled trial

Journal:	*BMJ Open*
Manuscript ID	bmjopen-2023-072061.R2
Article Type:	Original research
Date Submitted by the Author:	05-Dec-2023
Complete List of Authors:	Wijk, Ingrid ; Sophiahemmet University, Department of Health Promoting Science<br>Amsberg, Susanne; Marie Cederschiöld högskola - Campus Ersta, Department of Health Care Sciences<br>Johansson, Unn-Britt; Sophiahemmet University, Department of Health Promoting Science; Karolinska Institutet, Department of Clinical Sciences and Education, Södersjukhuset<br>Livheim, Fredrik; Karolinska Institutet, Department of Clinical Neuroscience<br>Toft, Eva; Karolinska Institutet, Department of Medicine, Huddinge; Karolinska Institutet, Department of Medicine, Ersta Hospital<br>Anderbro, Therese; Karolinska Institutet, Department of Clinical Sciences; Stockholm University, Department of Psychology
**Primary Subject Heading**:	Diabetes and endocrinology
Secondary Subject Heading:	Mental health
Keywords:	DIABETES & ENDOCRINOLOGY, Depression & mood disorders < PSYCHIATRY, PREVENTIVE MEDICINE

SCHOLARONE™ Manuscripts

I, the Submitting Author has the right to grant and does grant on behalf of all authors of the Work (as defined in the below author licence), an exclusive licence and/or a non-exclusive licence for contributions from authors who are: i) UK Crown employees; ii) where BMJ has agreed a CC-BY licence shall apply, and/or iii) in accordance with the terms applicable for US Federal Government officers or employees acting as part of their official duties; on a worldwide, perpetual, irrevocable, royalty-free basis to BMJ Publishing Group Ltd ("BMJ") its licensees and where the relevant Journal is co-owned by BMJ to the co-owners of the Journal, to publish the Work in this journal and any other BMJ products and to exploit all rights, as set out in our licence.

The Submitting Author accepts and understands that any supply made under these terms is made by BMJ to the Submitting Author unless you are acting as an employee on behalf of your employer or a postgraduate student of an affiliated institution which is paying any applicable article publishing charge ("APC") for Open Access articles. Where the Submitting Author wishes to make the Work available on an Open Access basis (and intends to pay the relevant APC), the terms of reuse of such Open Access shall be governed by a Creative Commons licence – details of these licences and which Creative Commons licence will apply to this Work are set out in our licence referred to above.

Other than as permitted in any relevant BMJ Author's Self Archiving Policies, I confirm this Work has not been accepted for publication elsewhere, is not being considered for publication elsewhere and does not duplicate material already published. I confirm all authors consent to publication of this Work and authorise the granting of this licence.

For peer review only

Impact of an Acceptance and Commitment Therapy programme on HbA1c, self-management and psychosocial factors in adults with type 1 diabetes and elevated HbA1c levels: a randomised controlled trial

Ingrid Wijk[1*], Susanne Amsberg[2], Unn-Britt Johansson[1,3], Fredrik Livheim[4], Eva Toft[5,6], Therese Anderbro[7,8]

[1]Department of Health Promoting Science, Sophiahemmet University, Stockholm, Sweden

[2]Department of Health Care Sciences, Marie Cederschiöld University, Stockholm, Sweden

[3]Department of Clinical Sciences and Education, Södersjukhuset, Karolinska Institutet, Stockholm, Sweden

[4]Department of Clinical Neuroscience, Karolinska Institutet, Stockholm, Sweden

[5]Department of Medicine, Huddinge, Karolinska Institutet, Stockholm, Sweden

[6]Department of Medicine, Karolinska Institutet, Ersta Hospital, Stockholm, Sweden

[7]Department of Clinical Sciences, Danderyd Hospital, Karolinska Institutet, Stockholm, Sweden

[8]Department of Psychology, Stockholm University, Stockholm, Sweden

*Corresponding author:

E-mail address: ingrid.wijk@shh.se (I. Wijk).

Sophiahemmet University

P O Box 5605

SE-114 86 Stockholm, Sweden

For peer review only

ABSTRACT

Objective: To evaluate the impact of an Acceptance and Commitment Therapy (ACT) programme, tailored for people living with type 1 diabetes, on HbA1c, self-management and psychosocial factors among individuals with HbA1c >60 mmol/mol compared with treatment as usual (TAU).

Setting: An endocrinologic clinic in Sweden.

Participants: In this randomised controlled trial, 81 individuals with type 1 diabetes, aged 18-70 years with HbA1c >60 mmol/mol, were randomly assigned to either an ACT group intervention or TAU. Exclusion criteria were: not able to speak Swedish, untreated or severe psychiatric disease, cortisone treatment, untreated thyroid disease, and newly started insulin pump therapy. At the two-year follow-up, HbA1c was measured in 26 individuals.

Intervention: The ACT programme comprised seven 2-hour sessions held over 14 weeks and focused on acceptance of stressful thoughts and emotions, and to promote value-based committed action.

Outcomes: The primary outcome was HbA1c, and the secondary outcomes were measures of depression, anxiety, general stress, fear of hypoglycaemia, diabetes distress, self-care activities, psychological flexibility (general and related to diabetes) and quality of life. The primary endpoint was HbA1c two years after the intervention programme. Linear mixed models were used to test for an interaction effect between measurement time and group.

Results: Likelihood ratio test of nested models demonstrated no statistically significant interaction effect (chi-square=0.49, p=0.485) between measurement time and group regarding HbA1c. However, a statistically significant interaction effect (likelihood ratio test chi-square=12.63, p<0.001) was observed with improved scores on The Acceptance and Action Questionnaire in the intervention group after one and two years.

Conclusions: No statistically significant difference was found between the groups regarding the primary outcome measure, HbA1c. However, the ACT programme showed a persistent beneficial impact on psychological flexibility in the intervention group. The dropout rate was higher than expected which may indicate a challenge in this type of study.

Trial registration: Trial registration number NCT02914496.

Strengths and limitations of this study:

- This study was developed using an interdisciplinary approach, with a randomised control trial design.
- The intervention is theory-based, with roots in behavioural research, and is tailored for living with diabetes.
- This research addresses psychological aspects of living with type 1 diabetes which has been requested by the diabetes community and in international guidelines.

For peer review only - http://bmjopen.bmj.com/site/about/guidelines.xhtml

- The drop-out rate in this study affects the possibility to draw conclusions from the findings.

BACKGROUND

Type 1 diabetes is an autoimmune mediated disease (1) with an increasing global incidence in young people under 20 years of age (2). Complications of type 1 diabetes involve life-threating conditions such as hypoglycaemia and ketoacidosis as well as micro- and macro vascular diseases (3). National and international guidelines recommend treatment targets for glycaemic level, individually adjusted, to prevent development of complications (4, 5). However, only a minority reach the treatment targets (6-8).

The pancreatic beta cell destruction in type 1 diabetes brings a life-long dependency on supplying exogenous insulin (3) and along with that a self-management posing high demands (9). Glucose monitoring, insulin adjustment and managing hypo- and hyperglycaemia are examples of self-care activities involved (10). In addition to the amount of ingested carbohydrates, several factors such as physical activity, hormones, stress and temperature affect the glycaemic level (10). Consequently, self-management requires knowledge and skills (11) and involves frequent decision-making (12). Emotional stress related to the management of diabetes, diabetes distress, persists over time (13) and is present among 20%–30% of people with type 1 diabetes (14). Furthermore, the prevalence of depression is up to three-fold higher among individuals living with type 1 diabetes (15). Moreover, depression in individuals with diabetes is linked with an increased risk of mortality (16). Several studies have found that psychological health problems and elevated HbA1c levels are associated (17-19). Consequently, there has been a call to address the psychological aspects of living with diabetes (20, 21).

Cognitive Behaviour Therapy (CBT) is focused on restructuring maladaptive thoughts to create more functional cognition and behaviours (22). CBT has shown positive effects on depression in a general population (23) as well as among individuals with diabetes (24). In a randomised controlled trial (RCT) by Amsberg et al., a CBT intervention improved HbA1c and reduced levels of depression and diabetes distress (25).

Acceptance and Commitment Therapy (ACT) expands upon CBT. The approach in ACT suggests that the presence of uncomfortable thoughts and feelings should be accepted and not supressed or modified owing to the fact that human life is difficult (26). Despite the existence of uncomfortable thoughts, functional behaviours could pave the way to a life in accordance with a person's values and goals. Being present and aware, and at the same time developing functional behaviours adapted in the current context, is referred to as psychological flexibility (27). Moreover, psychological flexibility, which ACT aims to increase (28), is suggested to be a key prerequisite to psychological health (29). ACT is based on six flexibility processes: acceptance - allowance of difficult thoughts and feelings, defusion- not getting fused with thoughts and feelings, self-as-context – observing thoughts and feelings, attention to the present moment – being mindful of the current internal and external environment, values – stating personal values that may provide direction in life and commitment – committed action - creating behaviours that corresponds to personal values (27). Previous studies have supported treatment effects of ACT for different mental and somatic health problems (30). With its focus on acceptance of

For peer review only - http://bmjopen.bmj.com/site/about/guidelines.xhtml

discomfort, ACT has also been argued to be especially suitable for long-term conditions where distress can be a realistic factor (31). Furthermore, a recent study found that greater psychological flexibility in adults with type 1 diabetes was associated with less distress and more beneficial HbA1c (32). A meta-analysis concluded that ACT interventions may improve the ability of self-care, psychological flexibility and HbA1c in type 2 diabetes (33). Within the scope of type 1 diabetes, few ACT interventions for adults have been evaluated. A feasibility study of a single-arm trial involving individuals with type 1 diabetes and comorbid eating disorder indicated positive effects on psychological flexibility and diabetes distress, in addition to being feasible and improving eating disorder symptoms. Furthermore, an RCT feasibility study of an ACT-based intervention provided in webformat included individuals with type 1 and type 2 diabetes. A reduction in diabetes distress was seen in the intervention group. However, the dropout rate was 57% during treatment (34).

In summary, type 1 diabetes is a severe disease with an increased prevalence of impaired psychological health with treatment targets reached by only a minority. Previous research regarding ACT and diabetes has mainly focused on type 2 diabetes. Our hypothesis was that an ACT programme would lead to improvements in glycaemic and psychosocial outcomes in an adult population with type 1 diabetes and prolonged elevation of HbA1c. Thus, we conducted an RCT aiming to evaluate the impact of an ACT programme, tailored for people living with type 1 diabetes, on HbA1c, self-management and psychosocial factors.

METHODS

Study design and participants

This study was a longitudinal two-arm RCT conducted at an endocrinologic clinic in Sweden. The intervention group participated in an ACT programme and the control group received treatment as usual (TAU) only. The endocrinology clinic population comprised adults (> 18 years) with type 1 diabetes and included urban and suburban residents. A study protocol has been published where a more detailed description of the method and the intervention is provided (35).

Eligible participants were identified by extracting data from the Swedish National Diabetes Register (NDR). Individuals who met the inclusion criteria: type 1 diabetes with a duration of at least 2 years, aged 18–70 years and HbA1c > 60 mmol/mol on two occasions during preceding year, were assessed in a random order (generated by computer). Sequentially, patient records were reviewed for information on exclusion criteria. The following criteria were applied: not able to speak Swedish, untreated, or severe ongoing psychiatric disease, cortisone treatment, untreated thyroid disease, and insulin pump therapy treatment started since < 3 months.

Recruitment

A power analysis before recruitment suggested that at least 56 individuals with type 1 diabetes were needed to reach 80% power for detecting a clinically relevant reduction in HbA1c of 6 mmol/mol (with assumed standard deviation [SD] of 9) at a 5% significance level. To allow for dropouts of approximately 24% (25) the aim was to recruit 70 persons with type 1 diabetes.

From 2016 to 2019 individuals with HbA1c >60 mmol/mol (reference value 42 mmol/mol) were assessed for eligibility (n=622) in five rounds. Study information and an invitation to participate were sent by post

For peer review only - http://bmjopen.bmj.com/site/about/guidelines.xhtml

to eligible individuals who met the criteria for inclusion (n=343). Two weeks after the material was sent, a research nurse assistant telephoned the invited individuals to offer more information about the study, if desired. Eighty-one people submitted written informed consent and were included in the study. Consequently, the participation rate was 23.6% (81 of 343 who received an invitation). Sociodemographic details and information on treatment were collected at baseline.

Randomisation

After the baseline measurements, participants were randomly allocated into either the intervention or control group (Figure 1). A person not involved with the project conducted randomisation by drawing sealed lots. Stratification was carried out to achieve an equal distribution by sex in the groups. The research assistant nurse informed participants of the group assignment by e-mail and provided further instructions three weeks before the start of the intervention programme. Blinding the ACT-instructors or the research assistant nurse to the group allocations during the study period was not possible. However, the ACT-instructors never met the control group; additionally, the research nurse assistant was not involved in the intervention programme and did not have ACT skills.

Intervention

This study was developed by a multidisciplinary team consisting of psychologists, nurses specialised in diabetes and an endocrinologist**.** The ACT programme consisted of seven two-hour sessions that were held every other week. A text message was sent the day before each session as a reminder. The size of the ACT groups ranged from six to nine participants. Furthermore, the programme was manual-based and developed according to a general ACT programme (36). The manual was tailored by a psychologist specialised in diabetes and a diabetes nurse to direct the focus to living with type 1 diabetes; both were trained ACT instructors. The sessions involved activities connected to the six core components of ACT: being present, acceptance, defusion, self as context, values, and commitment (27). Recurring activities throughout the programme were group discussions, psychoeducation, exercises, mindfulness practice, role play and value awareness. There was also homework between sessions.

The intervention group followed the ACT programme alongside TAU and the control group received TAU. TAU comprised a minimum of two visits (one appointment with an endocrinologist and one with a nurse specialised in diabetes) at the clinic each year. The needs of the patient governed the number of visits and telephone contacts.

Assessments

Time points for measurement were at baseline, weeks 8 and 14 during the intervention and repeated measurements at 6 months, one year and two years after the intervention. Web-based questionnaires were sent to participants by e-mail and reminders were sent one and two weeks after the first e-mail, if needed. The questionnaires and reminders were sent via a web-based tool. Three weeks before each follow-up assessment, a letter with information regarding the coming measurements and dates for HbA1c measurement was sent via post and via e-mail. Reminders of the upcoming dates were sent a couple of days before HbA1c measurement, which took place close to the clinic but not in the hospital area. If both the questionnaires and HbA1c measurement were completed, a gift card of 100 Swedish crowns was sent to the participant after each time point of measurement.

Primary outcome measure

For peer review only - http://bmjopen.bmj.com/site/about/guidelines.xhtml

The primary outcome was glycated haemoglobin, HbA1c. The measurement reflects the average glycaemic level during the previous two to three months (37). The treatment goal should be individually set; however, a general treatment target for adults is < 53 mmol/mol (38). We used capillary blood samples and equipment to conduct point-of-care immunoassay (DCA Vantage Analyzer, Siemens) which has shown sufficient correlation with the reference method, high-performance liquid chromatography (39). The method of analysis is calibrated against IFCC standard (International Federation of Clinical Chemists). The same equipment was used at each measurement time point and a checklist for calibration and maintenance was followed in accordance with the manufacturer's recommendations (40).

Secondary outcome measures

Depression Anxiety Stress Scales

Depression, anxiety and stress, were assessed with The Depression Anxiety, Stress Scale (DASS21) (41). The questionnaire consists of 21 items divided into three subscales. A 4-point Likert scale, starting with *didn't apply to me at all* and ending with *applied to me very much or most of the time,* is used for rating (41). Psychometric properties have been evaluated in a Swedish population (42). Cronbach's alpha in the subscales in the current study ranged from 0.75 to 0.92.

Hypoglycaemia Fear Survey

Fear of hypoglycaemia was assessed by using The Hypoglycaemia Fear Survey (HFS) (41, 43). The scale with 23 items in total involves two subscales: worry and behaviour. A 5-point Likert Scale is used for scoring (0=never, 4=always). A higher score indicates a more explicit fear of hypoglycaemia (43). The Swedish version has been evaluated regarding psychometric properties (44). Cronbach's alpha for the total scale used in the current study was 0.86.

Problem Areas in Diabetes Scale

The Problem Areas in Diabetes Scale (PAID) is a measure for assessing emotional distress related to diabetes (45). The questionnaire consists of 20 items and is scored on a 5-point Likert scale (0=not a problem to 4= serious problem) (45). Greater distress is indicated with higher scores (total score 0-100) where 40 is the cut-off for significant distress (46, 47). The reliability and validity of the Swedish version scale have been evaluated (48). Cronbach`s alpha in the current study was 0.91.

Summary of Diabetes Self-Care Activities

The frequency of self-care activities over the past seven days was measured with the Summary of Diabetes Self-Care Activities (SDSCA) (49). The questionnaire includes eleven items in different areas of self-care activity. The scoring system ranges from 0 to 7 and refers to the number of weekdays. The areas are separately scored and are not summed in a total score. High scores indicate high self-care performance (49). A Swedish version, translated by Amsberg et al., was used which also demonstrated

For peer review only - http://bmjopen.bmj.com/site/about/guidelines.xhtml

sufficient internal consistency for the subscales (Cronbach's alpha 0.80 – 0.86) in a similar sample (25). In the current study, Cronbach's alpha for the subscales diet, exercise and glucose monitoring ranged from 0.80 to 0.90.

Acceptance and Action Questionnaire

The original Acceptance and Action Questionnaire (AAQ) (50) was developed into a revised version, the AAQ-II (51) to measure the degree of psychological flexibility. The revised scale AAQ-II has been translated into Swedish and evaluated regarding psychometric features. This version was used in our study. There are six items in the Swedish version that are rated on a 7-point Likert scale (1= never true, 7 =always true). A lower score reflects greater psychological flexibility (52). Cronbach`s alpha in the current study was 0.88.

Acceptance Action Diabetes Questionnaire

Psychological flexibility related to diabetes was assessed using the Acceptance Action Diabetes Questionnaire (AADQ). The questionnaire is derived from the AAQ and was developed by Gregg et al. (53). The original AADQ comprises eleven items and is rated on a 7-point Likert scale (1= never true, 7= always true). A higher score indicates a greater psychological flexibility (53) in contrast to the AAQ which is scored in the opposite direction (52). The scale was translated into Swedish, and the psychometric properties has been evaluated. The manuscript has been accepted but is not yet published. Cronbach`s alpha in the current study was 0.77.

Manchester Short Assessment of Quality of life

The Manchester Short Assessment of Quality of life (MANSA) is used to assess quality of life (QoL). The questionnaire was developed by Priebe et al. (54) and was constructed with 16 items (12 items related to general QoL and 4 are objective items of QoL). The rating score ranges from 1 to 7 (1= could not be worse, 7 = could not be better). The response categories in the objective items are dichotomous (yes, no). A Swedish version has been evaluated regarding the psychometric properties (55). The Cronbach`s alpha in the current study was 0.85.

Statistical methods

Baseline characteristics were described as mean (SD) for continuous variables and absolute and relative frequencies for categorical variables. Tests for differences in baseline characteristics between completers and non-completers and intervention (control group, intervention group) were performed using the Kruskal-Wallis test (continuous variables) and chi-squared test (categorical variables). Mixed effect models were fitted to test for changes in HbA1c levels and questionnaire scores across follow-up. Each model included the time point (baseline, week 8, week 14, week 38, week 62) and intervention (control group, intervention group) as fixed effects and a random intercept for participants. Interaction effects between the time point and intervention were explored. Test of fixed effects were done using a likelihood ratio test in nested models using the chi-square distribution. We implemented a compound

For peer review only - http://bmjopen.bmj.com/site/about/guidelines.xhtml

symmetry structure within our mixed-effects model. Graphical assessment of residuals plotted against predicted values were conducted to validate the model's assumption.

All statistical analyses were performed using R version 3.6.2 (56). IBM SPSS 27 was used for graphs (IBM Corp., Armonk, NY, USA). Other statistical analyses that were planned and presented in the study protocol could not be conducted or were not suitable owing to a high number of missing values and lack of statistical significance in outcome. The intention-to-treat principle was applied in the primary analysis. In addition, a per-protocol analysis was conducted in a further examination of the results.

Fidelity check of the intervention

For quality assurance, a fidelity check was performed by videotaping one session held by the licensed psychologist and the registered nurse. The videotape was reviewed by DE, an experienced trainer who has been training instructors in the ACT-group intervention that was used. In addition, participants in two groups and the group leaders estimated the adherence to the treatment intervention and manual by completing an adherence evaluation after sessions three and seven. The aim was to measure how adherent each participant had been to the treatment interventions in the sessions and how well the group leaders adhered to the treatment manual. The group leaders also evaluated their adherence to the manual. Finally, participants' and group leaders' evaluations were compared. Participants and group leaders rated each intervention in the session on a scale from 0 to 10 where 0 = "this has not been discussed or brought up", to 10 = "This was covered thoroughly". The estimates are shown in Supplementary Table 1 and Supplementary Table 2 (Supplementary Material).

Insert figure 1

PATIENT AND PUBLIC INVOLVEMENT

The RCT was preceded by an informal pre-trial among eight volunteers with type 1 diabetes to test the design and content of the ACT intervention. On the basis of the feedback of participants, an adaptation of the programme was made with shorter but more sessions. The participants in the RCT will receive a message when the results are published and where to find them. There will also be a link provided to a webpage with a summary of the results in plain Swedish.

RESULTS

Study participants

The mean age for all study participants in the sample was 40.2 (SD 16.6) years and 63% were women. The mean duration of type 1 diabetes was 23.4 (SD 14.4) years, the mean HbA1c was 73.5 (SD 13) mmol/mol and 74.7% of participants have had retinopathy on some occasion. Approximately 88% of participants used sensor-based glucose monitoring and 35.8% wore an insulin pump. In Table 1 the baseline characteristics are described for the total sample and the allocated trial groups. No statistically

For peer review only - http://bmjopen.bmj.com/site/about/guidelines.xhtml

significant differences between group characteristics were found at baseline. Baseline scoring of the secondary outcomes and comparisons between the groups are shown in Supplementary Table 3 and Supplementary Table 4 (Supplementary Material). No statistically significant differences were found between the groups in baseline scoring (Bonferroni correction applied). Significantly elevated diabetes distress was present among 51% of the study sample, (PAID score >40). Mean scores in the normal range were noted for stress and anxiety. The mean score in the depression subscale of 12.14 (SD 10.85) indicated mild depression, and 39% of participants had scores corresponding to moderate to severe depression.

For peer review only

Table 1: Baseline characteristics of trial groups with comparisons between complete (participants completing the two-year follow-up) and drop-out (participants lost to follow-up or who discontinued the study at the two-year follow-up) groups.

Characteristics	All participants	Intervention group	Control group	P value	Complete	Dropout	P value
**N**	81	43	38		26	55	
**Males**, n (%)	30 (37)	15 (34.9)	15 (39.5)	0.844	8 (30.8)	22 (40)	0.578
**Age** (years) (SD, range)	40.2 (16.6, 19-69)	42.2 (16.5, 19-69)	37.9 (16.6, 20-68)	0.368	38.6 (16.4)	40.9 (16.8)	0.754
**Diabetes duration** (years) (SD, range)	23.4 (14.4, 3-61)	23.6 (14.0, 3-61)	23.2 (15, 5-61)	0.730	20.2 (12.5)	24.9 (15.1)	0.156
**Family/Cohabitation**							
With a partner, n (%)	36 (44.4)	17 (39.5)	19 (50)		11 (42.3)	25 (45,5)	
With parents, n (%)	14 (17.3)	7 (16.3)	7 (18.4)		3 (11.5)	11 (20.0)	
With other, n (%)	5 (6.2)	2 (4.7)	3 (7.9)		0 (0.0)	5 (9.1)	
Alone, n (%)	22 (27.2)	13 (30.2)	9 (23.7)		11 (42.3)	11 (20.0)	
Apart, n (%)	4 (4.9)	4 (9.3)	0 (0.0)	0.314	1 (3.8)	3 (5.5)	0.167
**Education (highest level)**							
Elementary school, n (%)	4 (4.9)	3 (7.0)	1 (2.6)		0 (0.0)	4 (7.3)	
Upper secondary school, n (%)	30 (37.0)	14 (32.6)	16 (42.1)		8 (30.8)	22 (40.0)	
University (courses or education) n (%)	41 (50.6)	24 (55.8)	17 (44.7)		16 (61.5)	25 (45.5)	
Other, n (%)	6 (7.4)	2 (4.7)	4 (10.5)	0.441	2 (7.7)	4 (7.3)	0.361
**Employment**							
Retired, n (%)	6 (7.4)	3 (7.0)	3 (7.9)		1 (3.8)	5 (9.1)	
Early retirement, n (%)	5 (6.2)	3 (7.0)	2 (5.3)		3 (11.5)	2 (3.6)	
Student, n (%)	11 (13.6)	5 (11.6)	6 (15.8)		3 (11.5)	8 (14.5)	
Not currently working for medical reasons, n (%)	2 (2.5)	2 (4.7)	0 (0.0)		0 (0.0)	2 (3.6)	
Unemployed, n (%)	3 (3.7)	1 (2.3)	2 (5.3)		1 (3.8)	2 (3.6)	

For peer review only - http://bmjopen.bmj.com/site/about/guidelines.xhtml

Employed, n (%)	44 (54.3)	21 (48.8)	23 (60.5)		15 (57.7)	29 (52.7)	
Manage own company, n (%)	10 (12.3)	8 (18.6)	2 (5.3)	0.420	3 (11.5)	7 (12.7)	0.730
**CGM or FGM**, n (%)	71 (87.7)	37 (86)	34 (89.5)	0.897	24 (92.3)	47 (85.5)	0.608
**CSII**, n (%)	29 (35.8)	15 (34.9)	14 (36.8)	1.000	12 (46.2)	17 (30.9)	0.277
**BMI** (SD, range)	26.1 (4.7, 17.3-39)	25.5 (4.5, 17.3-36.5)	26.8 (4.7, 19.6-39)	0.250	26.6 (3.4)	25.8 (5.1)	0.210
**Baseline HbA1c** (mmol/mol) (SD, range)	73.5 (13, 60-125)	71.4 (10.1, 60-118)	75.9 (15.4, 61-125)	0.315	69.2 (7.2)	75.5 (14.6)	0.029
**Retinopathy,** n (%) *	59 (74.7)	30 (73.2)	29 (76.3)	0.950	17 (65.4)	42 (79.2)	0.291
**Neuropathy**, n (%) *	23 (29.1)	13 (31.7)	10 (26.3)	0.780	9 (34.6)	14 (26.4)	0.624
**Nephropathy,** n (%) *							
Micro-albuminuria	4 (5.2)	2 (5.0)	2 (5.4)		2 (7.7)	2 (3.9)	
Macro-albuminuria	2 (2.6)	1 (2.5)	1 (2.7)		0 (0.0)	2 (3.9)	
No albuminuria	71 (92.2)	37 (92.5)	34 (91.9)	0.995	24 (92.3)	47 (92.2)	0.474
**Cerebrovascular disease**, n (%) *	1 (1.3)	1 (2.4)	0 (0.0)	1.000	0 (0)	1 (1.9)	1.000
**Coronary artery disease,** n (%) *	3 (3.8)	1 (2.4)	2 (5.3)	0.946	1 (3.8)	2 (3.8)	1.000

*Information missing for some individuals (nephropathy:4, neuropathy:2, retinopathy:2, cerebrovascular disease:4, coronary artery disease:2)

SD, standard deviation; BMI, body mass index; HbA1c, glycated haemoglobin; CGM, continuous glucose monitoring; FGM, flash glucose monitoring; CSII, continuous subcutaneous insulin infusion .

For peer review only - http://bmjopen.bmj.com/site/about/guidelines.xhtml

Dropouts and adherence to the programme

Eight participants did not start the intervention programme and wished to withdraw from the study. Reasons for their decision were as follows: internship in another city (n=1), going abroad (n=1), lack of time (n=1), family reasons (n=1), and having to work (n=1). In three cases, no reason was provided. Reasons for interrupting the programme were as follows: disliked the program (n=2), medical/health related reasons (n= 3), evening time not suitable (n = 1), family reasons (n= 1), and change in working schedule (n=1). One person in the intervention group passed away before the two-year follow-up. In the control group, the reasons reported for discontinuation of the study after randomisation until the last timepoint for measurement (two years) were as follows: medical reasons (n= 1), family reasons (n=1), no longer interested (n=2), lack of time (n=1) and moved to another city (n=2). Parts of the one-year and the two-year follow-up were conducted during the COVID-19 pandemic during 2020-2021. Three participants actively declined the invitation for follow-up measurement because of the pandemic (two who had symptoms and one owing to the risk of infection). However, one HbA1c measurement could be registered from the Endocrinology clinic in the same week. Most reasons for discontinuation/loss to follow-up were unknown.

Supplementary Table 5 (Supplementary Material) presents the exposure to ACT. Thirty-five participants attended at least one session (81.4%) and 20 participants (46.5%) attended four to seven sessions of the programme. The attendance for measurement of HbA1c is also shown in Supplementary Table 5. In week 8 after session 4, 62.8% of participants in the intervention group and 52.6% controls completed HbA1c measurement. The dropout rate was approximately equal in the trial arms during the follow-up period. Furthermore, no significant differences in baseline characteristics were detected among participants remaining in the study after two years and those who were lost to follow-up (Bonferroni correction applied) (Table 1).

Primary outcome

The mean HbA1c in the intervention group decreased over two years from 71.4 mmol/mol to 59.6 mmol/mol. However, the likelihood ratio test of nested models demonstrated no statistically significant interaction effect (chi-square:0.49, p:0.485) between measurement time point and group affiliation regarding HbA1c, as shown in Supplementary Table 3 (Supplementary Material). The observed mean HbA1c over time is illustrated in Figure 2.

Insert Figure 2.

Secondary outcomes

A significant interaction effect (likelihood ratio test chi-square:12.63, p<0.001) between time and group allocation was found in AAQ-II scores measuring psychological flexibility (Supplementary Table 3,

For peer review only - http://bmjopen.bmj.com/site/about/guidelines.xhtml

Supplementary Material). This finding indicates a trend of improvement in the ACT group over time. The observed mean AAQ-II score over two years is illustrated in Figure 3.

Insert Figure 3

No significant interaction effects between time and group allocation were demonstrated in scores of the PAID, DASS21 (Supplementary Table 3, Supplementary Material), HFS, AADQ, MANSA or SDSCA (Supplementary Table 4, Supplementary Material).

Per protocol

Per protocol analyses were conducted; the results were consistent with findings in the intention-to-treat analyses.

DISCUSSION

This study evaluated the impact of an ACT programme tailored for individuals living with type 1 diabetes. The observed mean values decreased to a level below 60 mmol/mol in the intervention group during the study period. However, no statistically significant effect on HbA1c was demonstrated two years after the ACT program. The AAQ-II scores decreased over time and the improvement in psychological flexibility was statistically significant after one and two years. The results regarding HbA1c correspond to those of a meta-analysis involving overall psychological interventions in type 1 diabetes (57) and a meta-analysis of CBT interventions for diabetes related distress in type 1 and type 2 diabetes (58). Some CBT intervention studies solely focusing on type 1 diabetes have shown similar findings (59, 60). At the same time, the meta-analysis of Winkley et. al (2020) and Yang et al (2020) concluded that CBT has the potential to be effective for HbA1c reduction in type 1 diabetes (57, 61) which was clearly demonstrated in the study of Amsberg et .al (2008). Regarding ACT intervention in diabetes the findings in our study stand in contrast to those of previous studies. ACT interventions in type 2 diabetes have shown promising results (33). A recent study of an ACT intervention for adolescents with type 1 diabetes showed a significant beneficial effect on HbA1c (62). To our knowledge, this is one of the first RCTs to evaluate the effect of a group-based face-to-face ACT intervention on HbA1c in an adult population. We used a longitudinal design with a long-term follow-up period of two years which can enlighten the mechanism of change over time. The study protocol was developed according to Standard Protocol Items: Recommendations for Interventional Trials (SPIRIT) 2013 Statement (63) and was previously published and open for transparency and replication. We used instruments that have been evaluated for psychometric properties (with one exception, the translated version of the SDSCA), and the same device for HbA1c measurements.

Interestingly, a persistent beneficial effect was found in psychological flexibility during the follow-up period. The effect was seen one year after the intervention but not before that time. This finding may indicate that psychological flexibility takes time to develop; this was also discussed in a previous study

For peer review only - http://bmjopen.bmj.com/site/about/guidelines.xhtml

(64). We were unable to conduct mediation analysis regarding the role of psychological flexibility in self-management and HbA1c improvement owing to the absence of statistical significance in the outcome variables. However, a development process that requires time would hypothetically generate improvement in HbA1c even later in the follow-up period. Notably, the statistically significant change in psychological flexibility was only observed in the generic AAQ-II and not in the disease-specific AADQ. The AAQ-II is the most frequently used instrument in research for measuring psychological flexibility, but it has also been criticised for being a measure for psychological distress rather than of psychological flexibility (65).

Reflections have been made regarding the connection between the content of the psychological intervention and diabetes self-management in explaining outcomes in similar studies (57, 62). Gregg et al. (2007) evaluated the impact of an educational intervention with and without ACT components in type 2 diabetes (53). The current study did not focus on self-management and did not involve elements of diabetes education, which may have been a disadvantage. Another observed aspect that differentiates the current study from other group-based studies showing HbA1c improvement is the individualised perspective. The intervention in the study by Amsberg et al. (2008) included individual meetings (25), and Alho et al. (2022) conducted interviews regarding expectations prior to the intervention (62). Moreover, participants called for more individualisation in a feasibility trial of a web-based ACT programme (34). An example of a beneficial individualised element in psychological intervention trials is a study by Malin et al. (2020). A brief motivational interview was introduced after the initial session which increased adherence and decreased the drop-out rate in a CBT intervention of chronic pain and cancer (66).

This study has several limitations, among which the main limitation is the dropout rate. Eight individuals (18.6%) allocated to the ACT group did not start the intervention which is in line with a meta-analysis of dropout rates in CBT trials (67). However, the dropout rate during the ACT programme period in the current study far exceeded the aggregated dropout rate in the study by Fernandez et. al (2015) (67). In the study by Bendig et al. (2021) (34) that involved a web-based ACT programme, the dropout rate was 57% in the intervention group during the study period which is more similar to our findings. Nevertheless, there are no indications of different dropout rates between trials based on ACT or other psychological interventions (68). In addition, the Covid-19 pandemic may have affected the attendance for measurement of HbA1c during parts of the one-year and two-years follow-up periods. There were no differences in baseline characteristics between participants who remained at the two-year follow-up and those who were lost to follow-up at the same measurement time point. Without a Bonferroni correction, the HbA1c demonstrated higher values in the group lost to follow-up which would imply a selection bias. Furthermore, the consequence of the high attrition is an underpowered analysis, which may entail an elevated risk of type II error. Another deficiency in the study is the lack of a comprehensive process evaluation such as that Craig et al. (2008) described in the Medical Research Council guidelines for complex interventions (69). On the other hand, we have gained valuable knowledge regarding the context and implementation of ACT groups for adults with type 1 diabetes that lays a foundation for further development. This study indicates that the format of providing tools based on ACT may not be suitable for the targeted study population. The mean age of the participants suggests a life context with demands from family and work life. In addition, previous research has showed that high HbA1c and executive dysfunctions are associated, at least in young adulthood (70). It is possible that comprehensive programmes for behaviour change which requirements of commitment may be

For peer review only - http://bmjopen.bmj.com/site/about/guidelines.xhtml

difficult to fit in a busy life. Brief ACT interventions have demonstrated promising results in different disciplines (71). It is also noteworthy that diabetes distress may be a significant issue for persons with HbA1c within target range (72). Thus, interventions targeting diabetes distress, with elements of brief ACT strategies incorporated in diabetes health care may be beneficial to explore.

Considering that this was a single-center study with significant attrition problems, the external validity is weakened. The study is ongoing with the last measurement timepoint at five years after the intervention. The decision to proceed with follow-up measurements despite the high attrition was made owing to the effects of psychological flexibility and tendencies toward a decreasing HbA1c.

In conclusion, this study showed no statistically significant difference between the groups regarding the primary outcome measure, HbA1c. However, the ACT programme showed a persistent beneficial impact on psychological flexibility in the intervention group.

Author contributions

Study conceptualisation and design: TA, SA, FL, ET. Acquisition of data: IW, ET. Analysis and interpretation of data: IW in collaboration with an external statistician and TA, U-BJ, SA. Manuscript draft: IW. Revision and approval of final draft: TA, U-BJ, SA, ET, FL.

Ethics approval

This study was approved by the Swedish Ethical Review Authority (2016/14-31/1).

Funding

The study was supported by Lindhés advokatbyrå AB (grant nr: LA2016-0429), the Annie and Fritz Tjus donation fund (grant nr: N/A), the Sophiahemmet Foundation (grant nr: N/A) and the Swedish Diabetes Foundation (grant nr: DIA2018-360).

Competing interests

FL receives royalties from his books written on ACT and income from training professionals in ACT.

Data sharing statement

The data may be available upon reasonable request.

Acknowledgement

We would like to thank all participants in the study and RN Neide Johansson for supporting the recruitment and data collection. Furthermore, we would like to acknowledge Fredrik Wiklund (Statisticon AB) for statistical support and Psychologist Daniel Ek (DE) for the fidelity check of the videotaped material. We also thank Edanz for language editing.

REFERENCES

1. WHO. Classification of Diabetes Mellitus. Geneva: WHO; 2019.

For peer review only - http://bmjopen.bmj.com/site/about/guidelines.xhtml

2. Patterson CC, Karuranga S, Salpea P, Saeedi P, Dahlquist G, Soltesz G, et al. Worldwide estimates of incidence, prevalence and mortality of type 1 diabetes in children and adolescents: Results from the International Diabetes Federation Diabetes Atlas, 9th edition. Diabetes Res Clin Pract. 2019;157:107842. https://doi.org/10.1016/j.diabres.2019.107842
3. DiMeglio LA, Evans-Molina C, Oram RA. Type 1 diabetes. Lancet. 2018;391(10138):2449-62. https://doi.org/10.1016/S0140-6736(18)31320-5
4. American Diabetes Association. 6. Glycemic Targets: Standards of Medical Care in Diabetes—2022. Diabetes Care. 2021;45(Supplement_1):S83-S96. https://doi.org/10.2337/dc22-S006
5. The National Board of Health and Welfare. Nationella riktlinjer för diabetesvård - Stöd för styrning och ledning. Report No.: 978-91-75555-476-1. Socialstyrelsen 2018 www.socialstyrelsen.se
6. The Swedish National Diabetes Register. Nationella Diabetesregistrets årsrapport 2021. www.ndr.nu
7. Shah VN, Grimsmann JM, Foster NC, Dost A, Miller KM, Pavel M, et al. Undertreatment of cardiovascular risk factors in the type 1 diabetes exchange clinic network (United States) and the prospective diabetes follow-up (Germany/Austria) registries. Diabetes Obes Metab. 2020;22(9):1577-85. https://doi.org/10.1111/dom.14069
8. Renard E, Ikegami H, Daher Vianna AG, Pozzilli P, Brette S, Bosnyak Z, et al. The SAGE study: Global observational analysis of glycaemic control, hypoglycaemia and diabetes management in T1DM. Diabetes Metab Res Rev. 2021;37(7):e3430. https://doi.org/10.1002/dmrr.3430
9. Funnell MM, Bootle S, Stuckey HL. The diabetes attitudes, wishes and needs second study. Clin Diabetes. 2015;33(1):32-6. https://doi.org/10.2337/diaclin.33.1.32
10. Reddy M, Rilstone S, Cooper P, Oliver NS. Type 1 diabetes in adults: supporting self management. BMJ. 2016;352:i998. https://doi.org/10.1136/bmj.i998
11. Iqbal A, Novodvorsky P, Heller SR. Recent Updates on Type 1 Diabetes Mellitus Management for Clinicians. Diabetes Metab J. 2018;42(1):3-18. https://doi.org/10.4093/dmj.2018.42.1.3
12. Jull J, Witteman HO, Ferne J, Yoganathan M, Stacey D. Adult-Onset Type 1 Diabetes: A Qualitative Study of Decision-Making Needs. Can J Diabetes. 2016;40(2):164-9. https://doi.org/10.1016/j.jcjd.2015.09.080
13. Fisher L, Hessler D, Polonsky W, Strycker L, Masharani U, Peters A. Diabetes distress in adults with type 1 diabetes: Prevalence, incidence and change over time. J Diabetes Complications. 2016;30(6):1123-8. https://doi.org/10.1016/j.jdiacomp.2016.03.032
14. Sturt J, Dennick K, Due-Christensen M, McCarthy K. The detection and management of diabetes distress in people with type 1 diabetes. Curr Diab Rep. 2015;15(11):101. https://doi.org/10.1007/s11892-015-0660-z
15. Roy T, Lloyd CE. Epidemiology of depression and diabetes: a systematic review. J Affect Disord. 2012;142 Suppl:S8-21. https://doi.org/10.1016/S0165-0327(12)70004-6
16. van Dooren FE, Nefs G, Schram MT, Verhey FR, Denollet J, Pouwer F. Depression and risk of mortality in people with diabetes mellitus: a systematic review and meta-analysis. PloS one. 2013;8(3):e57058. https://doi.org/10.1371/journal.pone.0057058
17. de Groot M, Golden SH, Wagner J. Psychological conditions in adults with diabetes. Am Psychol. 2016;71(7):552-62. https://doi.org/10.1037/a0040408
18. Hessler DM, Fisher L, Polonsky WH, Masharani U, Strycker LA, Peters AL, et al. Diabetes distress is linked with worsening diabetes management over time in adults with Type 1 diabetes. Diabet Med. 2017;34(9):1228-34. https://doi.org/10.1111/dme.13381
19. Melin EO, Thunander M, Svensson R, Landin-Olsson M, Thulesius HO. Depression, obesity, and smoking were independently associated with inadequate glycemic control in patients with type 1 diabetes. Eur J Endocrinol. 2013;168(6):861-9. https://doi.org/10.1530/EJE-13-0137

For peer review only - http://bmjopen.bmj.com/site/about/guidelines.xhtml

20. Jones A, Vallis M, Pouwer F. If it does not significantly change HbA1c levels why should we waste time on it? A plea for the prioritization of psychological well-being in people with diabetes. Diabet Med. 2015;32(2):155-63. https://doi.org/10.1111/dme.12620
21. Holt RIG, DeVries JH, Hess-Fischl A, Hirsch IB, Kirkman MS, Klupa T, et al. The management of type 1 diabetes in adults. A consensus report by the American Diabetes Association (ADA) and the European Association for the Study of Diabetes (EASD). Diabetologia. 2021;64(12):2609-52. https://doi.org/10.1007/s00125-021-05568-3
22. Fenn K, Byrne, M. . The key principals of cognitive behaviour therapy. InnovAiT: Education and inspiration for general practice. 2013;6(9). https://doi.org/10.1177/1755738012471029
23. Lepping P, Whittington R, Sambhi RS, Lane S, Poole R, Leucht S, et al. Clinical relevance of findings in trials of CBT for depression. Eur Psychiatry 2017;45:207-11. https://doi.org/10.1016/j.eurpsy.2017.07.003
24. Uchendu C, Blake H. Effectiveness of cognitive-behavioural therapy on glycaemic control and psychological outcomes in adults with diabetes mellitus: a systematic review and meta-analysis of randomized controlled trials. Diabet Med. 2017;34(3):328-39. https://doi.org/10.1111/dme.13195
25. Amsberg S, Anderbro T, Wredling R, Lisspers J, Lins PE, Adamson U, et al. A cognitive behavior therapy-based intervention among poorly controlled adult type 1 diabetes patients--a randomized controlled trial. Patient Educ Couns. 2009;77(1):72-80. https://doi.org/10.1016/j.pec.2009.01.015
26. Hayes S, Strosahl K, Wilson K. Acceptance and Commitment Therapy: The Process and Practice of Mindful Change. New York: Guilford Press 2011.
27. Hayes SC, Levin ME, Plumb-Vilardaga J, Villatte JL, Pistorello J. Acceptance and Commitment Therapy and Contextual Behavioral Science: Examining the Progress of a Distinctive Model of Behavioral and Cognitive Therapy. Behav Ther. 2013;44(2):180-98. https://doi.org/10.1016/j.beth.2009.08.002
28. Ruiz F. A review of Acceptance and Commitment Therapy (ACT) empirical evidence: Correlational, experimental psychopathology, component and outcome studies. International Journal of Psychology and Psychological Therapy. 2010;10:125-62.
29. Kashdan TB, Rottenberg J. Psychological flexibility as a fundamental aspect of health. Clin Psychol Rev. 2010;30(7):865-78. https://doi.org/10.1016/j.cpr.2010.03.001
30. A-Tjack J, Davis ML, Morina N, Powers MB, Smits JA, Emmelkamp PM. A meta-analysis of the efficacy of acceptance and commitment therapy for clinically relevant mental and physical health problems. Psychother Psychosom. 2015;84(1):30-6. https://doi.org/10.1159/000365764
31. Graham CD, Gouick J, Krahé C, Gillanders D. A systematic review of the use of Acceptance and Commitment Therapy (ACT) in chronic disease and long-term conditions. Clin Psychol Rev. 2016;46:46-58. https://doi.org/10.1016/j.cpr.2016.04.009
32. Nicholas JA, Yeap BB, Cross D, Burkhardt MS. Psychological flexibility is associated with less diabetes distress and lower glycated haemoglobin in adults with type 1 diabetes. Intern Med J. 2022;52(6):952-8. https://doi.org/10.1111/imj.15250
33. Sakamoto R, Ohtake Y, Kataoka Y, Matsuda Y, Hata T, Otonari J, et al. The efficacy of acceptance and commitment therapy for people with type 2 diabetes: Systematic review and meta-analysis. J Diabetes Investig. 2021. https://doi.org/10.1111/jdi.13658
34. Bendig E, Bauereiss N, Schmitt A, Albus P, Baumeister H. ACTonDiabetes-a guided psychological internet intervention based on Acceptance and Commitment Therapy (ACT) for adults living with type 1 or 2 diabetes: results of a randomised controlled feasibility trial. BMJ open. 2021;11(7):e049238-e. https://doi.org/10.1136/bmjopen-2021-049238
35. Amsberg S, Wijk I, Livheim F, Toft E, Johansson UB, Anderbro T. Acceptance and commitment therapy (ACT) for adult type 1 diabetes management: study protocol for a randomised controlled trial. BMJ Open. 2018;8(11):e022234. https://doi.org/10.1136/bmjopen-2018-022234

For peer review only - http://bmjopen.bmj.com/site/about/guidelines.xhtml

36. Livheim F. ACT treatment for youth – a contextual behavioral approach. [dissertation] Stockholm: Karolinska Institutet 2019. https://openarchive.ki.se/xmlui/bitstream/handle/10616/46394/Thesis_Fredrik_Livheim.pdf?sequence=3&isAllowed=y
37. Jeffcoate SL. Diabetes control and complications: the role of glycated haemoglobin, 25 years on. Diabet Med. 2004;21(7):657-65. https://doi.org/10.1046/j.1464-5491.2003.01065.x
38. American Diabetes Association. 6. Glycemic Targets: Standards of Medical Care in Diabetes—2021. Diabetes Care. 2020;44(Supplement_1):S73-S84. https://doi.org/10.2337/dc21-S006
39. Guadalupe Vargas M, Pazmiño Gomez BJ, Vera Lorenti FE, Álvarez Condo GM, Rodas Neira EI, Veron D, et al. Assessment of two glycated hemoglobin immunoassays. Endocrinol Diabetes Nutr (English ed). 2020;67(5):297-303. https://doi.org/10.1016/j.endinu.2019.09.011
40. Healthineers S. DCA Vantage Analyzer: Siemens Healthineers; [cited 2022 24-10]. Available from: https://www.siemens-healthineers.com/se/diabetes/diabetes/dca-vantage-analyzer
41. Lovibond PF, Lovibond SH. The structure of negative emotional states: comparison of the Depression Anxiety Stress Scales (DASS) with the Beck Depression and Anxiety Inventories. Behav Res Ther. 1995;33(3):335-43. https://doi.org/10.1016/0005-7967(94)00075-u
42. Alfonsson S, Wallin E, Maathz P. Factor structure and validity of the Depression, Anxiety and Stress Scale-21 in Swedish translation. J Psychiatr Ment Health Nurs. 2017;24(2-3):154-62. https://doi.org/10.1111/jpm.12363
43. Cox DJ, Irvine A, Gonder-Frederick L, Nowacek G, Butterfield J. Fear of hypoglycemia: quantification, validation, and utilization. Diabetes Care. 1987;10(5):617-21. https://doi.org/10.2337/diacare.10.5.617
44. Anderbro T, Amsberg S, Wredling R, Lins PE, Adamson U, Lisspers J, et al. Psychometric evaluation of the Swedish version of the Hypoglycaemia Fear Survey. Patient Educ Couns. 2008;73(1):127-31. https://doi.org/10.1016/j.pec.2008.03.022
45. Polonsky WH, Anderson BJ, Lohrer PA, Welch G, Jacobson AM, Aponte JE, et al. Assessment of diabetes-related distress. Diabetes Care. 1995;18(6):754-60. https://doi.org/10.2337/diacare.18.6.754
46. Hermanns N, Kulzer B, Krichbaum M, Kubiak T, Haak T. How to screen for depression and emotional problems in patients with diabetes: comparison of screening characteristics of depression questionnaires, measurement of diabetes-specific emotional problems and standard clinical assessment. Diabetologia. 2006;49(3):469-77. https://doi.org/10.1007/s00125-005-0094-2
47. Welch GW, Jacobson AM, Polonsky WH. The Problem Areas in Diabetes Scale. An evaluation of its clinical utility. Diabetes Care. 1997;20(5):760-6. https://doi.org/10.2337/diacare.20.5.760
48. Amsberg S, Wredling R, Lins PE, Adamson U, Johansson UB. The psychometric properties of the Swedish version of the Problem Areas in Diabetes Scale (Swe-PAID-20): scale development. Int J Nurs Stud. 2008;45(9):1319-28. https://doi.org/10.1016/j.ijnurstu.2007.09.010
49. Toobert DJ, Hampson SE, Glasgow RE. The summary of diabetes self-care activities measure: results from 7 studies and a revised scale. Diabetes Care. 2000;23(7):943-50. https://doi.org/10.2337/diacare.23.7.943
50. Hayes S, Strosahl K, Wilson K, Bissett RT, Pistorello J, Toarmino D, et al. Measuring experiential avoidance: A preliminary test of a working model. The Psychological record. 2004;54:553-78. https://doi.org/10.1007/BF03395492
51. Bond FW, Hayes SC, Baer RA, Carpenter KM, Guenole N, Orcutt HK, et al. Preliminary psychometric properties of the Acceptance and Action Questionnaire-II: a revised measure of psychological inflexibility and experiential avoidance. Behav Ther. 2011;42(4):676-88. https://doi.org/10.1016/j.beth.2011.03.007
52. Lundgren T, Parling T. Swedish Acceptance and Action Questionnaire (SAAQ): a psychometric evaluation. Cogn Behav Ther. 2017;46(4):315-26. https://doi.org/10.1080/16506073.2016.1250228

For peer review only - http://bmjopen.bmj.com/site/about/guidelines.xhtml

53. Gregg JA, Callaghan GM, Hayes SC, Glenn-Lawson JL. Improving diabetes self-management through acceptance, mindfulness, and values: a randomized controlled trial. J Consult Clin Psychol. 2007;75(2):336-43. https://doi.org/10.1037/0022-006X.75.2.336
54. Priebe S, Huxley P, Knight S, Evans S. Application and results of the Manchester Short Assessment of Quality of Life (MANSA). Int J Soc psychiatry. 1999;45(1):7-12. https://doi.org/10.1177/002076409904500102
55. Björkman T, Svensson B. Quality of life in people with severe mental illness. Reliability and validity of the Manchester Short Assessment of Quality of Life (MANSA). Nord J Psychiatry. 2005;59(4):302-6. https://doi.org/10.1080/08039480500213733
56. R Core Team. R: A language and environment for statistical computing. Vienna, Austria.: R Foundation for Statistical Computing; 2019 [Available from: https://www.R-project.org.]
57. Winkley K, Upsher R, Stahl D, Pollard D, Brennan A, Heller S, et al. Systematic review and meta-analysis of randomized controlled trials of psychological interventions to improve glycaemic control in children and adults with type 1 diabetes. Diabet Med. 2020;37(5):735-46. https://doi.org/10.1111/dme.14264
58. Jenkinson E, Knoop I, Hudson JL, Moss-Morris R, Hackett RA. The effectiveness of cognitive behavioural therapy and third-wave cognitive behavioural interventions on diabetes-related distress: A systematic review and meta-analysis. Diabet Med. 2022;39(11):e14948. https://doi.org/10.1111/dme.14948
59. Snoek FJ, van der Ven NC, Twisk JW, Hogenelst MH, Tromp-Wever AM, van der Ploeg HM, et al. Cognitive behavioural therapy (CBT) compared with blood glucose awareness training (BGAT) in poorly controlled Type 1 diabetic patients: long-term effects on HbA moderated by depression. A randomized controlled trial. Diabet Med. 2008;25(11):1337-42. https://doi.org/10.1111/j.1464-5491.2008.02595.x
60. van der Ven NC, Hogenelst MH, Tromp-Wever AM, Twisk JW, van der Ploeg HM, Heine RJ, et al. Short-term effects of cognitive behavioural group training (CBGT) in adult Type 1 diabetes patients in prolonged poor glycaemic control. A randomized controlled trial. Diabet Med. 2005;22(11):1619-23. https://doi.org/10.1111/j.1464-5491.2005.01691.x
61. Yang X, Li Z, Sun J. Effects of Cognitive Behavioral Therapy–Based Intervention on Improving Glycaemic, Psychological, and Physiological Outcomes in Adult Patients With Diabetes Mellitus: A Meta-Analysis of Randomized Controlled Trials. Front Psychiatry. 2020;11(711). https://doi.org/10.3389/fpsyt.2020.00711
62. Alho I, Lappalainen P, Muotka J, Lappalainen R. Acceptance and commitment therapy group intervention for adolescents with type 1 diabetes: A randomized controlled trial. J Contextual Behav Sci. 2022;25:153-61. https://doi.org/10.1016/j.jcbs.2022.08.002
63. Chan AW, Tetzlaff JM, Altman DG, Laupacis A, Gøtzsche PC, Krleža-Jerić K, et al. SPIRIT 2013 statement: defining standard protocol items for clinical trials. Ann Intern Med. 2013;158(3):200-7. https://doi.org/10.7326/0003-4819-158-3-201302050-00583
64. Fledderus M, Bohlmeijer ET, Smit F, Westerhof GJ. Mental health promotion as a new goal in public mental health care: a randomized controlled trial of an intervention enhancing psychological flexibility. Am J Public Health. 2010;100(12):2372. https://doi.org/10.2105/AJPH.2010.196196
65. Cherry KM, Hoeven EV, Patterson TS, Lumley MN. Defining and measuring "psychological flexibility": A narrative scoping review of diverse flexibility and rigidity constructs and perspectives. Clin Psychol Rev. 2021;84:101973. https://doi.org/10.1016/j.cpr.2021.101973
66. Malins S, Biswas S, Rathbone J, Vogt W, Pye N, Levene J, et al. Reducing dropout in acceptance and commitment therapy, mindfulness-based cognitive therapy, and problem-solving therapy for chronic pain and cancer patients using motivational interviewing. Br J Clin Psychol. 2020;59(3):424-38. https://doi.org/10.1111/bjc.12254

For peer review only - http://bmjopen.bmj.com/site/about/guidelines.xhtml

67. Fernandez E, Salem D, Swift J, Ramtahal N. Meta-Analysis of Dropout From Cognitive Behavioral Therapy: Magnitude, Timing, and Moderators. J Consult Clin Psychol. 2015;83. https://doi.org/10.1037/ccp0000044
68. Ong CW, Lee EB, Twohig MP. A meta-analysis of dropout rates in acceptance and commitment therapy. Behav Res Ther. 2018;104:14-33. https://doi.org/10.1016/j.brat.2018.02.004
69. Craig P, Dieppe P, Macintyre S, Michie S, Nazareth I, Petticrew M. Developing and evaluating complex interventions: the new Medical Research Council guidance. BMJ. 2008;337:a1655. https://doi.org/10.1136/bmj.a1655

70. Ding K, Reynolds CM, Driscoll KA, Janicke DM. The Relationship Between Executive Functioning, Type 1 Diabetes Self-Management Behaviors, and Glycemic Control in Adolescents and Young Adults. Curr Diab Rep. 2021;21(3):10. https://doi.org/10.1007/s11892-021-01379-3

71. Dindo L, Van Liew JR, Arch JJ. Acceptance and Commitment Therapy: A Transdiagnostic Behavioral Intervention for Mental Health and Medical Conditions. Neurotherapeutics. 2017;14(3):546-53. https://doi.org/10.1007/s13311-017-0521-3

72. Todd PJ, Edwards F, Spratling L, Patel NH, Amiel SA, Sturt J, et al. Evaluating the relationships of hypoglycaemia and HbA1c with screening-detected diabetes distress in type 1 diabetes. Endocrinology, diabetes & metabolism. 2018;1(1):e00003. https://doi.org/10.1002/edm2.3

For peer review only

Figure 1: Flow diagram of recruitment, randomisation and attrition

For peer review only - http://bmjopen.bmj.com/site/about/guidelines.xhtml

HbA1c, glycated haemoglobin.

Figure 2: Mean values of HbA1c over two years by group affiliation

ACT, acceptance and commitment therapy; HbA1c, glycated haemoglobin; CI, confidence interval.

Figure 3: Mean values of AAQ-II score over two years by group affiliation

AAQ, Acceptance and Action Questionnaire; HbA1c, glycated haemoglobin; CI, confidence interval.

For peer review only

For peer review only - http://bmjopen.bmj.com/site/about/guidelines.xhtml

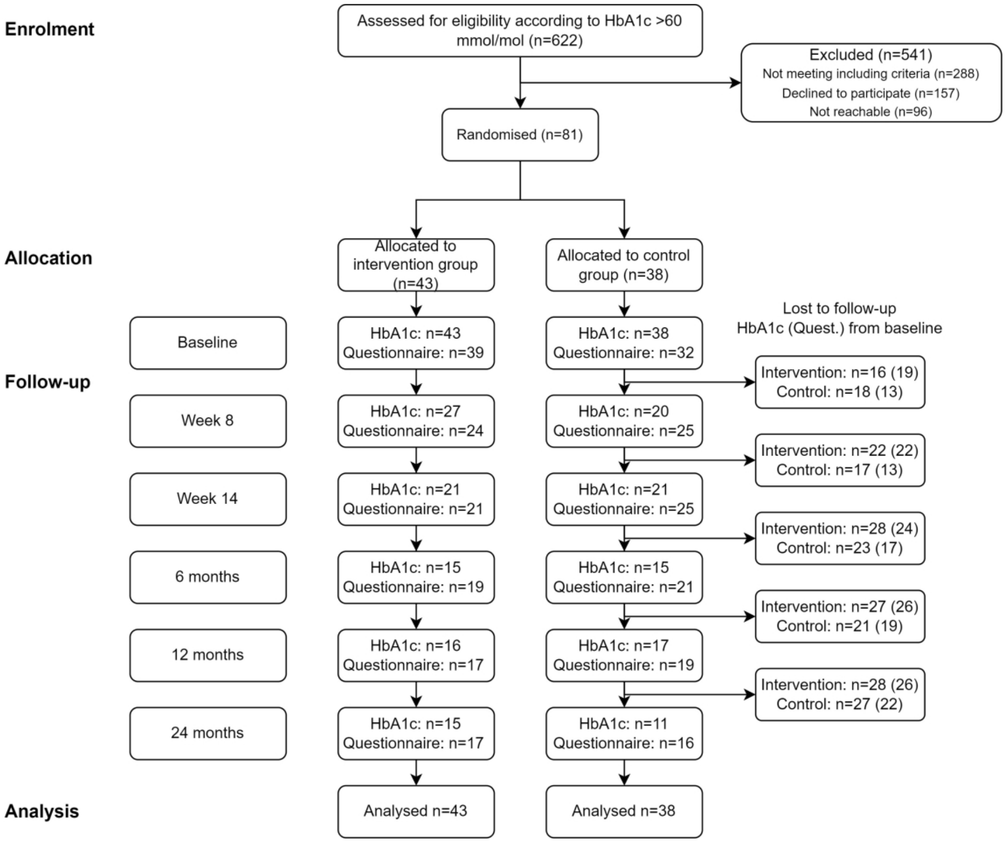

Flow diagram of recruitment, randomisation and attrition
HbA1c, glycated haemoglobin.

1058x882mm (72 x 72 DPI)

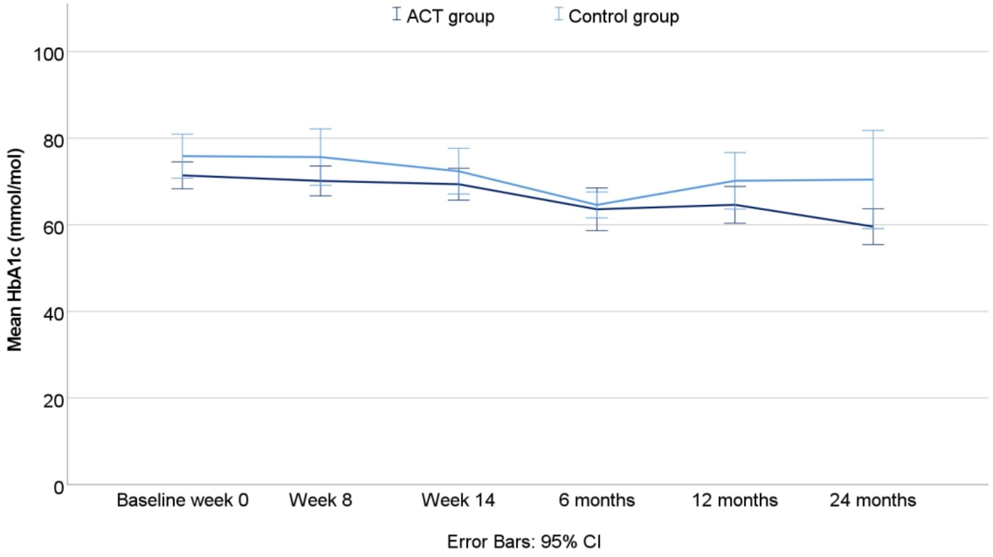

Figure 2: Mean values of HbA1c over two years by group affiliation
ACT, acceptance and commitment therapy; HbA1c, glycated haemoglobin; CI, confidence interval.

1219x713mm (72 x 72 DPI)

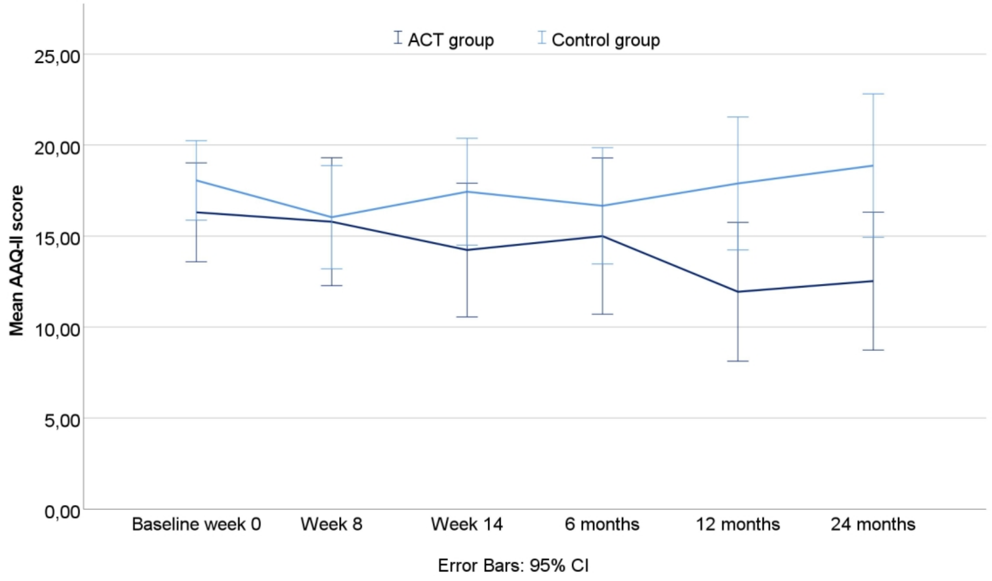

Figure 3: Mean values of AAQ-II score over two years by group affiliation
AAQ, Acceptance and Action Questionnaire; HbA1c, glycated haemoglobin; CI, confidence interval.

1245x727mm (72 x 72 DPI)

Supplementary Table 1: Estimates of the adherence among the instructors (n=2) to the ACT programme.

	Group A (n=5) s 3 median (range)	Instructors s 3 median (range)	Group A (n=5) s 7 median (range)	Instructors s 7 median (range)	Group B (n=3) s 3 median (range)	Instructors s 3 median (range)
Review of preceding sessions	8 (6-9)	10 (10-10)	9 (9-10)	8 (8-8)	7 (6-9)	9 (9-9)
Feedback on homework	7 (5-10)	7.5 (7-8)	9 (8-10)	6.5 (6-7)	8 (7-10)	8 (7-9)
The Compass of life	10 (7-10)	7.5 (7-8)	9 (9-10)	8 (8-8)	9 (7-10)	9.5 (9-10)
Specific exercise	9.5 (8-10)	8.5 (8-9)	10 (8-10)	9 (9-9)	9 (6-10)	9.5 (9-10)

Estimations on a scale 1-10 (0= "this has not been discussed/brought up", to 10 = "This was covered thoroughly".)
Examples of items from the adherence evaluation. s 3: session 3, s 7: session 7

Supplementary Table 2: Estimates of the adherence among participants in two groups to the ACT programme.

	Group A (n=5) s 3 median(range)	Group A (n=5) s 7 median(range)	Group B (n=3) s 3 median(range)
Made changes in life[1]	2 (1-2)	2.5 (2-3)	2 (2-3)
App use (mindfulness)[2]	0 (0-2)	1 (0-1)	2 (0-10)
Mindfulness daily life[3]	3 (2-4)	3.5 (2-4)	4 (3-4)

1. Have you since the course started, decided to change something/take steps in your life direction? 1=No, 2=I have decided and made a small step/change,3=I have decided and made several changes/steps.

2. How many times per week did you practise mindfulness with the app "I´m here now"?

3. How often do you practise mindfulness in your daily life? 1=Never,2=Seldom, 3=Once a week/sometimes,4=Once per day, 5=Several times a day

s 3: session 3, s 7: session 7

For peer review only

Supplementary Table 3: Observed mean value at each time point for outcome measurements and linear mixed model analysis with testing for interaction effects of time and ACT intervention on HbA1c, PAID, DASS21 and AAQ-II.

							Mixed model analysis with interaction		
	**Week 0**	**Week 8**	**Week 14**	**6 months**	**12 months**	**24 months**	**Fixed effects**	**Estimate (95% CI)**	**Interaction effect χ^2 (p value)**
**HbA1c** mmol/mol									
ACT							Intercept	75.22 (71.43, 79.01)	
N	43	27	21	15	16	15	ACT	-3.71 (-8.90, 1.49)	
Mean (SD)	71.4 (10.1)	70.1 (8.9)	69.4 (8.3)	63.6 (9.5)	64.6 (8.4)	59.6 (8.0)	Time	-0.05 (-0.09, -0.01)	
Control							ACT*time	-0.02 (-0.07, 0.03)	
N	38	20	21	15	17	11			
Mean (SD)	75.9 (15.4)	75.7 (14.4)	72.4 (11.9)	64.6 (5.7)	70.2 (13.3)	70.5 (18.6)			
P-value	0.315	0.281	0.227	0.677	0.181	0.132			0.49 (0.485)
**PAID**									
ACT							Intercept	43.52 (37.78, 49.29)	
N	39	24	21	19	17	17	ACT	-9.40 (-17.36, -1.46)	
Mean (SD)	36.6 (18.1)	32.6 (19.7)	28.7 (18.7)	31.1 (18.5)	28.6 (16.0)	27.4 (18.6)	Time	-0.05 (-0.10, -0.01)	
Control							ACT*time	-0.01 (-0.08, 0.05)	
N	32	25	25	21	19	16			
Mean (SD)	42.9 (15.9)	39.8 (20.7)	41.6 (19.2)	34.9 (20.9)	36.2 (16.6)	33.4 (17.7)			
P value	0.229	0.322	0.034	0.533	0.188	0.279			0.22(0.642)
**DASS-S**									
ACT							Intercept	16.53 (13.63, 19.44)	
N	39	24	21	19	17	17	ACT	-4.90 (-8.92, -0.89)	
Mean (SD)	12.5 (8.4)	12.5 (9.2)	9.8 (8.3)	12.8 (10.4)	9.4 (6.3)	10.7 (6.7)	Time	-0.01 (-0.03, 0.02)	
Control							ACT*time	-0.01 (-0.04, 0.03)	
N	32	25	25	21	19	16			
Mean (SD)	16.8 (11.1)	14.5 (10.7)	15.8 (9.7)	13.0 (10.9)	14.3 (11.3)	14.1 (10.7)			

For peer review only

For peer review only - http://bmjopen.bmj.com/site/about/guidelines.xhtml

P value	0.141	0.534	0.036	0.978	0.226	0.458				0.11(0.743)
**DASS-A**										
ACT								Intercept	7.54 (5.44, 9.64)	
N	39	24	21	19	17	17		ACT	-1.06 (-3.97, 1.85)	
Mean (SD)	6.8 (6.8)	5.8 (7.1)	5.4 (6.3)	7.3 (8.3)	4.2 (4.0)	4.6 (7.9)		Time	0.01 (-0.01, 0.03)	
Control								ACT*time	-0.02 (-0.05, 0.01)	
N	32	25	25	21	19	16				
Mean (SD)	8.2 (7.1)	6.6 (7.0)	6.9 (6.0)	6.5 (7.0)	8.0 (6.7)	7.1 (6.2)				
P value	0.302	0.460	0.283	0.763	0.042	0.063				1.97(0.161)
**DASS-D**										
ACT								Intercept	13.10 (9.87, 16.35)	
N	39	24	21	19	17	17		ACT	-2.53 (-7.03, 1.95)	
Mean (SD)	11.7 (11.4)	10.9 (11.0)	6.9 (9.4)	11.6 (12.8)	6.0 (7.4)	10.0 (7.5)		Time	0.02 (-0.01, 0.06)	
Control								ACT*time	-0.03 (-0.08, 0.01)	
N	32	25	25	21	19	16				
Mean (SD)	12.6 (10.3)	11.3 (9.0)	14.1 (10.0)	11.1 (11.7)	14.8 (11.3)	14.1 (10.5)				
P value	0.486	0.672	0.015	0.989	0.011	0.261				2.00(0.157)
**AAQ**										
ACT								Intercept	17.69 (15.34, 20.05)	
N	39	24	21	19	17	17		ACT	-1.95 (-5.20, 1.30)	
Mean (SD)	16.3 (8.4)	15.8 (8.5)	14.2 (8.3)	15.0 (9.3)	11.9 (7.8)	12.5 (7.8)		Time	0.02 (0.00, 0.04)	
Control								ACT*time	-0.05 (-0.08, -0.02)	
N	32	25	25	21	19	16				
Mean (SD)	18.1 (6.1)	16.0 (7.0)	17.4 (7.3)	16.7 (7.2)	17.9 (7.9)	18.9 (7.8)				
P value	0.325	0.749	0.095	0.356	0.016	0.013				12.63 (<0.001)

*DASS-S-stress, DASS-A-anxiety, DASS-D-depression

ACT, acceptance and commitment therapy; SD, standard deviation; CI, confidence interval; HbA1c, glycated haemoglobin; PAID, Problem Areas in Diabetes Scale; DASS, Depression, Anxiety, Stress Scale; AAQ, Acceptance and Action Questionnaire.

For peer review only

Supplementary Table 4: Observed mean value at each time point for outcome measurements and linear mixed model analysis with testing for interaction effects of time and ACT intervention on HFS, AADQ, MANSA and SDSCA

**HFS-B**										
ACT								Intercept	20.27 (18.61, 21.93)	
N	39	24	21	19	17	17		ACT	-3.14 (-5.44, -0.85)	
Mean (SD)	18.1 (4.9)	15.8 (5.2)	15.7 (4.6)	15.9 (4.4)	15.4 (4.5)	16.7 (6.8)		Time	-0.02 (-0.03, 0.00)	
Control								ACT*time	0.01 (-0.01, 0.03)	
N	32	25	25	21	19	16				
Mean (SD)	20.4 (5.3)	19.5 (4.9)	19.5 (6.7)	18.7 (6.0)	18.7 (5.8)	19.6 (5.3)				
P-value	0.030	0.013	0.021	0.162	0.177	0.139				0.55 (0.457)
**HFS-W**										
ACT								Intercept	17.00 (14.02, 19.99)	
N	39	24	21	19	17	17		ACT	-2.06 (-6.20, 2.05)	
Mean (SD)	16.7 (10.2)	14.2 (8.7)	12.5 (6.6)	12.0 (6.7)	11.6 (6.5)	12.6 (7.7)		Time	-0.01 (-0.04, 0.01)	
Control								ACT*time	-0.01 (-0.04, 0.02)	
N	32	25	25	21	19	16				
Mean (SD)	16.4 (9.3)	14.3 (8.8)	16.1 (8.7)	13.5 (11.4)	14.1 (8.7)	13.5 (8.7)				
P-value	0.986	0.888	0.182	0.957	0.526	0.843				0.65 (0.420)
**AADQ**										
ACT								Intercept	53.40 (50.50, 56.30)	
N	39	24	21	19	17	17		ACT	4.80 (0.80, 8.82)	
Mean (SD)	57.4 (8.8)	58.7 (7.6)	59.6 (7.7)	59.0 (9.2)	62.1 (8.5)	61.2 (8.3)		Time	0.03 (0.01, 0.05)	
Control								ACT*time	0.01 (-0.02, 0.04)	
No.	32	25	25	21	19	16				

For peer review only

Mean (SD)	52.6 (11.3)	54.3 (11.3)	54.5 (9.6)	58.3 (7.8)	56.5 (7.1)	57.8 (5.9)				
P value	0.049	0.207	0.047	0.560	0.022	0.073				0.34(0.562)
**MANSA**										
ACT								Intercept	4.65 (4.38, 4.91)	
N	39	24	21	19	17	17		ACT	0.44 (0.07, 0.80)	
Mean (SD)	5.0 (0.9)	5.1 (0.8)	5.3 (0.8)	5.1 (0.9)	5.3 (0.6)	5.3 (0.9)		Time	0.00 (0.00, 0.00)	
Control								ACT*time	0.00 (0.00, 0.01)	
N	32	25	25	21	19	16				
Mean (SD)	4.7 (0.7)	4.7 (1.0)	4.5 (0.9)	4.8 (0.9)	4.8 (0.9)	4.5 (0.8)				
P value	0.093	0.118	0.006	0.266	0.038	0.015				5.79(0.016)
**SDSCA-GD**										
ACT								Intercept	4.14 (3.61, 4.67)	
No.	39	24	21	19	17	17		ACT	0.75 (0.02, 1.48)	
Mean (SD)	5.0 (1.6)	4.7 (1.8)	4.5 (1.7)	4.9 (1.6)	5.1 (1.5)	5.1 (1.5)		Time	0.01 (0.00, 0.01)	
Control								ACT*time	0.00 (-0.01, 0.01)	
No.	32	25	25	21	19	16				
Mean (SD)	4.1 (1.9)	3.8 (2.1)	4.4 (1.8)	4.9 (1.4)	4.9 (1.8)	4.7 (1.6)				
P value	0.062	0.104	0.722	0.743	0.784	0.388				0.01(0.924)
**SDSCA- SD**										
ACT								Intercept	3.66 (3.18, 4.15)	
N	39	24	21	19	17	17		ACT	0.21 (-0.46, 0.88)	
Mean (SD)	3.8 (1.8)	4.1 (1.5)	3.6 (1.4)	4.5 (1.2)	4.2 (1.6)	4.1 (1.7)		Time	0.00 (-0.01, 0.00)	
Control								ACT*time	0.00 (0.00, 0.01)	
No.	32	25	25	21	19	16				

For peer review only - http://bmjopen.bmj.com/site/about/guidelines.xhtml

Mean (SD)	3.5 (1.6)	3.7 (1.6)	3.7 (1.6)	4.2 (1.5)	3.7 (1.5)	3.6 (1.6)				
P value	0.313	0.415	0.706	0.405	0.425	0.269				1.37 (0.242)
**SDSCA-E**										
ACT								Intercept	2.62 (2.03, 3.22)	
No.	39	24	21	19	17	17		ACT	0.73 (-0.10, 1.55)	
Mean (SD)	3.2 (2.1)	4.2 (1.9)	3.2 (2.0)	3.8 (2.3)	4.1 (2.1)	3.6 (2.2)		Time	0.00 (0.00, 0.01)	
Control								ACT*time	0.00 (-0.01, 0.01)	
N	32	25	25	21	19	16				
Mean (SD)	2.5 (2.0)	2.7 (2.0)	2.5 (1.9)	2.9 (1.8)	3.2 (2.6)	2.9 (1.7)				
P value	0.174	0.010	0.186	0.253	0.245	0.536				0.10 (0.749)
**SDSCA -G**										
ACT								Intercept	5.37 (4.83, 5.91)	
N	39	24	21	19	17	17		ACT	0.84 (0.09, 1.59)	
Mean (SD)	6.1 (1.8)	6.3 (1.2)	6.3 (1.4)	6.6 (0.9)	6.9 (0.3)	6.8 (0.8)		Time	0.01 (0.00, 0.01)	
Control								ACT*time	0.00 (-0.01, 0.01)	
N	32	25	25	21	19	16				
Mean (SD)	5.4 (2.5)	5.5 (2.3)	5.7 (2.2)	6.0 (1.7)	6.0 (1.9)	6.7 (0.8)				
P value	0.423	0.300	0.613	0.265	0.117	0.924				0.23 (0.634)

ACT, acceptance and commitment therapy; SD, standard deviation; CI, confidence interval; HbA1c, glycated haemoglobin; Scale; HFS, Hypoglycaemia Fear Survey (HFS- W-Worry, HFS-B- Behaviour); AADQ, Acceptance Action Diabetes Questionnaire; MANSA, Manchester Short Assessment of Quality of life; SDSCA, Summary of Diabetes Self-Care Activities (SDSCA-GD-general diet, SDSCA-SD-specific diet, SDSCA-E-Exercise, SDSCA-G-Glucose monitoring).

For peer review only

Supplementary Table 5: ACT exposure (number of sessions attended) and proportion of participants with HbA1c measurement in each timepoint

**ACT exposure**	**Frequency** Intervention group (n =43)	**Frequency** Control group (n=38)
Zero sessions	8	-
One session	3	-
Two sessions	5	-
Three sessions	7	-
Four sessions	1	-
Five sessions	3	-
Six sessions	9	-
Seven sessions	7	-
**HbA1c measurement**		
Week 8, n (%)	27 (62.8)	20 (52.6)
Week 14, n (%)	21 (48.8)	21 (55.3)
6 months, n (%)	15 (34.9)	15 (39.5)
12 months, n (%)	16 (37.2)	17 (44.7)
24 months, (%)	15 (34.9)	11 (28.9)

ACT, acceptance and commitment therapy; HbA1c, glycated Haemoglobin.

For peer review only

CONSORT 2010 checklist of information to include when reporting a randomised trial*

Section/Topic	Item No	Checklist item	Reported on page No
**Title and abstract**			
	1a	Identification as a randomised trial in the title	Title page
	1b	Structured summary of trial design, methods, results, and conclusions (for specific guidance see CONSORT for abstracts)	1
**Introduction**			
Background and objectives	2a	Scientific background and explanation of rationale	2
	2b	Specific objectives or hypotheses	3
**Methods**			
Trial design	3a	Description of trial design (such as parallel, factorial) including allocation ratio	3-4
	3b	Important changes to methods after trial commencement (such as eligibility criteria), with reasons	-
Participants	4a	Eligibility criteria for participants	3
	4b	Settings and locations where the data were collected	4
Interventions	5	The interventions for each group with sufficient details to allow replication, including how and when they were actually administered	4
Outcomes	6a	Completely defined pre-specified primary and secondary outcome measures, including how and when they were assessed	4-5
	6b	Any changes to trial outcomes after the trial commenced, with reasons	-
Sample size	7a	How sample size was determined	3
	7b	When applicable, explanation of any interim analyses and stopping guidelines	-
Randomisation:			
Sequence generation	8a	Method used to generate the random allocation sequence	3
	8b	Type of randomisation; details of any restriction (such as blocking and block size)	3
Allocation concealment mechanism	9	Mechanism used to implement the random allocation sequence (such as sequentially numbered containers), describing any steps taken to conceal the sequence until interventions were assigned	3-4
Implementation	10	Who generated the random allocation sequence, who enrolled participants, and who assigned participants to interventions	3-4
Blinding	11a	If done, who was blinded after assignment to interventions (for example, participants, care providers, those	-

For peer review only

		assessing outcomes) and how	
	11b	If relevant, description of the similarity of interventions	-
Statistical methods	12a	Statistical methods used to compare groups for primary and secondary outcomes	6
	12b	Methods for additional analyses, such as subgroup analyses and adjusted analyses	-
**Results**			
Participant flow (a diagram is strongly recommended)	13a	For each group, the numbers of participants who were randomly assigned, received intended treatment, and were analysed for the primary outcome	7
	13b	For each group, losses and exclusions after randomisation, together with reasons	7&10
Recruitment	14a	Dates defining the periods of recruitment and follow-up	3-4
	14b	Why the trial ended or was stopped	-
Baseline data	15	A table showing baseline demographic and clinical characteristics for each group	8-9
Numbers analysed	16	For each group, number of participants (denominator) included in each analysis and whether the analysis was by original assigned groups	7
Outcomes and estimation	17a	For each primary and secondary outcome, results for each group, and the estimated effect size and its precision (such as 95% confidence interval)	10-13
	17b	For binary outcomes, presentation of both absolute and relative effect sizes is recommended	-
Ancillary analyses	18	Results of any other analyses performed, including subgroup analyses and adjusted analyses, distinguishing pre-specified from exploratory	13
Harms	19	All important harms or unintended effects in each group (for specific guidance see CONSORT for harms)	-
**Discussion**			
Limitations	20	Trial limitations, addressing sources of potential bias, imprecision, and, if relevant, multiplicity of analyses	14
Generalisability	21	Generalisability (external validity, applicability) of the trial findings	14
Interpretation	22	Interpretation consistent with results, balancing benefits and harms, and considering other relevant evidence	13-14
**Other information**			
Registration	23	Registration number and name of trial registry	1
Protocol	24	Where the full trial protocol can be accessed, if available	3
Funding	25	Sources of funding and other support (such as supply of drugs), role of funders	15

*We strongly recommend reading this statement in conjunction with the CONSORT 2010 Explanation and Elaboration for important clarifications on all the items. If relevant, we also recommend reading CONSORT extensions for cluster randomised trials, non-inferiority and equivalence trials, non-pharmacological treatments, herbal interventions, and pragmatic trials. Additional extensions are forthcoming: for those and for up to date references relevant to this checklist, see www.consort-statement.org.

For peer review only - http://bmjopen.bmj.com/site/about/guidelines.xhtml
